# The Heme Biosynthetic Pathway of the Obligate *Wolbachia* Endosymbiont of *Brugia malayi* as a Potential Anti-filarial Drug Target

**DOI:** 10.1371/journal.pntd.0000475

**Published:** 2009-07-14

**Authors:** Bo Wu, Jacopo Novelli, Jeremy Foster, Romualdas Vaisvila, Leslie Conway, Jessica Ingram, Mehul Ganatra, Anita U. Rao, Iqbal Hamza, Barton Slatko

**Affiliations:** 1 Division of Molecular Parasitology, New England Biolabs, Ipswich, Massachusetts, United States of America; 2 Department of Animal and Avian Sciences and Department of Cell Biology and Molecular Genetics, University of Maryland, College Park, Maryland, United States of America; Washington University School of Medicine, United States of America

## Abstract

**Background:**

Filarial parasites (e.g., *Brugia malayi*, *Onchocerca volvulus*, and *Wuchereria bancrofti*) are causative agents of lymphatic filariasis and onchocerciasis, which are among the most disabling of neglected tropical diseases. There is an urgent need to develop macro-filaricidal drugs, as current anti-filarial chemotherapy (e.g., diethylcarbamazine [DEC], ivermectin and albendazole) can interrupt transmission predominantly by killing microfilariae (mf) larvae, but is less effective on adult worms, which can live for decades in the human host. All medically relevant human filarial parasites appear to contain an obligate endosymbiotic bacterium, *Wolbachia*. This alpha-proteobacterial mutualist has been recognized as a potential target for filarial nematode life cycle intervention, as antibiotic treatments of filarial worms harboring *Wolbachia* result in the loss of worm fertility and viability upon antibiotic treatments both *in vitro* and *in vivo*. Human trials have confirmed this approach, although the length of treatments, high doses required and medical counter-indications for young children and pregnant women warrant the identification of additional anti-*Wolbachia* drugs.

**Methods and Findings:**

Genome sequence analysis indicated that enzymes involved in heme biosynthesis might constitute a potential anti-*Wolbachia* target set. We tested different heme biosynthetic pathway inhibitors in *ex vivo B. malayi* viability assays and report a specific effect of N-methyl mesoporphyrin (NMMP), which targets ferrochelatase (FC, the last step). Our phylogenetic analysis indicates evolutionarily significant divergence between *Wolbachia* heme genes and their human homologues. We therefore undertook the cloning, overexpression and analysis of several enzymes of this pathway alongside their human homologues, and prepared proteins for drug targeting. *In vitro* enzyme assays revealed a ∼600-fold difference in drug sensitivities to succinyl acetone (SA) between *Wolbachia* and human 5′-aminolevulinic acid dehydratase (ALAD, the second step). Similarly, *Escherichia coli hemH* (FC) deficient strains transformed with human and *Wolbachia* FC homologues showed significantly different sensitivities to NMMP. This approach enables functional complementation in *E. coli* heme deficient mutants as an alternative *E. coli*-based method for drug screening.

**Conclusions:**

Our studies indicate that the heme biosynthetic genes in the *Wolbachia* of *B. malayi* (*w*Bm) might be essential for the filarial host survival. In addition, the results suggest they are likely candidate drug targets based upon significant differences in phylogenetic distance, biochemical properties and sensitivities to heme biosynthesis inhibitors, as compared to their human homologues.

## Author Summary

Human filarial nematodes are causative agents of elephantiasis and African river blindness, which are among the most debilitating tropical diseases. Currently used drugs mainly affect microfilariae (mf) and have less effect on adult filarial nematodes, which can live in the human host for more than a decade. Filariasis drug control strategy relies on recurrent mass drug administration for many years. Development of novel drugs is also urgently needed due to the threat of drug resistance occurrence. Most filarial worms harbor an obligate endosymbiotic bacterium, *Wolbachia*, whose presence has been identified as a potential drug target. Comparative genomics had suggested *Wolbachia* heme biosynthesis as a potential drug target, and we present an analysis of selected enzymes alongside their human homologues from several different aspects—gene phylogenetic analyses, *in vitro* enzyme kinetic and inhibition assays and heme-deficient *E. coli* complementation assays. We also conducted *ex vivo Brugia malayi* viability assays using heme pathway inhibitors. These experiments demonstrate that heme biosynthesis could be critical for filarial worm survival and thus is a potential anti-filarial drug target set.

## Introduction

Human filarial nematodes affect more than 150 million people worldwide with 1 billion people at risk in over 80 countries, and lead to some of the most debilitating tropical diseases, including elephantiasis and African river blindness [1],[2]. The current anti-filarial treatments e.g. DEC, ivermectin, albendazole (all suitable for lymphatic filariasis; ivermectin for onchocerciasis) interrupt the cycle of transmission of the causative filarial parasites *Brugia malayi*, *Onchocerca volvulus* and *Wuchereria bancrofti*, by predominantly killing microfilaria. However, a lower activity against adult worms, which can survive in human hosts for up to decades, is known. DEC and albendazole produce macrofilaricidal activity only after repeated rounds of mass drug administration (MDA) [3]. Since the current treatments have to be administered annually on a community-wide basis for many years to break the infection cycle, and drug resistance may be emerging [4],[5], there is still an urgent need to develop novel drugs (particularly macrofilaricidal). Numerous lines of evidence, in both laboratory and human trials, show that depletion of *Wolbachia* in filarial parasites by antibiotics (e.g. doxycycline, tetracycline) can kill adult worms in addition to affecting embryogenesis, mf output and worm development [6],[7],[8],[9],[10],[11],[12],[13]. These studies indicate that these vertically transmitted *Wolbachia* endosymbionts are indispensible for their filarial hosts and represent a promising therapeutic strategy for filariasis control.

**Figure 1:**
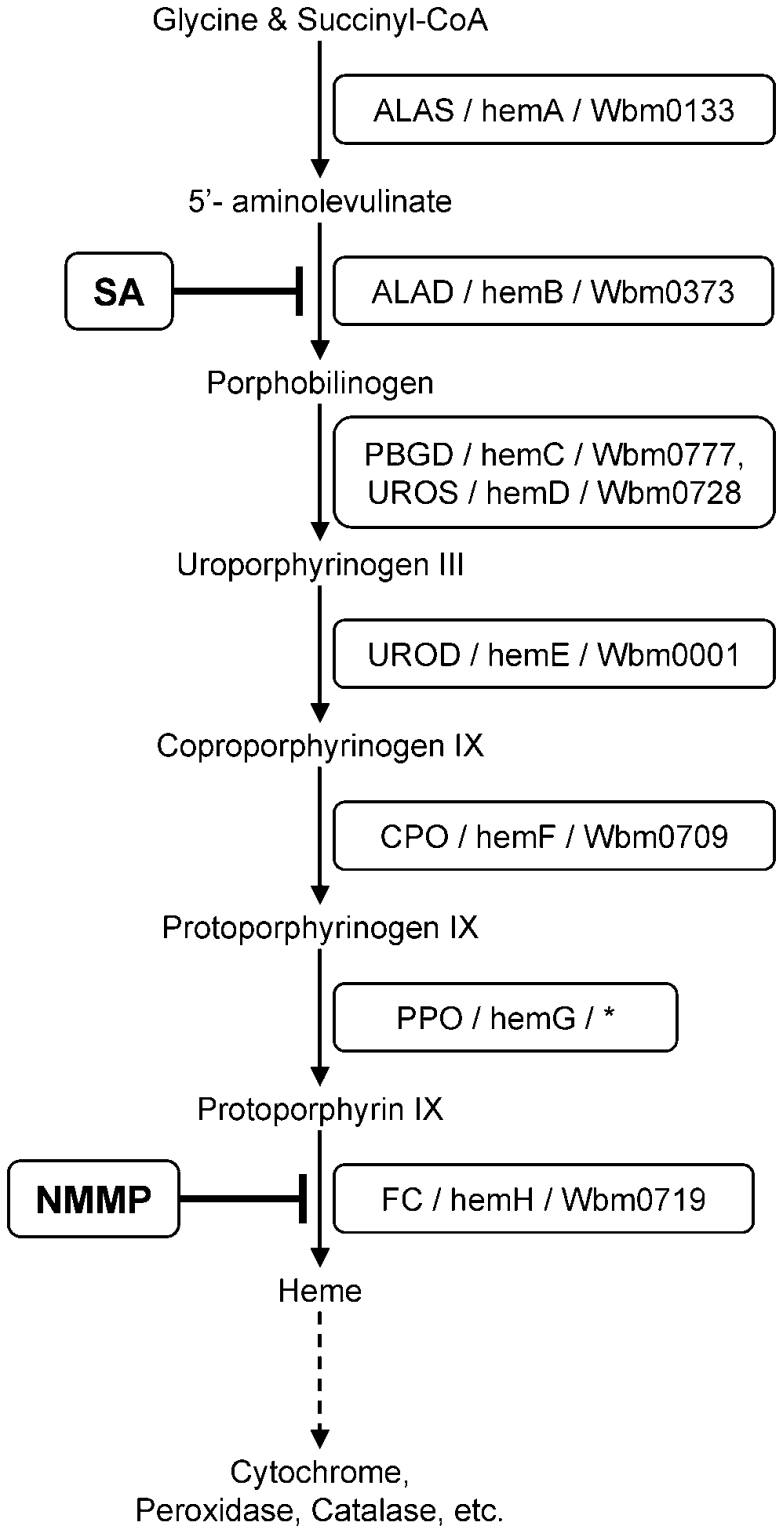
Schematic diagram of the heme biosynthetic pathway. The text in the boxes indicates the eukaryotic/prokaryotic/*Wolbachia* gene name. The asterisk indicates the gene missing in *Wolbachia* genome (PPO). ALAS, 5-aminolevulinate synthase (EC 2.3.1.37); ALAD, 5-aminolevulinate dehydratase (also known as PBGS, porphobilinogen synthase, EC 4.2.1.24); PBGD, porphobilinogen deaminase (EC 4.1.3.8); UROS, uroporphyrinogen-III synthase (EC 4.2.1.75); UROD, uroporphyrinogen-III decarboxylase (EC 4.1.1.37); CPO, coproporphyrinogen-IX oxidase (EC 1.11.1.10); PPO, protoporphyrinogen-IX oxidase (EC 1.3.3.4); FC, ferrochelatase (EC 4.99.1.1); SA, ALAD inhibitor Succinyl acetone; NMMP, FC inhibitor N-methyl mesoporphyrin.

Comparative analysis of available genomic sequences for *Wolbachia* (*w*Bm, GenBank accession no. AE017321) and its *B. malayi* nematode host (GenBank accession no. EF588824 to EF588901) provides insight into metabolic pathways that might contribute to the mutualistic symbiotic relationship [14]. This approach can be used to aid identification of potential anti-filarial drug targets. One biochemical pathway identified as potentially important in the symbiotic relationship between *w*Bm and its nematode host is heme biosynthesis. Heme, an iron-containing tetrapyrrole, is an essential cofactor for many proteins such as cytochromes, hemoglobins, peroxidases, and catalases, which are involved in a wide range of critical biological processes, including oxidative metabolism and electron transport. All but one of the C_4_-type heme biosynthetic genes are readily identified from the *w*Bm genome (Fig. 1). The only missing step, protoporphyrinogen-IX oxidase (PPO/hemG), has not been identified in many heme-producing bacteria [15]. However, all but one heme biosynthetic gene (*FC*/*hemH*, ferrochelatase, the last step in heme biosynthesis) is absent in the *B. malayi* genome [16], implying filarial nematodes are incapable of *de novo* heme biosynthesis, a condition that seems to be characteristic of all or most nematodes, including *Caenorhabditis elegans* [17]. Filarial worms presumably salvage heme/intermediates from their surroundings and/or acquire them from their *Wolbachia* endosymbionts. Heme deprivation may at least partially account for the effects caused by elimination of *w*Bm following antibiotic treatment of filarial worms. For example, it is already known that antibiotic treatment disrupts the L4 to L5 molt in *B. pahangi* [18]. Furthermore, heme-containing enzymes such as peroxidases have critical functions in the molting of *C. elegans* and orthologs exist in *B. malayi* [19],[20],[21]. In this report, we indicate that *Wolbachia* heme biosynthesis likely contributes to filarial worm survival and thus could be a potential anti-filarial drug target pathway.

## Materials and Methods

### Cloning, expression and purification of human and *Wolbachia* heme biosynthetic enzymes

Human heme gene cDNA clones were purchased from the Invitrogen human cDNA clone collection, except for the 5′-aminolevulinic acid synthetase cDNA clone which was purchased from Open Biosystems. *B. malayi* worms were purchased from TRS Labs, Athens, GA. *B. malayi* DNA (including *Wolbachia* DNA) was extracted using DNeasy extraction (Qiagen) according to the manufacturer's protocol. Based on available human, *Wolbachia* and *E. coli* sequences in the NCBI database, primers were designed with restriction endonuclease sites (Table S1) and used for full-length open reading frame (ORF) amplification by PCR with Phusion polymerase (New England Biolabs, NEB). After purification by QIAquick PCR purification (Qiagen) and digestion with corresponding restriction endonucleases (NEB), resulting PCR products were cloned into the pET21a+ vector (Novagen) for protein expression with a C-terminal 6XHis-tag. Correct clones were first identified by lysed-colony PCR and then verified by DNA sequencing. For improving protein expression and solubility, human 5′-aminolevulinic acid dehydratase (ALAD), *Wolbachia* porphobilinogen deaminase (PBGD) and *Wolbachia* ferrochelatase (FC) genes were codon-optimized by gene re-synthesis using DNAworks oligonucleotide designing software [22] and USER cloning methods [23].

All cloned heme genes were expressed in T7 Express competent *E. coli* (NEB), either with or without the RIL plasmid (Stratagene) which encodes *E. coli* rare tRNAs for arginine, isoleucine and leucine. Protein expression was induced with starting OD_600_ 0.3–0.4, 10–100 µM isopropyl β-D-thiogalactopyranoside (IPTG, Sigma), 18–48 hours at 14–16°C. The 6XHis-tagged proteins were purified under native conditions, using a nickel resin (Qiagen) according to a modified manufacturer's protocol. Buffers (100 mM Tris-HCl pH 8.0, 300 mM NaCl) containing different concentrations of imidazole (10–20 mM, 40–50 mM and 250 mM) were used as the lysis, wash and elution buffers, respectively. Purity of the proteins was verified on 4–20% SDS-PAGE gels (Invitrogen) and protein concentrations were measured on a Nanodrop ND-1000 (Thermo Scientific). Proteins were stored at −80°C in 10% glycerol for long-term storage or stored at 4°C for no more than 1 month for further analysis.

### Phylogenetic analysis

Homologous protein sequences were retrieved from the NCBI database via protein-protein BLAST similarity searches and were aligned using CLUSTAL ×1.83 [24]. The sequence alignments were further refined manually after the removal of large gaps and evolutionarily diverse regions. Based on protein sequence alignments, gene phylogenies for the *B. malayi Wolbachia* heme synthesis genes were derived from both Bayesian inference (BI) [25] and Maximum likelihood (ML) [26] methods. ML trees were constructed by the PROML programs of PHYLIP package version 3.65 [27] with global rearrangements and randomized input order options in conjunction with estimated parameter gamma and the proportion of invariable sites obtained from TREE-PUZZLE 5.1 [28] calculation, in which Quartet puzzling maximum likelihood (QP) analysis was carried out employing the JTT-f amino acid substitution probability model with a mixed eight category gamma+invariable-sites model of rate heterogeneity and 10,000 puzzling steps. ML analyses were performed by subsequent applications of SEQBOOT (100 replicates), PROML and CONSENSE. BI analyses were conducted with randomly produced starting trees, JTT amino acid substitution frequencies, four category gamma+invariable-sites model, 200,000 generations of searches. Posterior possibilities for the best trees were calculated using a 50% majority rule.

### *B. malayi* worm *ex vivo* motility assays

Fresh live adult male and female *B. malayi* worms were incubated with different concentrations of succinyl acetone (SA, Sigma) or methyl mesoporphyrin (NMMP, Frontier Scientific) (3 replicates/experiment, 1 adult female or 3 adult males/replicate, experiment repeated three times), which target ALAD and FC, respectively. Worms were cultured in RPMI-1640 with 2 mM glutamine, 25 mM HEPES (Gibco) with 10% Fetal Calf Serum (Gibco) and 100 U/ml streptomycin, 100 µg/ml penicillin, 0.25 µg/ml amphotericin B (Sigma). Medium was changed every 2 days. SA was freshly made in water at a concentration of 500 mM before use. Both NMMP and hemin (Frontier Scientific) were freshly prepared as 5 mM stock concentrations in 50% ethanol containing 0.02 N NaOH. In NMMP tests, control worms were cultured in medium containing 1% ethanol and 0.0004 N NaOH (“solvent only”) with and without 100 µM hemin. Motility was measured daily (similar to the method used by Rao et al [11]) as 0, no motility; 1, slight movement clearly observed under microscope; 2, minor movement readily observed by eye; 3, non-continual moderate movement; 4, continual moderate movement; 5, continual active movement.

### *C. elegans* growth assays

*C. elegans* Bristol N2 was cultured and maintained according to standard protocols [29]. Drug testing was performed as follows. Eggs were extracted from gravid hermaphrodites using alkaline hypochlorite treatment followed by extensive washes in M9 buffer (22 mM KH_2_PO_4_, 42 mM Na_2_HPO_4_, 86 mM NaCl, 1 mM MgSO_4_) [29]. Eggs were allowed to hatch overnight in S-basal buffer (0.1 M NaCl, 0.05 M KH_2_PO_4_ pH 6, 5 mg/ml cholesterol). The concentration of first-stage larvae (L1) was adjusted to obtain an average of 5 to 6 live animals per well. Pre-grown concentrated *E. coli* OP50 was added as a food source. The compounds to be tested were added at the appropriate concentration. Worms were cultured in 96-well plates (NUNC, Rochester, NY) in a 100 µl volume for three days at 20°C. Twenty-four wells were cultured for each compound at a given concentration. Only one generation was followed. The number of parental animals reaching adulthood was scored.

### Enzyme assays

Enzyme activities were assayed using purified recombinant C-terminal 6XHis-tagged *Wolbachia* and human ALAD proteins (wALAD & hALAD) at 37°C for 15–30 min. The enzyme reactions were carried out in 100 mM Bis-Tris Propane (BTP) buffer (Sigma, pH range 6.5–9.5) containing 1 µg protein, 5 mM substrate 5′-aminolevulinic acid (ALA) (Sigma) and 10 mM β-mercaptoethanol (Sigma) unless otherwise stated, in a total volume of 100 µl. All assays were initiated by the addition of ALA after enzyme pre-incubation for 20–30 min with various metal ions (e.g. Zn^2+^, Mg^2+^) and/or other reagents (e.g. the metal ion chelator EDTA, specific enzyme inhibitor SA). After determining optimal reaction pH, enzyme assays were further conducted in the presence of different concentrations of substrate ALA (for determination of *Km* and *Vmax*) or inhibitor SA (for calculation of EC_50_). The reaction was stopped by mixing with an equal volume of stop buffer (0.1 M HgCl_2_ in 12% Trichloroacetic acid) followed by the addition of 800 µl modified Ehrlich reagent for 10 min [30]. The product porphobilinogen (PBG) was subsequently estimated by measuring the absorbance at OD_555_. The molar extinction coefficient for PBG (60,200 M^−1^ cm^−1^) was used in calculation of PBG concentration (µmol PBG/mg of protein/h).

### Complementation assays in *E. coli*

*E. coli hemB* (*ALAD*) mutant strain RP523 [31] and *HemD* (Uroporphyrinogen III synthase, *UROS*) deletion mutation strain SASZ31 were obtained from the *E. coli* Genetic Stock Center (http://cgsc.biology.yale.edu/). *E. coli hemG* (*PPO*) deletion strain SASX38 [32] and *hemH* (*FC*) deletion strain VS200 [33] were generously provided by Dr. Harry A. Dailey, University of Georgia. The pET21a+ vectors carrying the corresponding human, *Wolbachia* and *E. coli* heme gene inserts were transformed into the above-mentioned *E. coli* mutant strains, both with and without RIL plasmid co-transformation alongside a vector only control, and with appropriate antibiotic selection. Transformants were selected on 20 µM hemin-containing LB plates with appropriate antibiotics and incubated at 37°C overnight. The selected transgenic clones were further tested on LB plates with no hemin addition.

### *E. coli*-based growth assays

*E. coli hemH* mutants containing human, *Wolbachia* or *E. coli* FC genes, were used in growth assays. The fresh transgenic *E. coli* mutants (grown to 0.6–0.8 OD_600_) were diluted to 0.01 OD_600_ before initiating growth assays in the presence or absence of different concentrations of the specific FC inhibitor, NMMP in LB medium. The *E. coli* cells were grown in a 30°C shaker (180 rpm) for 3 h before estimating the cell growth level by measuring the final OD_600_ values. The final cell density for the untreated controls varied from 0.4 to 1.0 OD_600_. Relative cell density was taken as a measure of toxicity. The average cell growth ratio (final OD_600_/0.01) for untreated control is set at 1.0.

## Results

### Phylogenetic analyses reveal significant evolutionary divergence between *Wolbachia* and human heme biosynthesis gene homologues

**Figure 2:**
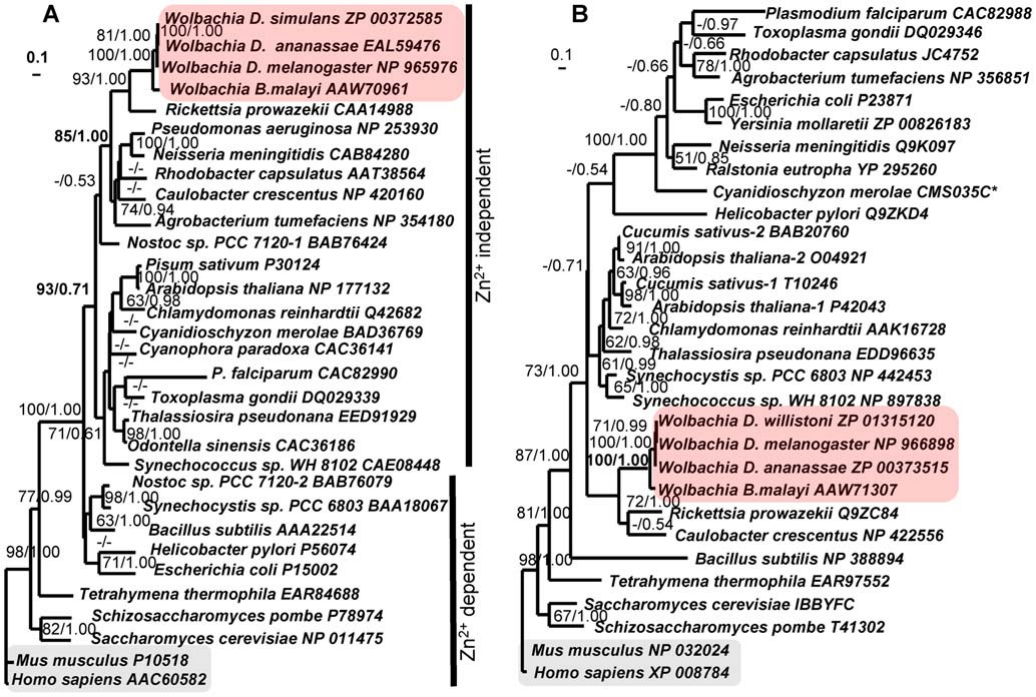
Gene phylogeny of ALAD and FC. A) ALAD, B) FC. The scaled Maximum likelihood (ML) consensus trees were inferred by ProML program of PHYLIP 3.65 package [27]. Two methods – Bayesian inference (BI) and ML analyses were used in gene phylogeny reconstruction and yielded similar tree topologies (details see Materials and Methods). The supporting values shown at nodes were obtained from ML and BI analyses, respectively and the values below 50% indicated by hyphens. The branch length scale shown below ML tree represents estimated substitutions per site. Available GenBank accession numbers follow the corresponding sequences. The asterisk indicates sequences retrieved from the organism's genome data; details are listed in the Text S1.

Based upon genomic DNA sequence analyses, both humans and *Wolbachia* share the C_4_-type heme biosynthetic pathway, usually consisting of eight components (Fig. 1) and phylogenies inferred by both ML and BI analyses indicate a deep evolutionary distance existing between homologues in this pathway. Sequences for all heme biosynthetic enzymes (except for the missing PPO gene) were obtained by database queries from diverse organisms (17–30 species, with exclusion of archaeal sequences due to their extreme divergence). They were aligned using ClustalX (with manual refinement). Conserved regions (157–312 sites) were used for phylogenetic reconstruction. The unrooted gene trees (except for ALAS), presented in Fig. 2 and Fig. S1, show that the gene homologues from both nematode and insect *Wolbachia* consistently group together and mostly within the *Rickettsia* subgroup of the alpha-proteobacterial cluster, sharing high amino acid sequence identities/similarities (70–87%/80–97%). By comparison, *B. malayi Wolbachia* heme synthesis genes only share 22–34% identities (29–53% similarities) with their human homologues. Among the seven identified components of *Wolbachia* heme pathway (Fig. 1), two were of particular interest (gene trees are presented in Fig. 2) owing to their significant divergence and biochemical properties. The ALAD gene tree shows that *Wolbachia* and human homologues belong to Zn^2+^-independent and Zn^2+^-dependent groups, respectively (Fig. 2A), which is confirmed by the absence of the critical cysteine residues in the Zn^2+^-binding sites in *Wolbachia* ALADs, while present in human ALAD (Text S1). Similarly, it is known that human FC contains both an N-terminal extension (encoding a mitochondrion-targeting signal) and a C-terminal extension (involved in formation of homodimers), and harbors an [Fe-S] cluster binding site (formed by 4 cysteine residues) [34],[35]. However, sequence analysis of *Wolbachia* FCs revealed that they do not have any of these features (Text S1). This is in line with the FC phylogenetic analysis that shows significant evolutionary divergence between human and *Wolbachia* FCs (Fig. 2B).

### *Wolbachia* heme biosynthesis might be crucial for *B. malayi* worm survival

**Figure 3:**
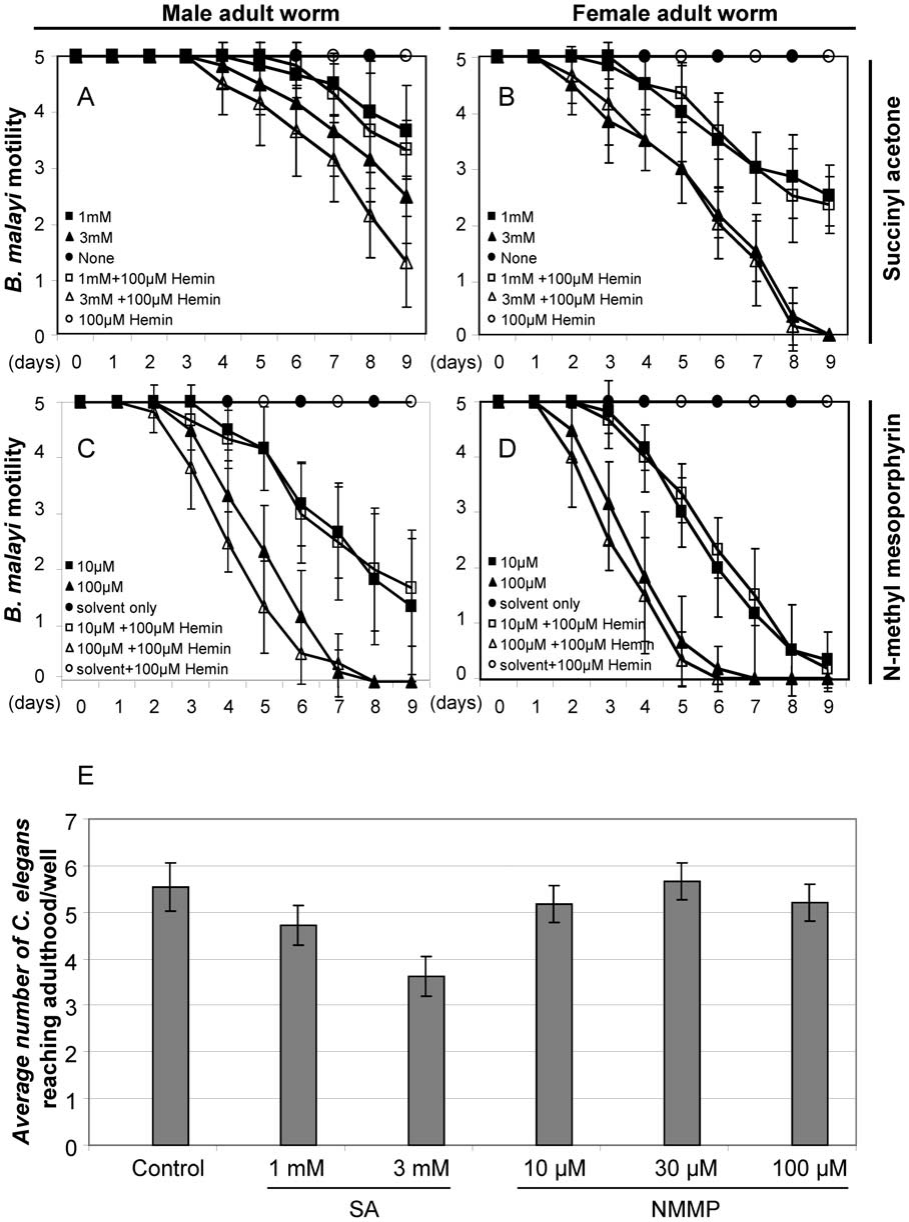
Effects of succinyl acetone (SA) and N-methyl-mesoporphyrin (NMMP) on the motility of *B. malayi* adult worms *ex vivo* and *C. elegans* larval growth. A) Effect of SA on *B. malayi* adult males, B) Effect of SA on *B. malayi* adult females, C) Effect of NMMP on *B. malayi* adult males, D) Effect of NMMP on *B. malayi* adult females, E) Effects of SA and NMMP on growth of *C. elegans*. The scale of viability (A–D) is arbitrarily measured by the relative motility of the adult worms: 0, no motility; 1, slight movement clearly observed under microscope; 2, minor movement readily observed by eye; 3, non-continual moderate movement; 4, continual moderate movement; 5, continual active movement. Control *B. malayi* worms (C, D) were cultured in medium containing 1% ethanol and 0.0004 N NaOH (“solvent only”) with and without 100 µM hemin. Data is compiled from 3 independent experiments (see Materials and Methods). Control *C. elegans* worms (E) were grown in 1% ethanol with 0.0004 N NaOH, which corresponds to the concentration of ethanol and NaOH present in the 100 µM NMMP test.

Two analog inhibitors, SA (1–3 mM) and NMMP (10–100 µM), were used in *B. malayi* worm *ex vivo* motility assays, which specifically target ALAD and FC, respectively. The results are shown in Fig. 3A–D. Motility was measured (similar to the method used by Rao et al [11]). Compared to the untreated controls, both SA and NMMP lead to significantly reduced motilities of adult worms during the nine-day treatments, independent of the addition of hemin to the medium (Fig. 3A–D). The tissue/cell structure of immotile worms (scaled as 0) seemed degenerative after treatment and no recovery was observed even after transferring these worms to fresh medium without inhibitor. Similar results were also observed in *B. malayi* mf larvae assays (data not shown). The effect of the inhibitors on female adult worms (Fig. 3B, D) appears more severe than that on male adult worms (Fig. 3A, C). The free-living nematode *C. elegans* was used as a worm control as it lacks the heme biosynthetic pathway and does not harbor an obligate endosymbiont, so has to salvage heme from the medium for viability [17]. In the presence of hemin, NMMP (10–100 µM) did not affect the growth of *C. elegans* larvae into adults (Fig. 3E) or worm fertility (data not shown) as expected. However, SA (1–3 mM) appeared to have a non-specific inhibitory effect on *C. elegans* larval development (Fig. 3E) and resulted in 100% sterility even at the lowest concentration tested (data not shown).

### Biochemical characterization of purified recombinant *Wolbachia* and human ALAD enzymes

**Figure 4:**
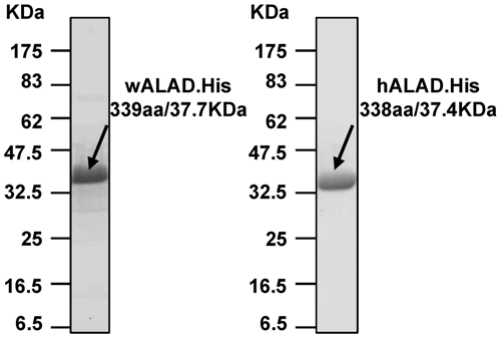
Purification profiles for C-terminally 6XHis-tagged wALAD and hALAD recombinant proteins.

cDNA clones in pET 21a+ of *Wolbachia* and human ALADs were transformed into *E. coli* containing the RIL plasmid and expressed as C-terminal 6XHis-tagged proteins. However, expression for hALAD was very poor, thus a codon-optimized version was made for improvement of expression. Examples of purified full-length C-terminal 6XHis-tagged wALAD (37.7 kDa) and hALAD (37.4 kDa) are presented in Fig. 4.

**Figure 5:**
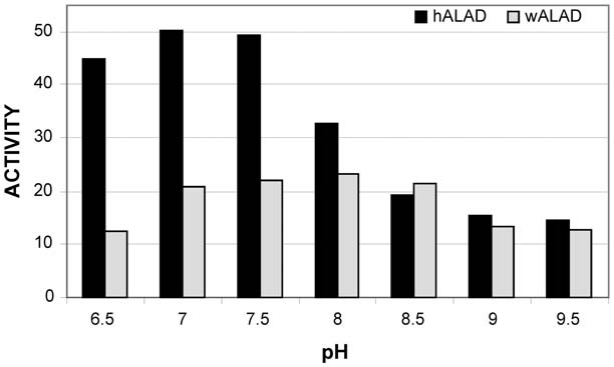
pH profiles for wALAD and hALAD enzyme activities. The enzyme activity is expressed as µmol PBG/mg of protein/h.

**Figure 6:**
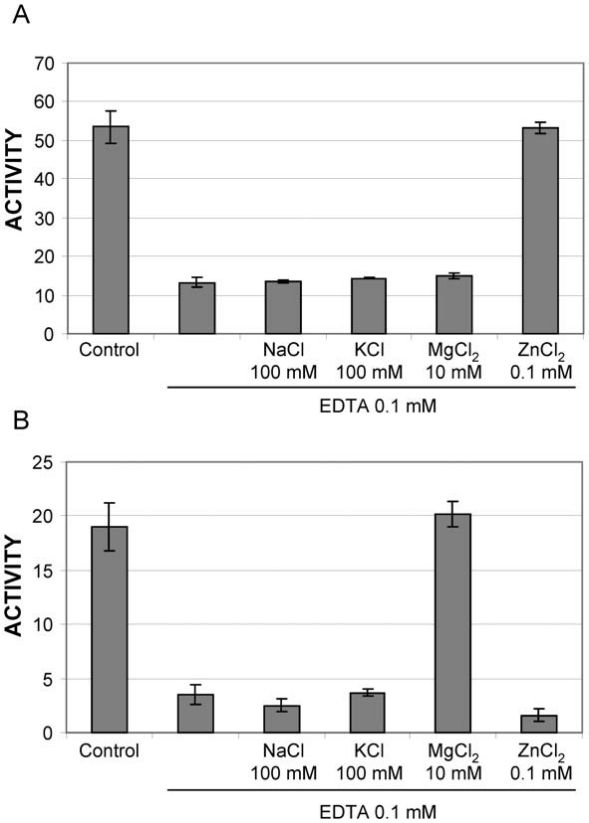
Effect of EDTA and metal ions on wALAD and hALAD enzyme activities. A) wALAD and B) hALAD were assayed at pH 8.0 and 7.0, respectively. Conventional concentrations of metal ions were used in this assay. The enzyme activity is expressed as µmol PBG/mg of protein/h. Control indicates sample without addition of extra metal ions or EDTA.

The pH profiles (Fig. 5) for hALAD and wALAD enzyme activities indicate an overlapped optimal pH range (pH 6.5–7.5 *vs* pH 7.0–8.5). hALAD is Zn^2+^-dependent. At optimal pH 7.0, its activity is inhibited by the metal ion chelator EDTA and recovered by addition of Zn^2+^ (Fig. 6A). wALAD activity (at its optimal pH 8.0) is also sensitive to EDTA inhibition, however, its activity is only restored by Mg^2+^ addition (Fig 6B). This suggests that wALAD is Zn^2+^-independent, which agrees with the absence of a putative Zn^2+^ binding site in wALAD, while it is present in hALAD. The maximum activity (*Vmax*) of hALAD (pH 7.0) is measured as 57.8±2.2 µmol porphobilinogen (PBG)/mg of protein/h and the *Km* value for substrate ALA is 0.35±0.06 mM (Fig. S2A), while for wALAD (pH 8.0), the *Vmax* and *Km* values are 22.5±1.1 µmol PBG/mg of protein/h and 0.32±0.07 mM, respectively (Fig. S2B). hALAD activity is about 2.5 times higher than that of wALAD with similar ALA substrate binding affinity.

**Figure 7:**
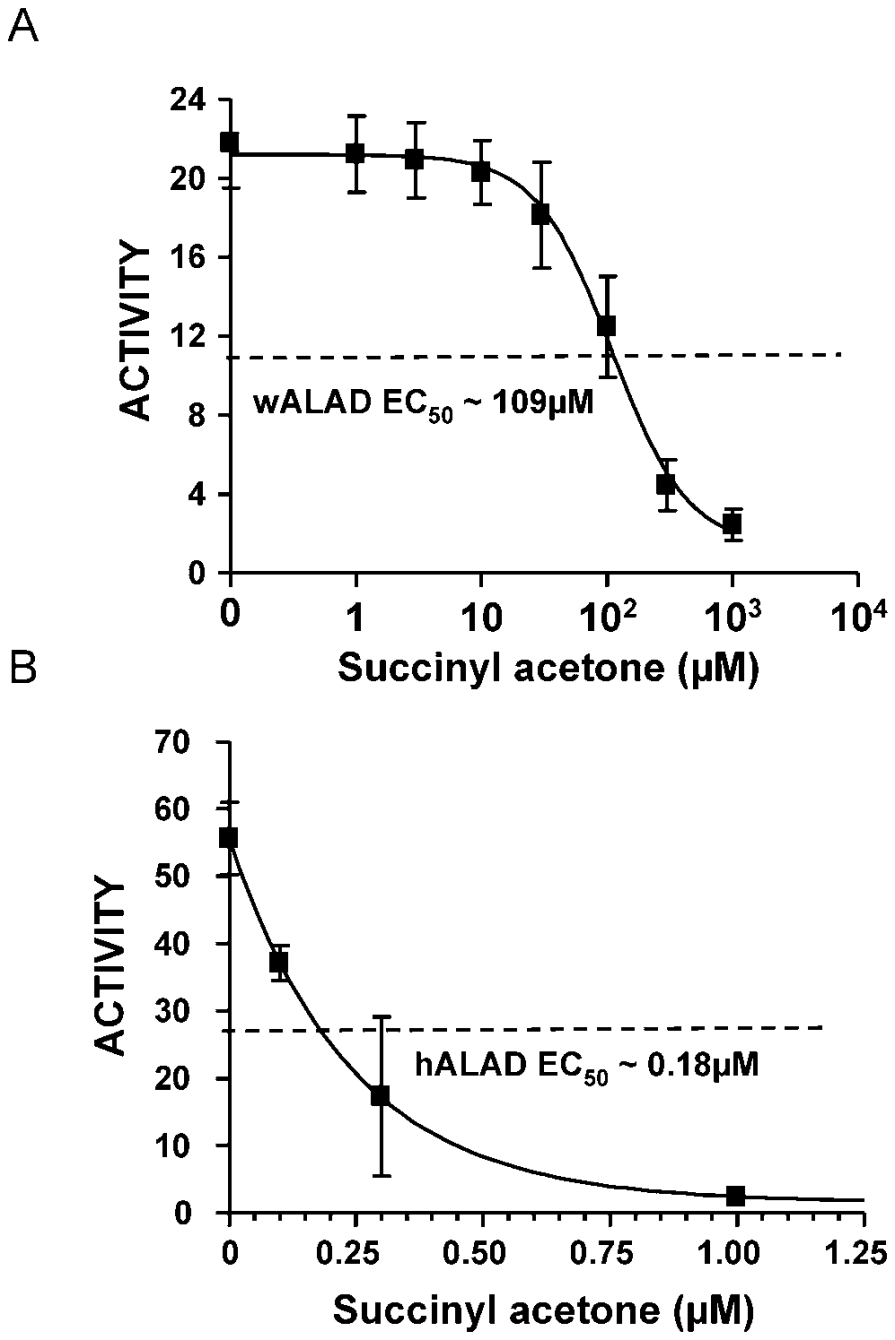
Biochemical characterization of recombinant wALAD and hALAD enzymes. A) Inhibition of wALAD enzyme activity by SA, B) Inhibition of hALAD enzyme activity by SA. The enzyme activity is expressed as µmol PBG/mg of protein/h.

SA is a specific ALAD inhibitor with different potency depending on the particular ALAD species involved. The sensitivities of wALAD and hALAD to SA are presented in Fig. 7. Both enzymes could be inhibited by SA, but with strikingly different inhibition profiles - EC_50_s for wALAD and hALAD were ∼109 µM and ∼0.18 µM, respectively, a 600 fold difference. This likely reflects a significant structural variation between these two enzymes.

### Functional complementation of *E. coli* heme deficient mutants and *E. coli*-based drug sensitivity assays

To test whether the cloned *Wolbachia* heme biosynthetic genes are functional, we performed complementation assays using *E. coli* heme deficient mutant strains (*hemB*, *hemD*, *hemG* and *hemH*). These assays complement the *in vitro* enzyme assays described above. The corresponding *Wolbachia* and human heme genes, cloned in the pET21a+ vector, were tested for activity, alongside a pET21a+ vector negative control.

An *E. coli hemB* mutant, transformed with the pET21a+ plasmid fails to grow on LB plates, unless hemin is added to the media. Transformations of the *E. coli hemB* mutant strain with wALAD or hALAD constructs result in strong colony growth on LB plates without hemin addition, similar to wild type *E. coli* growth, indicating functional expression of these two heme genes in *E. coli*. Similar complementation was observed for *E. coli hemD* and *hemH* mutants with *Wolbachia*/human UROS and *Wolbachia*/human FC genes, respectively. As mentioned previously, *Wolbachia* PPO is still un-recognized. It has been reported that overexpression of *E. coli* CPO might function as PPO [36] and therefore we tested an *E. coli hemG* mutant with the *w*Bm CPO construct. No complementation was observed, even under IPTG induction. As a positive control, an *E. coli hemG* mutant was functionally complemented by transformation with the human PPO construct. Our enzyme assays and *E. coli* complementation tests verified that *Wolbachia* ALAD, UROS and FC genes are functional. Since ALAD is the second step and FC is the last step of the heme biosynthesis pathways, our results indicate that *Wolbachia* has the ability to synthesize endogenous heme.

**Figure 8:**
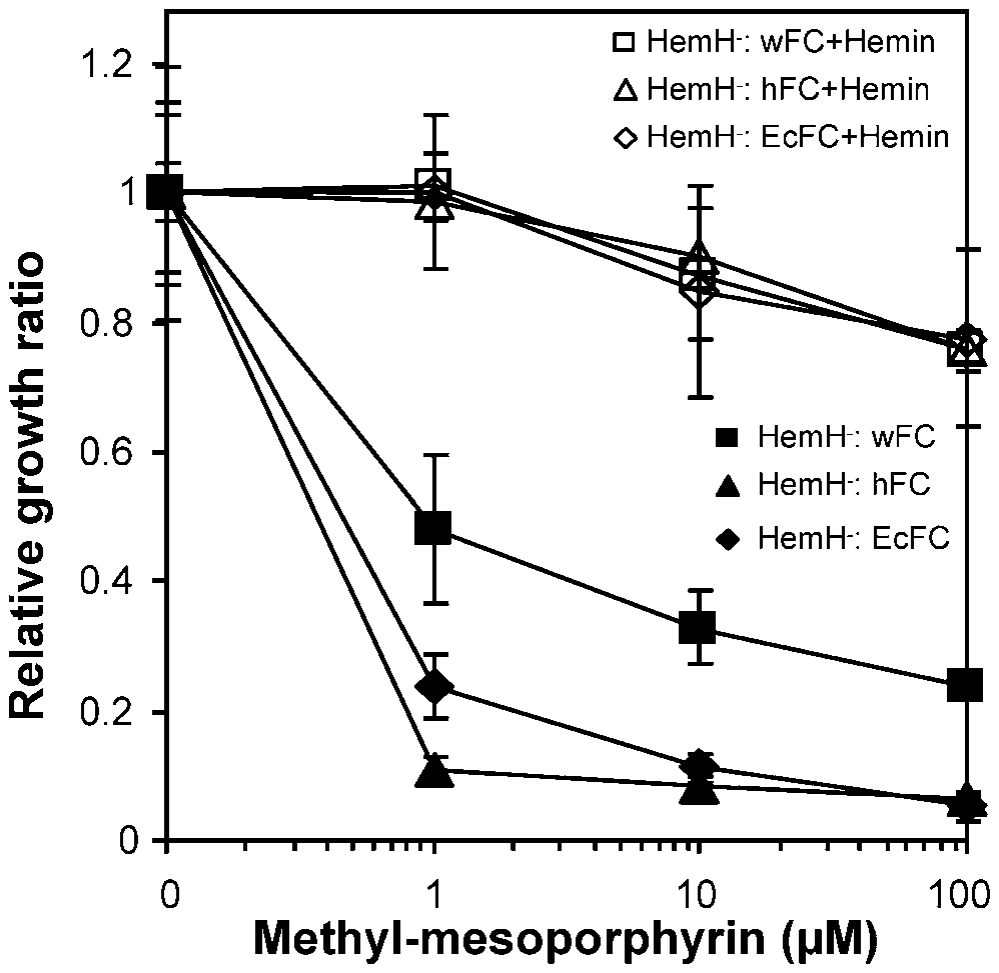
Growth inhibition assays by N-methyl mesoporphyrin (NMMP) of *E. coli HemH* mutants complemented with human, *Wolbachia* or *E. coli* FC genes. The average cell growth rate (final OD_600_/0.01) for untreated control is set as 1.0.

FC is a potential drug target based on its evolutionary divergence and differing protein features compared to its human homologue. Human FC (hFC) is an [Fe-S] protein, while *Wolbachia* FC (wFC) lacks [Fe-S] clusters. A potent FC inhibitor - NMMP was used in growth inhibition assays using *E. coli hemH* mutants transformed with wFC, hFC or *E. coli* FC (EcFC). The growth of all transformed strains was inhibited by NMMP with significantly different drug sensitivities (sensitivity level: hFC>EcFC>wFC) as compared to their non-treated controls (Fig. 8). The inhibition was readily overcome by the inclusion of hemin in the growth medium (Fig. 8). This assay further supports the possibility of using a rapid *E. coli*-based complementation assay to screen for specific inhibitors that will differentially target the *Wolbachia* heme synthesis enzymes.

## Discussion

Anti-*Wolbachia* chemotherapeutic treatment is an emerging approach for filariasis control [37]. Comparative genomic and bioinformatic analyses are shedding light onto the mutualistic symbiotic relationship between *Wolbachia* and its filarial host, revealing essential biochemical pathways in the bacterial endosymbiont that might provide critical metabolites for its worm host survival [14]. Based on our data from *ex vivo* worm assays, phylogenetic analyses, gene expression and purification profiles, *in vitro* enzyme assays, and *E. coli* complementation results, we have evaluated the possibility of the *Wolbachia* heme pathway as an anti-filarial drug target set. No heme biosynthetic genes except ferrochelatase (FC, the last step) have been identified from the worm host *B. malayi* genome sequence [16]. It appears that the nematode is not capable of synthesizing heme *de novo*, and thus may have to acquire heme from its *Wolbachia* endosymbiont or salvage environmental heme or both. The motilities of both *B. malayi* male and female worms are significantly reduced when exposed to the heme biosynthesis inhibitor SA (targeting ALAD), even in the presence of hemin in the medium (Fig. 3A–B). However, we believe that this effect was non-specific because a similar phenotype was observed when *C. elegans*, a heme auxotroph, which does not have the biosynthetic pathway at all, was exposed to SA (Fig. 3E), suggesting that SA may have some unspecific effect on *B. malayi*. In contrast, NMMP appears to be potent and specific in its inhibitory effect on the heme pathway, since it has an *in vivo* effect on *B. malayi* (Fig. 3C–D), but not on *C. elegans* (Fig. 3E). This inhibition can not be rescued by hemin (Fig. 3C–D), implying that *B. malayi* possibly lacks the capability of salvaging environmental heme, as has been demonstrated for *C. elegans* [38]. However, it should be noted that the *B. malayi* genome encodes for *FC* [16] and the effect of NMMP on *B. malayi* ferrochelatase (BmFC) could contribute to the inhibition of the worm viability (Wu et al, in preparation). We have no experimental evidence for a direct effect of NMMP on *Wolbachia*. Thus, the survival of *B. malayi* might be dependent on bacterial derived heme from the *Wolbachia* heme biosynthetic pathway and/or a functional BmFC which may utilize porphyrin intermediates from the endosymbiont or the environment. Given the observed differences between human and *B. malayi* FC proteins, specific inhibition of nematode FC in infected humans could be a potential drug target (Wu et al, in preparation).

Because mammals also synthesize heme via the C_4_-type pathway like *Wolbachia*, caution is needed when considering *Wolbachia* heme biosynthetic enzymes as anti-filarial drug targets. However, phylogenetic analyses (Fig. 2 & Fig. S1) revealed that significant evolutionary distances exist among the human and *Wolbachia* heme genes (except for ALAS), as shown by their low sequence similarities/identities (22–34%/29–53%). We corroborate these *in silico* studies with biochemical/pharmacological assays by cloning the *Wolbachia* and human genes and expressing the proteins. Difficulty in protein expression or purification of soluble proteins for hALAD, wPBGD & wFC was addressed by codon optimization. This helped improve expression levels, but still failed to yield soluble proteins, with the exception of synthetic hALAD.

Based on the significant difference (∼600 fold) in drug sensitivities between wALAD and hALAD enzymes (Fig. 7), large amounts of recombinant wALAD and hALAD proteins are currently being prepared for high throughput screening as part of anti-*Wolbachia* (A-WOL) drug discovery and development program (http://www.a-wol.com). Crystal structures are available for ALAD from human, mouse, yeast, *Pseudomonas aeruginosa* and *Chlorobium vibrioforme* (including a structure for yeast ALAD in complex with SA) from Protein Data Bank (PDB, http://www.rcsb.org). With this information, structural studies of *Wolbachia* and human ALAD by homology modeling may help explain the observed differences in SA sensitivity and help optimize identification of lead compounds obtained from the on-going drug screening effort.

Functional *Wolbachia* heme synthesis activity for several genes (ALAD, UROS and FC) along with their human homologues was demonstrated by complementation tests using the corresponding *E. coli* heme deficient mutants (*hemB*, *hemD* and *hemH*). As mentioned above, PPO, the penultimate step in heme pathway, is missing in *Wolbachia* and is unidentifiable from many other bacterial genomes, e.g. *Rickettsia* [15]. The *E. coli hemG* mutant was readily complemented by the human PPO gene; however, unlike *E. coli* CPO, *Wolbachia* CPO was incapable of rescuing PPO deficiency. Since PPO function is required for heme biosynthesis, it is possible that an unidentified oxidase may function as PPO in *Wolbachia*.

We have attempted to express and purify recombinant wFC (codon-optimized) and hFC proteins, however the yield of pure soluble protein was limited due to the formation of inclusion bodies. The availability of an *E. coli hemH* deficient mutant and functional complementation by wFC and hFC genes permit an alternative *E. coli*-based inhibition assay and drug-screening strategy. FC, the final step in heme biosynthesis, is responsible for insertion of iron into protoporphyin IX (PPIX) to form heme. NMMP is a strong PPIX analog, and competitively binds to the FC active site with *Ki* values in the nM range [39]. It was reported previously that FC enzymes could have dramatically different sensitivities to NMMP inhibition (>1000 fold, e.g. Human FC vs chicken FC) due to the differences existing in their active sites [40]. The *E. coli hemH* mutant, complemented with wFC, hFC and EcFC, is not sensitive to NMMP when grown in the presence of hemin. Significantly different sensitivities to NMMP were detected between human and *Wolbachia* FCs in absence of hemin, with human FC being much more sensitive. This may be accounted for by the structural difference in their active sites. Cell-based drug screening may help identify compounds more specifically targeting wFC instead of hFC. FC crystal structures from several species (human, yeast and *Bacillus*) are also available in PDB with a crystal structure of *Bacillus subtilis* FC complexed with NMMP [41]. *In silico* comparative studies based on molecular modeling can be conducted for *Wolbachia* and human FCs. *E. coli* mutants complemented by other *Wolbachia*/human heme genes (ALAD, UROS) will be used as potential screens as well for identification of compounds specifically inhibiting selected *Wolbachia* heme biosynthesis enzymes.

Our data suggest that the *Wolbachia* heme biosynthetic pathway is a potential anti-filarial drug target due to its requirement for survival of both *Wolbachia* and its filarial host. The presumptive transporters, responsible for heme trafficking, could be drug targets as well. However, it still remains unknown how the heme/heme intermediates might transfer from *Wolbachia* to its filarial host. No enzymes involved in traditional heme catabolism (e.g. heme oxygenase) have been identified from the *B. malayi* genome sequence. It is still an open question how transport, degradation, and regulation of heme occur in filarial parasites.

## Supporting Information

Figure S1 Gene phylogeny of ALAS, PBGD, UROS, UROD and CPO. A) ALAS, B) PBGD, C) UROS, D) UROD, E) CPO. The scaled Maximum likelihood (ML) consensus trees were inferred by ProML program of PHYLIP 3.65 package [27]. Two methods - Bayesian inference (BI) and ML analyses were used in gene phylogeny reconstruction and yielded similar tree topologies (details see Materials and Methods). The supporting values shown at nodes were obtained from ML and BI analyses, respectively and the values below 50% were indicated by hyphens. The branch length scale shown below the ML tree represents estimated substitutions per site. Available GenBank accession numbers follow the corresponding sequences. * Sequences were retrieved from the organism's genome data directly; details are listed in the supplementary sequence alignment file.
(2.06 MB TIF)

Figure S2 The *Vmax* and *Km* of the purified recombinant wALAD and hALAD enzymes. A) wALAD and B) hALAD were assayed at pH 8.0 and 7.0, respectively. The enzyme activity is expressed as µmol PBG/mg of protein/h.
(0.13 MB TIF)

Table S1 *Wolbachia*, human and *E. coli* heme gene specific primers were used for acquiring the full-length coding sequence by polymerase chain reaction (PCR) amplification, and were subsequently cloned into pET21a+ vector. Primers were designed according to information acquired from available *B. malayi*, *Wolbachia* (*w*Bm), human and *E. coli* genome databases. Restriction enzyme sites in primers are underlined. Abbreviations used: w: *Wolbachia*, h: human, Ec: *E. coli*, f: forward primer, r: reverse primer. The full names of the abbreviations for the heme biosynthetic enzymes are listed in the caption of Figure 1.
(1.77 MB TIF)

Text S1 Multiple sequence alignment for heme biosynthetic genes ALAS, ALAD, PBGD, UROS, UROD, CPO and FC.
(0.46 MB DOC)
