# Supplementary figures and images for: The Heme Biosynthetic Pathway of the Obligate Wolbachia Endosymbiont of Brugia malayi as a Potential Anti-filarial Drug Target
Source: PLoS Negl Trop Dis. 2009 Jul 14;3(7):e475. doi: 10.1371/journal.pntd.0000475 (PMC2703803; doi:10.1371/journal.pntd.0000475)

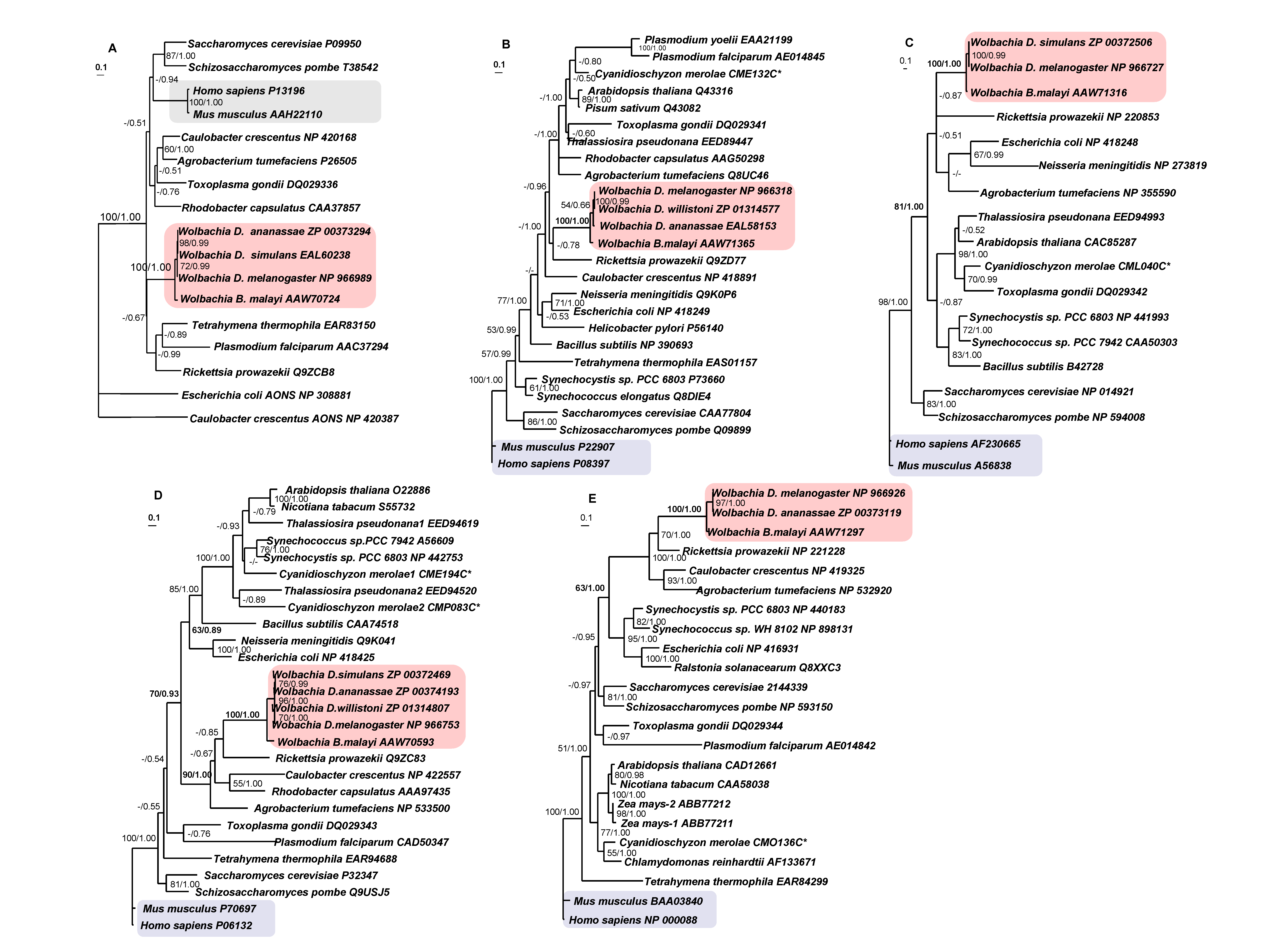

Supplement: Figure S1 — Gene phylogeny of ALAS, PBGD, UROS, UROD and CPO. A) ALAS, B) PBGD, C) UROS, D) UROD, E) CPO. The scaled Maximum likelihood (ML) consensus trees were inferred by ProML program of PHYLIP 3.65 package [27]. Two methods - Bayesian inference (BI) and ML analyses were used in gene phylogeny reconstruction and yielded similar tree topologies (details see Materials and Methods). The supporting values shown at nodes were obtained from ML and BI analyses, respectively and the values below 50% were indicated by hyphens. The branch length scale shown below the ML tree represents estimated substitutions per site. Available GenBank accession numbers follow the corresponding sequences. * Sequences were retrieved from the organism's genome data directly; details are listed in the supplementary sequence alignment file. (2.06 MB TIF) [file pntd.0000475.s001.tif]

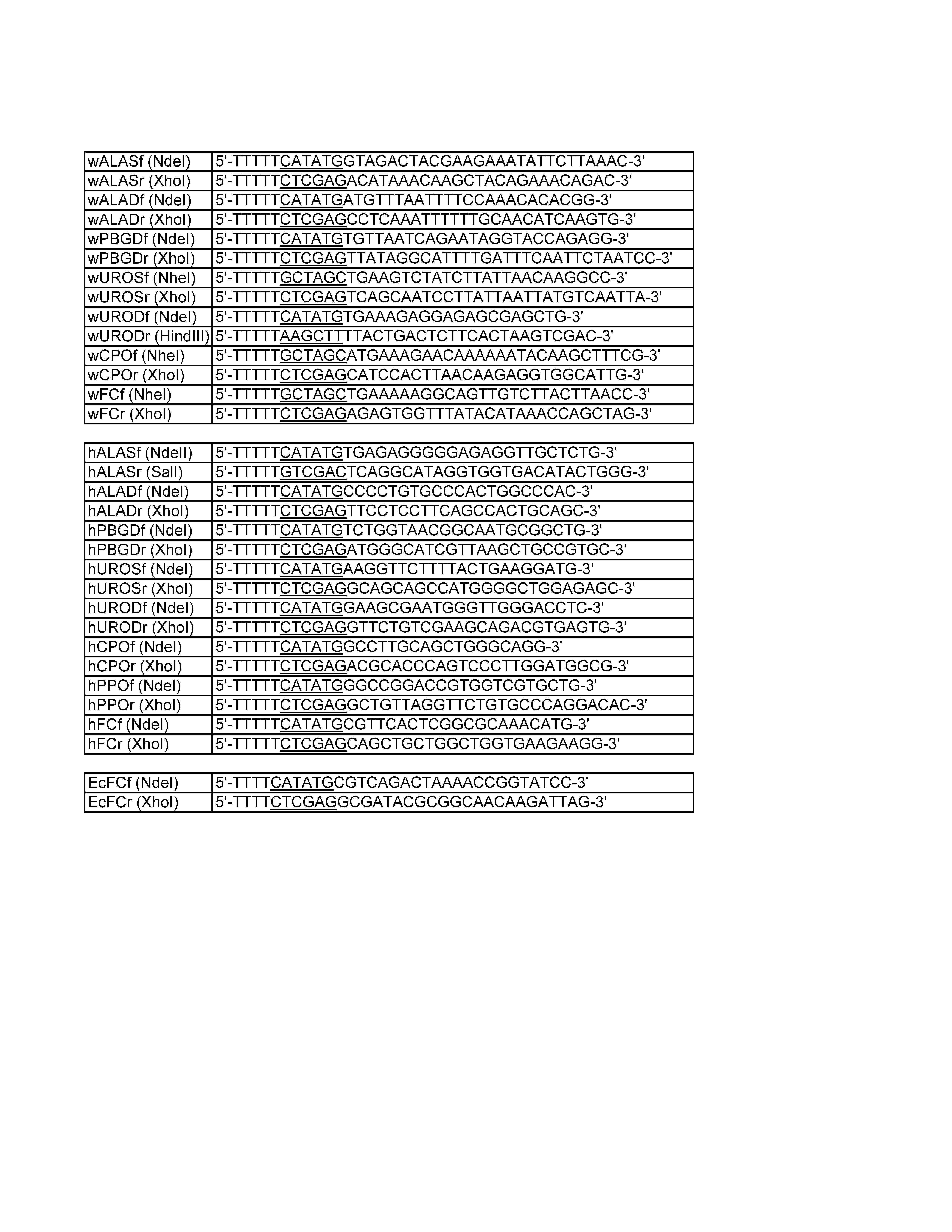

Supplement: Table S1 — Wolbachia, human and E. coli heme gene specific primers were used for acquiring the full-length coding sequence by polymerase chain reaction (PCR) amplification, and were subsequently cloned into pET21a+ vector. Primers were designed according to information acquired from available B. malayi, Wolbachia (wBm), human and E. coli genome databases. Restriction enzyme sites in primers are underlined. Abbreviations used: w: Wolbachia, h: human, Ec: E. coli, f: forward primer, r: reverse primer. The full names of the abbreviations for the heme biosynthetic enzymes are listed in the caption of Figure 1. (1.77 MB TIF) [file pntd.0000475.s003.tif]
