# Supplementary material for: The Heme Biosynthetic Pathway of the Obligate Wolbachia Endosymbiont of Brugia malayi as a Potential Anti-filarial Drug Target
Source: PLoS Negl Trop Dis. 2009 Jul 14;3(7):e475. doi: 10.1371/journal.pntd.0000475 (PMC2703803; doi:10.1371/journal.pntd.0000475)
Supplement: Text S1 — Multiple sequence alignment for heme biosynthetic genes ALAS, ALAD, PBGD, UROS, UROD, CPO and FC. (0.46 MB DOC) [file pntd.0000475.s004.doc]

**Supplementary sequence alignment file:**

**CLUSTAL X (1.83) multiple sequence alignment for ALAS (1)**

**17 taxa/312 characters from underlined evolutionarily conserved regions are used for phylogeny analysis; two proteobacterial AONS genes are used as outgroup**

**Legend for abbreviations used in the alignment:**

**SACCE** P09950 5-aminolevulinic acid synthase, mitochondrial precursor 548aa Saccharomyces cerevisiae Fungi

**SCHPO** T38542 5-aminolevulinate synthase mitochondrial precursor 525aa fission yeast Schizosaccharomyces pombe Fungi

**Human_H** P13196 5-aminolevulinic acid synthase, nonspecific, mitochondrial precursor/ALAS-H Housekeeping form Homo sapiens 640aa Animals

**Mouse_H** AAH22110.1 5-aminolevulinic acid synthase, nonspecific, mitochondrial precursor/ALAS-H Housekeeping form Mus musculus 641aa Animals

**CAUCR** NP_420168.1 408aa Caulobacter crescentus CB15 5-aminolevulinic acid synthase (alpha-proteobacteria)

**RHOCA** CAA37857.1 401aa Rhodobacter capsulatus 5-aminolevulinic acid synthase (alpha-proteobacteria)

**AGRTU** P26505 Agrobacterium tumefaciens 5-AMINOLEVULINIC ACID SYNTHASE (alpha-proteobacteria)

**RICPR** Q9ZCB8 414aa Rickettsia prowazekii 5-AMINOLEVULINIC ACID SYNTHASE (alpha-proteobacteria)

**Wolb** AAW70724 400aa Wolbachia endosymbiont strain TRS of Brugia malayi 5-AMINOLEVULINIC ACID SYNTHASE (alpha-proteobacteria)

**wAna** ZP_00373294 373aa Wolbachia endosymbiont of Drosophila ananassae 5-AMINOLEVULINIC ACID SYNTHASE (alpha-proteobacteria)

**wSim** EAL60238 369aa Wolbachia endosymbiont of Drosophila simulans 5-AMINOLEVULINIC ACID SYNTHASE (alpha-proteobacteria)

**wMel** NP_966989 402aa Wolbachia endosymbiont of Drosophila melanogaster 5-AMINOLEVULINIC ACID SYNTHASE (alpha-proteobacteria)

**Cliate** EAR83150 609aa delta-aminolevulinic acid synthetase Tetrahymena thermophila /gene#4597 retrieved from http://tigrblast.tigr.org/tgi/TIGR "Tetrahymena thermophila genome sequencing project" Ciliates

**EUGGR** AF325915_1 5-aminolevulinate synthase Missing N-terminus 446aa Euglena gracilis Euglenozoa

**TOXO** DQ029336 delta-aminolevulinic acid synthetase Toxoplasma gondii 584aa (apicomplexa)

**PLAFA** AAC37294.1 delta-aminolevulinic acid synthetase 630aa Plasmodium falciparum Apicomplexa

**AONS_Cau** NP_420387.1 8-amino-7-oxononanoate synthase/AONS [Caulobacter crescentus CB15] 440aa involved in coenzyme metabolism, closely related to ALAS gene used as outgroup alpha-proteobacteria

**AONS_Esc** NP_308881.1 8-amino-7-oxononanoate synthase/AONS [Escherichia coli O157:H7] 384aa used as outgroup gamma-proteobacteria

SACCE ------------------------------------------------------------

SCHPO ------------------------------------------------------------

Human_H MESVVRRCPFLSRVPQAFLQKAGKSLLFYAQNCPKMMEVGAKPAPRALSTAAVHYQQIKE 60

Mouse_H METVVRRCPFLSRVPQAFLQKAGKSLLFYAQNCPKMMEVGAKPAPRTLSTSAVHCQQVKE 60

RHOCA ------------------------------------------------------------

CAUCR ------------------------------------------------------------

AGRTU ------------------------------------------------------------

Wolb ------------------------------------------------------------

wAna ------------------------------------------------------------

wSim ------------------------------------------------------------

wMel ------------------------------------------------------------

TOXO ------------------------------------------------------------

Cliate --------------------MKGLNKFIQANKQCPFLYNHFKIDVSRNQIASDFLKKFVN 40

RICPR ------------------------------------------------------------

PLAFA ------------------------------------------------MRKKRTLKVSIN 12

AONS_Esc ------------------------------------------------------------

AONS_Cau ------------------------------------------------------------

SACCE --------------------------------MQRSIFARFGNSSAAVSTLNRLSTTAAP 28

SCHPO -------MERVVKLAAKHCPFVSKADPSALRRMAGAGLIRAGARCPVVRHALPVAAATGA 53

Human_H TPPASEKDKTAKAKVQQTPDGS--QQSPDGTQLPSGHPLPATSQGTASKCPFLAAQMNQR 118

Mouse_H TPPANEKEKTAKAAVQQAPDESQMAQTPDGTQLPSGHPSPATSQGSGSKCPFLAAQLSQT 120

RHOCA ------------------------------------------------------------

CAUCR ------------------------------------------------------------

AGRTU ------------------------------------------------------------

Wolb ------------------------------------------------------------

wAna ------------------------------------------------------------

wSim ------------------------------------------------------------

wMel ------------------------------------------------------------

TOXO ------------------------------------------------------------

Cliate LCPHLKNANKMNEREASGQTVSACASTNTTTHVAAAASFFSKCPINHSKLLGKKEKCTGC 100

RICPR ------------------------------------------------------------

PLAFA EIKKYCPFVKNIQFLYNTNEKKNNLVLSVMSDLCPVGKAINEKHFIIIDNKSKINIIKIL 72

AONS_Esc ------------------------------------------------------------

AONS_Cau ------------------------------------------------------------

SACCE HAKNGYATATG-------------AGAAAATA----TASSTHAAAAAAAAANHSTQ---- 67

SCHPO DVSRGFKSDSKQMAMEPSLDEIHLKAGVVNTG----SRTCRHADAVKAAAEAATTTPVTK 109

Human_H GSSVFCKASLELQEDVQEMNAVRKEVAETSAG----PSVVSVKTDGGDPSGLLKNFQDIM 174

Mouse_H GSSVFRKASLELQEDVQEMHAVRKEAAQSPVP----PSLVNVKTDGEDPSRLLKNFQDIM 176

RHOCA ------------------------------------------------------------

CAUCR ------------------------------------------------------------

AGRTU ------------------------------------------------------------

Wolb ------------------------------------------------------------

wAna ------------------------------------------------------------

wSim ------------------------------------------------------------

wMel ------------------------------------------------------------

TOXO -----------MRATTLNHQHFPPSPAPSAAA----GLESRGDALGSTDALSAAAPVASL 45

Cliate PHNACLHSNEAPLLNEITEKTLQESSQKCPFVGLLNIDNSTETAASKLLKNQEEYEQSVK 160

RICPR ------------------------------------------------------------

PLAFA KQANMQS-KVLVQCIKNKNIEKENMSNDDLLKSGKRNNNVLFYDILEKNKNDHSFQINDN 131

AONS_Esc ------------------------------------------------------------

AONS_Cau -------------------------MATTGSGGLAVFIWQYGRQTCGAPSVSQRGSDMSL 35

SACCE -------------------ESGFDYEGLIDSELQKKRLDKSYRYFNNINRLAKEFPLAHR 108

SCHPO KHQMPKHYASDLNGVGPATTPRFDYDTFYREELDKKHRDKSYRYFNNINRLAKEYPLAHL 169

Human_H QKQRPERVSHLLQDNLPKSVSTFQYDRFFEKKIDEKKNDHTYRVFKTVNRRAHIFPMADD 234

Mouse_H RKQRPERVSHLLQDNLPKSVSTFQYDHFFEKKIDEKKNDHTYRVFKTVNRRAQIFPMADD 236

RHOCA ----------------------MDYNLALDKAIQKLHDEGRYRTFIDIEREKGAFPKAQ- 37

CAUCR ----------------------MDYKAAFRSAVEQIREEGRYRVFADLKRQRGQFPRAT- 37

AGRTU ----------------------MDFEAFFTTELQSLHSEGRYRVFADIERQQGNFPRAT- 37

Wolb ---------------------MVDYEEIFLNKIKDIKEEGRYREFTHFASLPGRLPYIMD 39

wAna -------------------------------------MPGRLPHIMDYERNR-------- 15

wSim --------------------------------LPHIMDYERNR----------------- 11

wMel ---------------------MVNYEEIFLNKIKDIKDEGRYREFTHFASLPGRLPHIMD 39

TOXO LESKADLESSGINAEYAGALSPFFYEQRFTAAINELHAEGRYRVFAQLQRKRGAFPTAAI 105

Cliate KNFIMKNKSIQLDNQETSEKELNEYDQQFANAIKGLKTEGRYRVFNHIKKIAGRFPKALY 220

RICPR ---------------------MSYYDTIFNKHIDKIKSEGRYREFKSLKRQADNFPFAEY 39

PLAFA TIQKNNIIYKYINSLDEYKLFKNNCNNNLKDLLNKLYTDKRYRIFTILNKYRINYPNVYI 191

AONS_Esc ----------------------MSWQEKINAALDARRAADALRRRYPVAQGAGRWLVAD- 37

AONS_Cau SVCGLGAPCLSPGSILGKENDMRSLDAFAGQKLAALDAQSLRRRLSPTRRHDGAVVERD- 94

SACCE QREADK------------------------------------------------------ 114

SCHPO ADPNTR------------------------------------------------------ 175

Human_H YSDSLI------------------------------------------------------ 240

Mouse_H YTDSLI------------------------------------------------------ 242

RHOCA WNRPDG------------------------------------------------------ 43

CAUCR WTRQDG------------------------------------------------------ 43

AGRTU RYNANG------------------------------------------------------ 43

Wolb CERNRE------------------------------------------------------ 45

wAna ------------------------------------------------------------

wSim ------------------------------------------------------------

wMel YERNRE------------------------------------------------------ 45

TOXO FFDRQSGWEGDAESAGSGAQGAREAAAASPNAEGGTEIRREAAGAPGAHEEADRWMCQSV 165

Cliate TDSATN------------------------------------------------------ 226

RICPR ED---------------------------------------------------------- 41

PLAFA ENNKLMLPSFYEFYQKYGYKPCIGNIRYQLSASFEDN----------------NKNICSF 235

AONS_Esc ------------------------------------------------------------

AONS_Cau ------------------------------------------------------------

SACCE ------------------------VTVWCSNDYLALSKHPEVLDAMHKTIDKYGCGAGGT 150

SCHPO ------------------------VEVWCSNDYLNMGGHKKIREAMHQCIETYGGGAGGT 211

Human_H --------------------TKKQVSVWCSNDYLGMSRHPRVCGAVMDTLKQHGAGAGGT 280

Mouse_H --------------------TKKQVSVWCSNDYLGMSRHPRVCGAVMETVKQHGAGAGGT 282

RHOCA --------------------GKQDITVWCGNDYLGMGQHPVVLAAMHEALEAVGAGSGGT 83

CAUCR --------------------SEHEVVVWCSNDYLGQGQNPVVLEAMKAAVDEHGSGSGGT 83

AGRTU --------------------QRKDVTVWCSNDYLGMGQNPKVIEAMKAAIDHCGAGAGGT 83

Wolb ------------------------VIVWCSNNYLGMSQN----ESVVAAIQNSSVGAGGT 77

wAna -----------------------EVIVWCSNNYLGMSQN----ESVIAAIQNSSVGAGGT 48

wSim -----------------------EVIVWCSNNYLGMSQN----ESVIAAIQNSSVGAGGT 44

wMel ------------------------VIVWCSNNYLGMSQN----ESVIAAIQNSSVGAGGT 77

TOXO NEEASEAVASEAVAGIAKQLSQTEVQLWCSNDYLGMGQNPVVIQAAHEALDAAGAGAGGT 225

Cliate --------------------ETKEITVWCSNDYLGMGQHPTVRQAMIDAVKETGVGAGGT 266

RICPR ----------------------KQIVMWCINDYLGMSKHVKVMQASIDALLKYGVGSGGT 79

PLAFA SHKNKENYLFNFWNLHIDNVSNEKTVVWCSNDYLCLSNNEKIIEVGIETLKKIGNSSGGT 295

AONS_Esc ---------------------DRQYLNFSSNDYLGLSHHPQIIRAWKQGAEQFGVGSGGS 76

AONS_Cau ---------------------GKRMISFSCNDYLNLSQHHLVRAAAAEAALNYGAGAAAS 133

:. *:** . : . .:..:

SACCE RNIAGHNIPTLNLEAELATLHKKEGALVFSSCYVANDAVLSLLGQKMKDLVIFSDELNHA 210

SCHPO RNIAGHNQHAVRLEKSLADLHQKPAALVFGSCYVANDATLSTLGRKLPNCIFLSDEMNHA 271

Human_H RNISGTSKFHVDLERELADLHGKDAALLFSSCFVANDSTLFTLAKMMPGCEIYSDSGNHA 340

Mouse_H RNISGTSKFHVELEQALADLHGKDAALLFSSCFVANDSTLFTLAKMMPGCEIYSDSGNHA 342

RHOCA RNISGTTAYHRRLEAEIADLHGKEAALVFSSAYIANDATLSTLRLLFPGLIIYSDSLNHA 143

CAUCR RNISGTNHDHVLLEQELADLHGKEAGLLFTSGYVSNEATLSVVQKILPGLIIFSDELNHA 143

AGRTU RNISGTNHYHVLLEQELADLHGKESALIFTSGYVSNWATLGTLGQKIPGLIIFSDALNHA 143

Wolb RNISGTTKEVVELEQSLACLHNKEAALTFACGYLANQTTLSTLSSVIPDVVIFSDEKNHS 137

wAna RNISGTTKEVVELEKSLACLHKKEAALTFACGYLANQTTLSTLSSVIPGVVIFSDEKNHS 108

wSim RNISGTTKEVVELEKSLACLHKKEAALTFACGYLANQTTLSTLSSVIPGVVIFSDEKNHS 104

wMel RNISGTTKEVVELEKSLACLHKKEAALTFACGYLANQTTLSTLSSVIPGVVIFSDEKNHS 137

TOXO RNISGNCTFHLELERELAALHGKEAALLFTSGYVANEATLSTLGKLLPNLHIFSDEKNHA 285

Cliate RNIGGSSIYHTQLESELADLHFKEKAIVMSSGFVANQGAINALTKVLKDVIYLSDEKNHA 326

RICPR RNIGGNNISILELEKELADLHSKETALVFTSGFVANDTTLASLAKIIPDIVFFSDELNHA 139

PLAFA RNISGSLLNHTHLEYIIAKWYNKESSLLFTSGYIANVGALETLGKLLN-LIYISDEMNHA 354

AONS_Esc GHVSGYSVAHQALEEELAEWLGYSRALLFISGFAANQAVIAAMMAKED--RIVADRLSHA 134

AONS_Cau RLVTGDHPLLSDLEKRLAHLKGTEAACVFGSGYLANTGVIPTL--VGPGDVILIDALAHA 191

: * ** :* . : . : :* .: : * *:

SACCE SMIVGIKHANVKKHIFKHNDLNELEQLLQSYPKSVP-----KLIAFESVYSMAGSVADIE 265

SCHPO SMINGIRNSRCEKIIFKHNDLVDLEAKLASLPLNRP-----KIIAFESVYSMSGNVAPIS 326

Human_H SMIQGIRNSRVPKYIFRHNDVSHLRELLQRSDPSVP-----KIVAFETVHSMDGAVCPLE 395

Mouse_H SMIQGIRNSRVPKYIFRHNDVNHLRELLQRSDPSVP-----KIVAFETVHSMD-AVCPLE 396

RHOCA SMIEGIKRNAGPKRIFRHNDVAHLRELIAADDPAAP-----KLIAFESVYSMDGDFGPIK 198

CAUCR SMIAGIRNGGGPRKIFKHNDLAHLEQLLAEAPADAP-----KLIAFESVYSMDGDIADLA 198

AGRTU SMIEGIRYGRCERVIWKHNDLEDLEAKLKAADPNAP-----KLIAFESVYSMDGDIAPIK 198

Wolb SMIEGIKSGKRPKHIFKHNNVDHLEQLLKSVDRKTP-----KIIAFESIYSMDGDIAPLR 192

wAna SMIEGIKSGKRPKHIFKHNNVNHLEQLLKSIDKKTP-----KIIALESVYSMDGDIAPLE 163

wSim SMIEGIKSGKRPKHIFKHNNVNHLEQLLKSIDKKTP-----KIIALESVYSMDGDIAPLE 159

wMel SMIEGIKSGKRPKHIFKHNDVNHLEQLLKSIDKKTP-----KIIALESVYSMDGDVAPLK 192

TOXO SIIAGIRGARCAKRIFRHNDLIHLESLLAAAPADVP-----KLIVFESIYSMDGSVAPVK 340

Cliate SIIEGIRNSKADKVVWKHNDMEDLENKLKQLPLERN-----KIIIFESVYSMSGTISPIG 381

RICPR SIIAGIKSSRAEKYVYRHLDVQHLEKLLQSVDINKP-----KIIVFESAYSMDGFFSPIK 194

PLAFA SIINGIRESRCEKFIFKHNDMNDLERILYNLRINKQYENRKIMIVFESIYSMSGHISNIE 414

AONS_Esc SLLEAASLSPSQLRRFVHNDVTHLARLLASPCPGQQ------LVVTEGVFSMDGDSAPLA 188

AONS_Cau CIWAGAQLSGAKVVKFAHNDPADLERLLLAERGAAR----HALVATDGVFSMDGDIAPLD 247

.: . : * : .* : :: : .** :

SACCE KICDLADKYGALTFLDEVHAVGLYGPHGAGVAEHCDFESHRASGIATPKTNDKGGAKTVM 325

SCHPO EICDLAKKYGAITFLDEVHAVGMYGPRGAGVAEET---------------------PGLL 365

Human_H ELCDVAHEFGAITFVDEVHAVGLYGARGGGIGDRD----------------------GVM 433

Mouse_H ELCDVAHEFGAITFVDEVHAVGLYGARGGGIGDRD----------------------GVM 434

RHOCA EICDIADEFGALTYIDEVHAVGMYGPRGAGVAERD----------------------GLM 236

CAUCR GTVALAKKYGAMTYLDEVHAVGMYGPRGGGVAERD----------------------GLM 236

AGRTU EICDLADRYGAMTYLDEVHAVGMYGPRGGGIAERE----------------------GLM 236

Wolb EICNLADQYNAITYLDEVHAIGMYGLRGGGIAERE----------------------GLM 230

wAna AICDLADQHNAITYLDEVHAVGMYGSHGGGIAERE----------------------GLM 201

wSim AICDLADQHNAITYLDEVHAVGMYGSHGGGIAERE----------------------GLM 197

wMel EICDLADQHNAITYLDEVHAVGMYGSHGGGIAERE----------------------GLM 230

TOXO EICDLAEKFNSLTYIDEVHSVGMYGRTGGGVTEQS----------------------GQQ 378

Cliate EVCKLAKKYNALTFIDEVHAIGLYGKRGGGVAEMM----------------------GLM 419

RICPR DIINLAKKYNALTFIDEVHTVGLYGKQGGGISELL----------------------DCS 232

PLAFA YIVQLAKKYNALTYVDEVHAVGLYGNKGSGYLEEL----------------------HLC 452

AONS_Esc EIQQVTQQHNGWLMVDDAHGTGVIGEQGRGTCWLQ------------------------K 224

AONS_Cau ALSELCQRHDAWLLSDDAHGVGVLAEGRGSGALFP------------------------T 283

: ..... *:.* *: . .

SACCE DRVDMITGTLGKSFGSVGGYVAASRKLIDWFRSFAPGFIFTTTLPPSVMAGATAAIRYQR 385

SCHPO SRVDIITGTLAKSYGCVGGYIAASSTLVDMIRSLAPGFIFTTSLPPHVMVGALTAVEHLK 425

Human_H PKMDIISGTLGKAFGCVGGYIASTSSLIDTVRSYAAGFIFTTSLPPMLLAGALESVRILK 493

Mouse_H PKMDIISGTLGKAFGCVGGYIASTSLLIDTVRSYAAGFIFTTSLPPMLLAGALESVRILK 494

RHOCA HRIDIFNGTLAKAYGVFGGYIAASAKMVDAVRSYAPGFIFSTSLPPAIAAGAQASIAFLK 296

CAUCR GEIDIIEGTLGKAFGVMGGYITGDAEVIDAIRLMASGFIFTTSLPPALTAGALASVRWLK 296

AGRTU DRLTIIEGTLGKAFGVMGGYITGSTAVCDFIRSFASGFIFTTALPPSLAAGAIASIQHLK 296

Wolb DRVTVIQGTLSKAFGVMGGYIASSKSLVDVIRSSAPGFIFTTAMSPVLAAAAKASVEHLK 290

wAna DRITVIQGTLSKAFGVMGGYIASSKSLVDVIRSSAPGFIFTTAMSPVLAAAAKASVEHLK 261

wSim DRITVIQGTLSKAFGVMGGYIASSKSLVDVIRSSAPGFIFTTAMSPVLAAAAKASVEHLK 257

wMel DRITVIQGTLSKAFGVMGGYIASSKSLVDVIRSSAPGFIFTTAMSPVLAAAAKASVEHLK 290

TOXO LRVDLINGTLAKAVGVFGGYVAGMATLIDCIRSYAAGFIFTSSVPPAVAAAATASIRYLR 438

Cliate DQIDIFSGTLGKAYGCVGGYIAGNSLLIDCIRSFAQNFIFTTSIPPCIAQAAKTSIAYVK 479

RICPR NQIDIIQGTLAKAYGTIGGYITSNYNLIDAIRLTAPGFIFTTSLPPVISTAATHSIRHLK 292

PLAFA NHIDIINGTLSKAIGSLGGFICANKYYIDVIRSYSSHFIFTTSLTPVNINTSAEAIHIIQ 512

AONS_Esc VKPELLVVTFGKGFGVSGAAVLCSSTVADYLLQFARHLIYSTSMPPAQAQALRASLAVIR 284

AONS_Cau AKIPLQMGTLSKALGSYGGYLCGSQAVVDLLKTRARTLVYATGLPPASAAAALASLDLIA 343

. : *:.*. * *. : * . : ::::: :.* ::

SACCE CHI--DLRTSQQKHTMYVKKAFHELGIPVIPNPSHIVPVLIGNADLAKQASDILINKHQI 443

SCHPO VSN--VEREQQRSAVRRVKQSLSEIGIPVLSNDTHIVPAMVGDAHLAKLASDSLLHDHNI 483

Human_H SAEGRVLRRQHQRNVKLMRQMLMDAGLPVVHCPSHIIPVRVADAAKNTEVCDELMSRHNI 553

Mouse_H SSEGRALRRQHQRNVKLLRQMLMDAGLPVIHCPSHIIPVRVADAAKNTEICDELMTRHNI 554

RHOCA TAEGQKLRDAQQMHAKVLKMRLKALGMPIIDHGSHIVPVVIGDPVHTKAVSDMLLSDYGV 356

CAUCR QHP--EVREIHQERAATLKAMFKAAGLPVMDSVSHIVPVLVGDPVHCKMISDMLLADFGV 354

AGRTU ASP--FERARHQDRVRKLRGLLDARGIPHMDNPSHIVPVMVGDAAKCKWISDILLDSHGV 354

Wolb SSN--VEREKQRQVVEKVKNSLSSVGINFIPTETHIIPIIIGDPELSKRASKLLFDEYGV 348

wAna SSN--IEREKQKQVVEKVKNSLRNAGINFIPTETHIIPIIIGNSELSKKASKLLFDEYGI 319

wSim SSN--IEREKQKQVVEKVKNSLRNAGINFIPTETHIIPIIIGNSELSKKASKLLFDEYGI 315

wMel SSN--IEREKQKQVVEKVKSSLRNAGINFIPTETHIIPIIIGNSELSKKASKLLFDEYGI 348

TOXO RSS--TERYLQQLRATQLKTLLLSRDFPVLLNPSHIVPLLVGCPIACKRASDLLLHEHKL 496

Cliate EHN--ELRERLHFIARLIKKRCNDKNIPILPNESHIIPVFIGDAKKAKMASDLLMQKYDI 537

RICPR ESN--EERIKHQEVVTKLKNSFEHFNIPYLKNESHIIPIIIGDPIKATKVSNMLLNEYGI 350

PLAFA NDM--SLRKKLTQVVNKTKQKLQERGIQVLHNNSHIVVLMINSAEKCKQICDDLLKEYNI 570

AONS_Esc SDEGDARREKLAALITRFRAGVQDLPFTLADSCSAIQPLIVGDNSRALQLAEKLRQQ-GC 343

AONS_Cau ANP-------TMTEVPLAKARLFTRRLGLPEACSPIVPVVLGSAESALAASTELQNQ-GF 395

: : : * : . *

SACCE YVQAINFPTVARGTERLRITPTPGH-TNDLSDILINAVDDVFNELQLPRVRDWESQGGLL 502

SCHPO YVQSINFPTVSVGTERLRITPTPAHNTEHYVQSLTNAMNDVWSKFNINRIDGWEKRG--- 540

Human_H YVQAINYPTVPRGEELLRIAPTPHH-TPQMMNYFLENLLVTWKQVGLELKPHSSAECNFC 612

Mouse_H YVQAINYPTVPRGEELLRIAPTPHH-TPQMMNFFVEKLLVTWKRVGLELKPHSSAECNFC 613

RHOCA YVQPINFPTVPRGTERLRFTPSPVH-DLKQIDGLVHAMDLLWARCA-------------- 401

CAUCR YVQPINYPTVPRGTERLRFTPTPFH-TDDMMRKLVAAMEKLWAHCNVARMGGYAA----- 408

AGRTU YVQPINYPTVPRKTERLRITPTPLH-SDADIEHLVGALHQLWSHCALARAVA-------- 405

Wolb YVQHINYPTVPRGTERFRITPTPYH-TDEMIEHLTESLIKVFVKLSVSVACLC------- 400

wAna YVQHINYPTVPRGTERFRITPTPYH-TDEMIEHLTESLVKVFEKLSISLACLCYS----- 373

wSim YVQHINYPTVPRGTERFRITPTPYH-TDEMIEHLTESLVKVFEKLSISLACLCYS----- 369

wMel YVQHINYPTVPRGTERFRITPTPYH-TDEMIEHLTESLVKVFEKLSISVACLCYS----- 402

TOXO YIQPINYPTVPRGSERLRVTPGPLH-SYDDVLNLVDALDQVFFGRLKCCSGAPAAPGPLL 555

Cliate YVQPINYPTVPKGQELLRISPTPNH-NEDMVEKLVLALEGVFEELQLRENFGQELSHNFT 596

RICPR YVQHINFPTVPRGTERLRIIPTPAH-TDKMINDLSTALVHIFDELDIELSSAKELNKEVR 409

PLAFA YIQPINYPTVPMGMERIRITPSPFH-TDEQIFKLVNSLYTLFKKYQVNMFDKKNKHTLMK 629

AONS_Esc WVTAIRPPTVPSGTARLRLTLTAAH-EMQDIDRLLEVLHGNG------------------ 384

AONS_Cau LVVAIRPPTVPDGTARLRIAFSAAH-EDADIIRLADAIAKLRETAS-------------- 440

: *. ***. :*. . * : :

SACCE GVGESGFVEESNLWTSSQLSLTNDDLNPNVRDPIVKQLEVSSGIKQ 548

SCHPO -------IDVGRLCKFPVLPFTTTH--------------------- 558

Human_H RRPLHFEVMSEREKSYFSGLSKLVSAQA------------------ 640

Mouse_H RRPLHFEVMSEREKAYFSGMSKMVSAQA------------------ 641

RHOCA ----------------------------------------------

CAUCR ----------------------------------------------

AGRTU ----------------------------------------------

Wolb ----------------------------------------------

wAna ----------------------------------------------

wSim ----------------------------------------------

wMel ----------------------------------------------

TOXO QQSRVSRVRRTVDLFRIFFSGRSLRLSSQ----------------- 584

Cliate EPLSLAEKLIITA--------------------------------- 609

RICPR LHLIA----------------------------------------- 414

PLAFA L--------------------------------------------- 630

AONS_Esc ----------------------------------------------

AONS_Cau ----------------------------------------------

**CLUSTAL X (1.83) multiple sequence alignment for ALAD (2)**

**29 taxa/280 characters from underlined conserved regions are used for phylogeny analysis**

**Note: Cysteine residues involved in Zn^2+^-binding are labeled in red color.**

**Legend for abbreviations used in the alignment:**

**ARAT** 430aa NP_177132 delta-aminolevulinic acid dehydratase, Arabidopsis thaliana Plants

**PEA** 398aa P30124 Delta-aminolevulinic acid dehydratase, Pisum sativum Plants

**CHLRE** 390aa Q42682 Delta-aminolevulinic acid dehydratase, Chlamydomonas reinhardtii Green Algae

**CYAME** 402aa BAD36769 delta-aminolevulinic acid dehydratase Cyanidioschyzon merolae Red Algae

**ODOSI** 412aa CAC36186 Delta-aminolevulinic acid dehydratase Odontella sinensis Diatoms/Heterokonts

**THAPS** EED91929 367aa grail.38.12.1 retrieved from http://genome.jgi-psf.org/thaps1/thaps1.home.html thalassiosira pseudonana Diatoms/Heterokonts

**CYAPA** 419aa CAC36141 ALA dehydratase Cyanophora paradoxa Glaucophytes

**TOXO** 693 aa DQ029339 delta-aminolevulinic acid dehydratase Toxoplasma gondii Apicomplexa

**PLAF** 451aa CAC82990 delta-aminolevulinic acid dehydratase Plasmodium falciparum Apicomplexa

**RICP** 330aa CAA14988 DELTA-AMINOLEVULINIC ACID DEHYDRATASE Rickettsia prowazekii alpha-proteobacteria

**CAUL** 334aa NP_420160 delta-aminolevulinic acid dehydratase Caulobacter crescentus CB15 alpha-proteobacteria

**AGRO** 360aa NP_354180 delta-aminolevulinic acid dehydratase Agrobacterium tumefaciens str C58 alpha-proteobacteria

**RHOD** 332aa AAT38564 delta-aminolevulinic acid dehydratase Rhodobacter capsulatus alpha-proteobacteria

**Wolb** AAW70961 331aa Wolbachia endosymbiont strain TRS of Brugia malayi delta-aminolevulinic acid dehydratase alpha-proteobacteria

**wAna** EAL59476 331aa Wolbachia endosymbiont of Drosophila ananassae delta-aminolevulinic acid dehydratase alpha-proteobacteria

**wSim** ZP_00372585 331aa Wolbachia endosymbiont of Drosophila simulans

delta-aminolevulinic acid dehydratase alpha-proteobacteria

**wMel** NP_965976 331aa Wolbachia endosymbiont of Drosophila melanogaster

delta-aminolevulinic acid dehydratase alpha-proteobacteria

**NEIME** 338aa CAB84280 delta-aminolevulinic acid dehydratase Neisseria meningitidis Z2491 beta-proteobacteria

**ECOLI** 334aa P15002 Delta-aminolevulinic acid dehydratase Escherichia coli Zn+ gamma-proteobacteria

**HELPY** 323aa P56074 Delta-aminolevulinic acid dehydratase Helicobacter pylori Epsilon-proteobacteria

**PSEAE** 334aa NP_253930 delta-aminolevulinic acid dehydratase Pseudomonas aeruginosa PAO1 gamma-proteobacteria

**Ciliate** EAR84688 336aa delta-aminolevulinic acid dehydratase retrieved from http://tigrblast.tigr.org/tgi/TIGR "Tetrahymena thermophila genome sequencing project" Tetrahymena thermophila Ciliate

**HOMO** 330aa AAC60582 delta-aminolevulinate dehydratase Homo sapiens Animals

**MOUSE** 330aa P10518 Delta-aminolevulinic acid dehydratase Mus musculus Animals

**SACC** 342aa NP_011475 Delta-aminolevulinate dehydratase Saccharomyces cerevisiae Fungi

**SCHP** 329aa P78974 Delta-aminolevulinate dehydratase Schizosaccharomyces pombe Fungi

**NOSTOC1** 336aa BAB76424 delta-aminolevulinic acid dehydratase Nostoc sp. PCC 7120 Cyanobacteria

**NOSTOC2** 326aa BAB76079 delta-aminolevulinic acid dehydratase Nostoc sp. PCC 7120 Cyanobacteria

**SYNOWH** 333aa CAE08448 delta-aminolevulinic acid dehydratase Synechococcus sp. WH 8102 Cyanobacteria

**SYNE** 327aa BAA18067 delta-aminolevulinic acid dehydratase Synechocystis sp. PCC 6803 Cyanobacteria

**BACS** 324aa AAA22514 delta-aminolevulinic acid dehydratase Bacillus subtilis firmicutes G+ bacteria

ARAT ------------------------------------------------------------

PEA ------------------------------------------------------------

SYNOWH ------------------------------------------------------------

CHLRE ------------------------------------------------------------

CYAPA ------------------------------------------------------------

ODOSI ------------------------------------------------------------

THAPS ------------------------------------------------------------

CYAME ------------------------------------------------------------

TOXO MSFFLLFFLPPQEFRVHVCPPSGPVCLSDHFSFSKMRPLSLFFRLSFLLFVLLFPSPSVD 60

Wolb ------------------------------------------------------------

wMel ------------------------------------------------------------

wAna ------------------------------------------------------------

wSim ------------------------------------------------------------

CAUL ------------------------------------------------------------

AGRO ------------------------------------------------------------

RHOD ------------------------------------------------------------

NEIME ------------------------------------------------------------

PSEAE ------------------------------------------------------------

ECOLI ------------------------------------------------------------

HELPY ------------------------------------------------------------

NOSTOC2 ------------------------------------------------------------

SYNE ------------------------------------------------------------

BACS ------------------------------------------------------------

HOMO ------------------------------------------------------------

MOUSE ------------------------------------------------------------

SACC ------------------------------------------------------------

SCHP ------------------------------------------------------------

Ciliate ------------------------------------------------------------

NOSTOC1 ------------------------------------------------------------

RICP ------------------------------------------------------------

PLAF ------------------------------------------------------------

ARAT ------------------------------------------------------------

PEA ------------------------------------------------------------

SYNOWH ------------------------------------------------------------

CHLRE ------------------------------------------------------------

CYAPA ------------------------------------------------------------

ODOSI ------------------------------------------------------------

THAPS ------------------------------------------------------------

CYAME ------------------------------------------------------------

TOXO ALLQSSSVAGSFPTPASVASSGRSVSPHNALYVHSRTEATAGLPSGASLAITLNGESEEP 120

Wolb ------------------------------------------------------------

wMel ------------------------------------------------------------

wAna ------------------------------------------------------------

wSim ------------------------------------------------------------

CAUL ------------------------------------------------------------

AGRO ------------------------------------------------------------

RHOD ------------------------------------------------------------

NEIME ------------------------------------------------------------

PSEAE ------------------------------------------------------------

ECOLI ------------------------------------------------------------

HELPY ------------------------------------------------------------

NOSTOC2 ------------------------------------------------------------

SYNE ------------------------------------------------------------

BACS ------------------------------------------------------------

HOMO ------------------------------------------------------------

MOUSE ------------------------------------------------------------

SACC ------------------------------------------------------------

SCHP ------------------------------------------------------------

Ciliate ------------------------------------------------------------

NOSTOC1 ------------------------------------------------------------

RICP ------------------------------------------------------------

PLAF ------------------------------------------------------------

ARAT ------------------------------------------------------------

PEA ------------------------------------------------------------

SYNOWH ------------------------------------------------------------

CHLRE ------------------------------------------------------------

CYAPA ------------------------------------------------------------

ODOSI ------------------------------------------------------------

THAPS ------------------------------------------------------------

CYAME ------------------------------------------------------------

TOXO RRNGARRKGSKSLGFLVAPPLSRSCTTEQAGELFSGGAKVRGLNVQVTASPVGRELASSL 180

Wolb ------------------------------------------------------------

wMel ------------------------------------------------------------

wAna ------------------------------------------------------------

wSim ------------------------------------------------------------

CAUL ------------------------------------------------------------

AGRO ------------------------------------------------------------

RHOD ------------------------------------------------------------

NEIME ------------------------------------------------------------

PSEAE ------------------------------------------------------------

ECOLI ------------------------------------------------------------

HELPY ------------------------------------------------------------

NOSTOC2 ------------------------------------------------------------

SYNE ------------------------------------------------------------

BACS ------------------------------------------------------------

HOMO ------------------------------------------------------------

MOUSE ------------------------------------------------------------

SACC ------------------------------------------------------------

SCHP ------------------------------------------------------------

Ciliate ------------------------------------------------------------

NOSTOC1 ------------------------------------------------------------

RICP ------------------------------------------------------------

PLAF ------------------------------------------------------------

ARAT ---------------------------MATTPIFNASCSFPSTRGIDCKSYIGLRSNVSK 33

PEA ------------------------------------------------HTFVDLKSPFTL 12

SYNOWH ------------------------------------------------------------

CHLRE -------------------------------------------------------MQMMQ 5

CYAPA -------------------------------------------MGTRFCIASPALPAARS 17

ODOSI ---------------------------------MKFASVILATFAGVACAFAPSTPAFS- 26

THAPS ------------------------------------------------------------

CYAME -------------------------------------------------MFVAKVPLSI- 10

TOXO RSRASEPEIRQLAASPIFGAPKPFSSCPCSSSSRSSCAAHPSALLPEVCSFVSRRGARRN 240

Wolb ------------------------------------------------------------

wMel ------------------------------------------------------------

wAna ------------------------------------------------------------

wSim ------------------------------------------------------------

CAUL ------------------------------------------------------------

AGRO ------------------------------------------------------------

RHOD ------------------------------------------------------------

NEIME ------------------------------------------------------------

PSEAE ------------------------------------------------------------

ECOLI ------------------------------------------------------------

HELPY ------------------------------------------------------------

NOSTOC2 ------------------------------------------------------------

SYNE ------------------------------------------------------------

BACS ------------------------------------------------------------

HOMO ------------------------------------------------------------

MOUSE ------------------------------------------------------------

SACC ------------------------------------------------------------

SCHP ------------------------------------------------------------

Ciliate ------------------------------------------------------------

NOSTOC1 ------------------------------------------------------------

RICP ------------------------------------------------------------

PLAF ---------------------------------------MLKSDVVLLLYILIINLICCL 21

ARAT VS--------VASSRIATSQRRNLVVRASESGNGHAKKLGMSDAECEAAVAAGNVP---- 81

PEA SN--------YLS--FSSSKRRQPPSLFTVR---------ASDSDFEAAVVAGKVP---- 49

SYNOWH ------------------------------------------------------------

CHLRE RN--------VVGQRPVAGSRRSLVV--------------ANVAEVTRPAVSTNG----- 38

CYAPA IA--------VASSVSPLAAKPSQFDEFEGLRKKTFLHDGAVASRQFVAQTSGIKM---- 65

ODOSI -----------RSSALYSAVEDEVTTGPVAAGSIDRSLSGTDSSLSYDAFDPTSG----- 70

THAPS ------------------------------MEPVDRTMNGIDDNLGYEAFDPTAG----- 25

CYAME -----------RGQPSLNAIKPSAT----APKRRKLSLFAVAAPRRSSEQASTSA----- 50

TOXO LGAQRGRGPGARGQRLGAAAGPTPVGKGTPGGAKGQFRGGMSAQKGAMHQEPLRGRLEED 300

Wolb ------------------------------------------------------------

wMel ------------------------------------------------------------

wAna ------------------------------------------------------------

wSim ------------------------------------------------------------

CAUL ------------------------------------------------------------

AGRO ------------------------------------------------------------

RHOD ------------------------------------------------------------

NEIME ------------------------------------------------------------

PSEAE ------------------------------------------------------------

ECOLI ------------------------------------------------------------

HELPY ------------------------------------------------------------

NOSTOC2 ------------------------------------------------------------

SYNE ------------------------------------------------------------

BACS ------------------------------------------------------------

HOMO ------------------------------------------------------------

MOUSE ------------------------------------------------------------

SACC ------------------------------------------------------------

SCHP ------------------------------------------------------------

Ciliate ------------------------------------------------------------

NOSTOC1 ------------------------------------------------------------

RICP ------------------------------------------------------------

PLAF NGNSKKRAYILNTPKSSNCKRSSFRRWNNPVNNNNSQILSKNEGSIEDVYN--------- 72

ARAT ---------------------------------EAPPVPPKPAAPVGTPIIKPLNLSRRP 108

PEA ---------------------------------EAPPVPPTPASPAGTPVVPSLPIQRRP 76

SYNOWH -----------------------------------------------------MELTYRP 7

CHLRE --------------------------------------KHRTGVPEGTPIVTPQDLPSRP 60

CYAPA ---------------------------------QAGHASVVSGPSSARGSSYALAMRRRP 92

ODOSI ----------------------------------DSPALKR-----NNADEVWVSQRARP 91

THAPS ----------------------------------ENPAVAR-----NNQGGVWVQQRARP 46

CYAME ----------------------------------NSPLLPR-----NDKGEPWVVQRSRP 71

TOXO EEEEDEFEDDEFDDEDEGEDEGEDEEYDEDDDEDYDPMTPRGPLDNNNYGEVWLPIQARP 360

Wolb --------------------------------------------------MMFNFPNTRL 10

wMel ---------------------------------------------------MFNFPNTRL 9

wAna ---------------------------------------------------MFNFPNTRL 9

wSim ---------------------------------------------------MFNFPNTRL 9

CAUL --------------------MTTPP-----LAPYP---------------------HTRL 14

AGRO --------------------MSHSHGPMFMVCSFPNFIEGHSDMQDKTHLVDEITGHRRM 40

RHOD --------------------MTLITPPFP---------------------------TNRL 13

NEIME ---------------MIGGLMQFPYRNVS---------------------------ASRM 18

PSEAE ------------------MSFTPANRAYP---------------------------YTRL 15

ECOLI ----------------------------------------------------MTDLIQRP 8

HELPY -------------------------------------------------------MFKRL 5

NOSTOC2 -----------------------------------------------------MFPTHRP 7

SYNE -----------------------------------------------------MFPTIRP 7

BACS ----------------------------------------------------MSQSFNRH 8

HOMO -----------------------------------------------------MQPQSVL 7

MOUSE -----------------------------------------------------MHHQSVL 7

SACC ------------------------------------------MHTAEFLETEPTEISSVL 18

SCHP ---------------------------------------------------MPVDISSLL 9

Ciliate ----------------------------------------------MQPNSFELPISHAL 14

NOSTOC1 ------------------------------------------MSVENSSTAKPLNLLQRP 18

RICP -----------------------------------------------------MYPLIRF 7

PLAF --------------------------------KKISGRCNIKNFSKDINNNIYIETNRRE 100

ARAT RRNRASPVTRAAFQET-DISPANFVYPLFIHE--GEEDTPIGAMPGCYRLGWRHGLVQEV 165

PEA RRNRRSPALRSAFQET-TLSPANFVYPLFIHE--GEEDTPIGAMPGCYRLGWRHGLLEEV 133

SYNOWH RRLRRTPALRAMVREH-QLSSADFIYPLFVHE--GTDVEPIGAMPGASRWSLAA-LTGEV 63

CHLRE RRNRRSESFRASVREV-NVSPANFILPIFIHEE-SNQNVPIASMPGINRLAYGKNVIDYV 118

CYAPA RRNRKGAAMRGMYRET-TVGPENFILPLFIHD--GDDNQPISAMPGCFRLSREG-LLKEV 148

ODOSI RRNRKSAAVRGMVREN-IVTPANFIYPLFIHE--EDFNTEIPSMPGCERHSLPN-MLKEV 147

THAPS RRNRKSAGVRAMVREN-IVTPSNFIYPLFIHD--EDFCQDISSMPGCQRHSLPS-MLNEV 102

CYAME RRNRKTNAVREMVREN-VVSPGNLVYPVFIHD--EDHDEDIDVMPGCKRLSIQS-LLKHA 127

TOXO RRNRKNRAVRQLVQEN-LVKPSSLIYPLFVHD--EETSVPIPSMPGQSRLSMED-LLKEV 416

Wolb RRRRSSKWVRNLTSES-ALSVNDLIFPLFVHDR-EETTELVSSLPGMKCYSIDG-LVSIA 67

wMel RRRRSSKWVRNLTSEN-SLSVNDLVLPLFVHDR-EETTEPISGLPGVKCYSIDG-LVSIV 66

wAna RRRRSSKWVRNLTSEN-SLSVNDLVLPLFVHDR-EETTEPISGLPDVKCYSIDG-LVSIV 66

wSim RRRRSSKWVRNLTSEN-SLSVNDLVLPLFVHDR-EETTEPISGLPDVKCYSIDG-LVSIV 66

CAUL RRVRQSDWVRRLVRET-EVRPSDLIWSMVVHEG-EGTIP-VASMPGVERLSVKE-AAKAA 70

AGRO RRNRKADWTRRLVQEN-RLTVDDLIWPVFIVPG-SNIVEPIPAMPGVNRMSVDR-LVEAA 97

RHOD RRMRRTEALRDLAQEN-RLSVKDLIWPIFITDV-PGADVEISSMPGVVRRTMDG-ALKAA 70

NEIME RRMRRDDFSRRLMREH-TLTADDLIYPVFVLEG-SAREEDVPSMPGVKRQSLDR-LLFTA 75

PSEAE RRNRRDDFSRRLVREN-VLTVDDLILPVFVLDG-VNQRESIPSMPGVERLSIDQ-LLIEA 72

ECOLI RRLRKSPALRAMFEET-TLSLNDLVLPIFVEEEIDDYK-AVEAMPGVMRIPEKH-LAREI 65

HELPY RRLRSSENLRAMLRET-RLNIDDFIAPLFVIESDSCIKNEISSMPGVYQMSMEP-LLKEC 63

NOSTOC2 RRLRTHPQLRRMVRET-VLTTNDLIYPLFAVPG-EGIANEVKSMPGVYQLSVDK-IVEEA 64

SYNE RRLRQTDVLRRMVREN-TLTVNDLIYPLFAVPG-NAIAKEVVSMPGVYQLSVDK-IVDEA 64

BACS RRLRTSKAMREMVKET-RLHPSDFIYPIFVVEGLEGKK-AVPSMPDVHHVSLDL-LKDEV 65

HOMO HSGYFHPLLRAWQTATTTLNASNLIYPIFVTDVPDDIQ-PITSLPGVARYGVKR-LEEML 65

MOUSE HSGYFHPLLRSWQTAASTVSASNLIYPIFVTDVPDDVQ-PIASLPGVARYGVNQ-LEEML 65

SACC AGGYNHPLLRQWQSER-QLTKNMLIFPLFISDNPDDFT-EIDSLPNINRIGVNR-LKDYL 75

SCHP HAGYSNPLLREWQGVR-PITKSSLMYPIFISEIDDAKE-AIDSMPNQFRWGVNR-LEEFL 66

Ciliate HGGYKHPACRNWVKQT--LSANNLVYPVFVLDEPDAEQ-EIKTMPGIKRFGYKK-IVDHL 70

NOSTOC1 RRLRRTATLRRMVRET-TLTVDDLIYPMFVMEG-EGQKVEITSMPGCYRYSLDL-LLKEI 75

RICP RRNRKALWLRELIAES-NLSISDLVLPLFVVEG-HNEMQEIKTMPGIYRLSIDQ-IVETA 64

PLAF RRIKRNKYLLSLYNNT-NIKTSNFIYPLFIHEE--DVEKKHTQLEGIYTYNVDG-IIKEI 156

: :: .:. : .

ARAT AKARAVGVNSIVLFPKV-PEALKNSTGDEAYND--NGLVPRTIRLLKDKY-PDLIIYTDV 221

PEA AKARDVGVNSVVLFPKI-PDALKTPTGDEAYNE--DGLVPRSIRLLKDKY-PDLIIYTDV 189

SYNOWH QRAWNLGVRCIVLFPKV-AEGLKTEDGAECFNA--NGLIPRAIRQIKQEV-PGMAIMTDV 119

CHLRE AEPRSYGVNQVVVFPKT-PDHLKTQTAEEAFNK--NGLSQRTIRLLKDSF-PDLEVYTDV 174

CYAPA EGAYSEGIRAVVLFPKV-PDNLKTSDGAECFNP--DGIVPRTVRMLKEKF-PELLVITDI 204

ODOSI GEAYDLGVKTFVLFPKV-PDELKTNLGVEAYNP--DGIVHRAIRMIKEAY-PDSVVCTDV 203

THAPS GEALELGVKAFVLFPKV-DDALKTNLACEAYNP--EGIVHRSIRMIKEKY-PEAIVCTDV 158

CYAME AQALDLGIGHIILFPKI-EEHLKSNTADECYNP--KGLVPSAIKALKEKFGSRITVWTDI 184

TOXO GEARSYGIKAFMLFPKV-DDELKSVMAEESYNP--DGLLPRAIMALKEAF-PDVLLLADV 472

Wolb QEAEDLGINAVAIFPVV-DSKLKSENAEEAYNS--DNLICKAIRAIKLKV-PGIGIIADV 123

wMel KEAKDLGINAVAIFPVV-DSKLKSENAEEAYNS--DNLICRAICAVKLKV-PEIGIIADV 122

wAna KEAKDLGINAVAIFPVV-DSKLKSENAEEAYNS--DNLICRAICAVKLKV-PEIGIIADI 122

wSim KEAKDLGINAVAIFPVV-DSKLKSENAEEAYNS--DNLICRAICAVKLKV-PEIGIIADI 122

CAUL VRARDLGIPAIAIFPHI-DGSRKDAAGSIAADP--DGVIPRAVKAMKDAA-PEVGIMCDV 126

AGRO KEAADLGIPAIATFPNI-EMELRDETGSNALEA--NNLINQATIAVKKAV-PNIGMITDV 153

RHOD EEAATLGIPAICLFPYT-DPAVKTETCEMAWQP--DNFTNRVIAAMKQAV-PEVAIMTDI 126

NEIME EEAVKLGIPMLALFPVV--TANKTERAQEAYNP--EGLVPSTVRALRERF-PELGIMTDV 130

PSEAE EEWVALGIPALALFPVT-PVEKKSLDAAEAYNP--EGIAQRATRALRERF-PELGIITDV 128

ECOLI ERIANAGIRSVMTFGIS---HHTDETGSDAWRE--DGLVARMSRICKQTV-PEMIVMSDT 119

HELPY EELVGLGVKAVLLFGIP---KHKDATGSHALNK--DHIVAKAAKEIKKRF-KDLIVIADL 117

NOSTOC2 KEVHDLGIPAIILFGIP---ADKDTDATGAWHD--CGIVQKAATAVKAAV-PDLIVIADT 118

SYNE KEVRDLGIPAIILFGIP---EDKDTDATGAWHD--CGIVQKATEAVKKAV-PDLVVIVDT 118

BACS AELVKLGIQSVIVFGIP---EEKDDCGTQAYHD--HGIVQKAITEIKEHF-PEMVVVADT 119

HOMO RPLVEEGLRCVLIFGVP-SRVPKDERGSAADSE--ESPAIEAIHLLRKTF-PNLLVACDV 121

MOUSE RPLVEAGLRCVLIFGVP-SRVPKDEQGSAADSE--DSPTIEAVRLLRKTF-PSLLVACDV 121

SACC KPLVAKGLRSVILFGVPLIPGTKDPVGTAADDP--AGPVIQGIKFIREYF-PELYIICDV 132

SCHP GPLVKKGLRSVILFGVI--ESKKDYCGSMADSE--NGPVIKAVKEIRHLF-PELVVACDV 121

Ciliate KPLVKKGLQSIIIFGILSDDNKKDERGSHAGGNGIKGPTNLALEEIKKEI-PELLLIVDV 129

NOSTOC1 AEVSQLGIPAIALFPVI-SENKKDDIGAESYNP--EGLVQQTVKAIKQAV-PDIVVITDV 131

RICP KKAADFGINAIALFPSI-DHSLKSDNADEAYNL--DNLICRTIIKIKNAN-IDIGIICDV 120

PLAF EECIKLNIHHFMFFPVI-REENKTVYCEESYNE--NSYFCKTISRIKEKFSDDIIVYTDV 213

.: . * . : : *

ARAT ALDPYSSDGHDGIVRED-------GVIMNDETVHQLCKQAVSQARAGADVVSPSDMMDGR 274

PEA ALDPYSSDGHDGIVRED-------GVIMNDETVHQLCKQAVAQARAGADVVSPSDMMDGR 242

SYNOWH ALDPYSCDGHDGIVSPA-------GVVLNDETIELLCKQAVVQAEAGADLIGPSDMMDGR 172

CHLRE ALDPYNSDGHDGIVSDA-------GVILNDETIEYLCRQAVSQAEAGADVVSPSDMMDGR 227

CYAPA ALDPYNSDGHDGIVDKTN------GKIINDETVEQLCKQALCHAAAGADIVSPSDMMDGR 258

ODOSI ALDPYSDQGHDGVVED--------GKILNDVTINQLCKQAVSQARAGADVVAPSDMMDGR 255

THAPS ALDPYSDQGHDGVVED--------GVILNDVTVNQLCKQAVSQARAGADIVAPSDMQDGR 210

CYAME ALDPYSDQGHDGIVSNDSLGDGGCARILNDETVEQLCRQALCHARAGADIVAPSDMMDGR 244

TOXO ALDPYSSMGHDGVVDEQS------GKIVNDLTVHQLCKQAITLARAGADMVCPSDMMDGR 526

Wolb ALDPYTTHGHDGILKSNQ------IDVENDKTVSILCKQALALAKAGCNIVASSDMMDGR 177

wMel ALDPYTIHGHDGILKDNQ------MDVENDETISVLCKQALALAKAGCDIVAPSDMMDGR 176

wAna ALDPYTIHGHDGILKDNQ------MDVENDETISVLCKQALALAKAGCDIVAPSDMMDGR 176

wSim ALDPYTIHGHDGILKDNQ------MDVENDETISVLCKQALALAKAGCDIVAPSDMMDGR 176

CAUL ALDPFTDHGHDGVVEG--------GKILNDATIERLIEQGLMQADAGADILAPSDMMDGR 178

AGRO ALDPFTSHGHDGILRG--------DEIVNDETVEQVVTAAILQAEAGSDIISPSEMMDGR 205

RHOD ALDPYNANGHDGLVRD--------GIILNDETTEALVKMALAQAAAGADILGPSDMMDGR 178

NEIME ALDPYTVHGQDGLTDEN-------GYVMNDETVEVLVKQALCHAEAGAQVVAPSDMMDGR 183

PSEAE ALDPFTTHGQDGILDDD-------GYVLNDVSIDVLVRQALSHAEAGAQVVAPSDMMDGR 181

ECOLI **C**F**C**EYTSHGH**C**GVLCE--------HGVDNDATLENLGKQAVVAAAAGADFIAPSAAMDGQ 171

HELPY **C**F**C**EYTDHGH**C**GILEN--------ASVSNDKTLEILNLQGLILAESGVDILAPSNMMDGN 169

NOSTOC2 **C**L**C**EYTSHGH**C**GYLQVGDLT----GRVLNDPTLELLKKTAVSQAKAGADIIAPSGMMDGF 174

SYNE **C**L**C**EYTNHGH**C**GYLETGDLT----GRVLNDPTLELLKKTAVSQANAGADVIAPSGMMDGF 174

BACS **C**L**C**EYTDHGH**C**GLVKD--------GVILNDESLELLAQTAVSQAKAGADIIAPSNMMDGF 171

HOMO **C**L**C**PYTSHGH**C**GLLSEN-------GAFRAEESRQRLAEVALAYAKAGCQVVAPSDMMDGR 174

MOUSE **C**L**C**PYTSHGH**C**GLLSEN-------GAFLAEESRQRLAEVALAYAKAGCQVVAPSDMMDGR 174

SACC **C**L**C**EYTSHGH**C**GVLYDD-------GTINRERSVSRLAAVAVNYAKAGAHCVAPSDMIDGR 185

SCHP **C**L**C**EYTDHGH**C**GLLYED-------GTINNAKSVERIAEVSGNYALAGAQIISPSDCMDGR 174

Ciliate **C**L**C**AFTSHGH**C**GLLNCE-------GYIDNAASLKRLVEVSISYVQSGAQVIAPSDMMDGR 182

NOSTOC1 ALDPFTTHGHDGLVDEN-------GTILNDPTVEMLVKMALSQAAAGTDFVAPSDMMDGR 184

RICP ALDPYTISGHDGIVHH--------GEVDNDRSVKALCNQALVLAKAGVDIVAPSDMMDGR 172

PLAF ALDPYNIYGHDGIYDDNKK------EILNDITVHTLVKQSLCLAKSGADVVCPSDSMDKR 267

.: :. *: * . : : . . :* . : .* *

ARAT VGAIRSALDAEGFQ-NVSIMSYTAKYASSFYGPFREALDSNPRFG--------DKKTYQM 325

PEA VGAMRVALDAEGFQ-HVSIMSYTAKYASSFYGPFREALDSNPRFG--------DKKTYQM 293

SYNOWH VGAIREALDDEGFE-HVGIISYTAKYSSAYYGPFREALDSAPRAAGSKP-IPTNKDTYQM 230

CHLRE VGAIRRALDREGFT-NVSIMSYTAKYASAYYGPFRDALASAPKPGQAHRRIPPNKKTYQM 286

CYAPA IGAIRDALDGAGYT-DVSILSYCAKYASAYYGPFRTALDSDPRFG--------DKTTYQM 309

ODOSI VGAIRDALDAEGFT-DVSILAYTAKYASAYYGPFRDALDSHP--------GFGDKKTYQQ 306

THAPS VKAIRDALDSEGFT-NVSILSYTAKYASAYYGPFRDALDSHP--------GFGDKKTYQQ 261

CYAME VGAIRDALDEEGFT-DVSIVSYAAKYASSFYGPFRGALDSAPRETSR---APRDKKTYQM 300

TOXO VSAIRESLDMEGCT-DTSILAYSCKYASSFYGPFRDALDSHMVG-------GTDKKTYQM 578

Wolb VGRIRKVLDDNNLQ-DVSILSYAVKYCSSFYAPFRQIVGSCVSSNS------IDKSGYQM 230

wMel VGRIRKSLDDNNFQ-DVLILSYAVKYCSSFYAPFRQVVGSCGLSHS------IDKSGYQM 229

wAna IGRIRKSLDDNNFQ-DVLILSYAVKYCSSFYAPFRQVVGSCGLSHS------IDKSGYQM 229

wSim IGRIRKSLDDNNFQ-DVLILSYAVKYCSSFYAPFRQVVGSCGLSHS------IDKSGYQM 229

CAUL IGKLRAALEGANYQ-DVMIMSYAAKYASAFYGPYRDAIGSAKLSAGQ-----GDKKTYQM 232

AGRO IGAIRRGLDAAGHQ-NVGIMAYATKFSSGYYGPYREAISTAGLLKG-------DKNSYYI 257

RHOD VGAIRQAMEAAGHK-DIAILSYAAKYASAFYGPFRDAVGASSALKG-------DKKTYQM 230

NEIME IGAIREALEDAGHI-HTRIMAYSAKYASAFYGPFRDAVGSSGNLGK------ADKKTYQM 236

PSEAE IGAIREALESAGHT-NVRIMAYSAKYASAYYGPFRDAVGSASNLGK------GNKATYQM 234

ECOLI VQAIRQALDAAGFK-DTAIMSYSTKFASSFYGPFREAAGS---ALK------GDRKSYQM 221

HELPY VLSLRKTLDNAGYT-HTPIMSYSTKFASSYYGPFRDVANS---APSF-----GDRKSYQM 220

NOSTOC2 VQAIRAGLDEAGFQ-DTPILSYAAKYASAYYGPFRDAADS---TPQF-----GDRRTYQM 225

SYNE VQAIREALDDHDFQ-NIPILSYAAKYASAYYGPFRDAADS---SPQF-----GDRRTYQM 225

BACS VTVIREALDKEGFV-NIPIMSYAVKYSSEFYGPFRDAANS---TPQF-----GDRKTYQM 222

HOMO VEAIKEALMAHGLGNRVSVMSYSAKFASCFYGPFRDAAKS---SPAF-----GDRRCYQL 226

MOUSE VEAIKAALLKHGLGNRVSVMSYSAKFASCFYGPFRDAAQS---SPAF-----GDRRCYQL 226

SACC IRDIKRGLINANLAHKTFVLSYAAKFSGNLYGPFRDAACS---APSN-----GDRKCYQL 237

SCHP VKAIKQKLVELELSHKVCVISYSAKFASGFFGPFRAAANG---APKF-----GDRSCYQL 226

Ciliate IGAIKQELIRQNL--EIPVMSYAAKFASSYYGPFRDACKS---APGK-----SDRQAYQL 232

NOSTOC1 IGAIRQALDAEGYI-NVGILAYSAKYASAYYGPFRDALDS---APKF-----GDKKTYQM 235

RICP IAAIREYLDKEGFI-NVGILAYAAKYASSFYGPFRDAVQSNK-KNYL------DKSSYQI 224

PLAF IELIRKNLDFHNFR-DILILSYTCKYSSSMYKPFRSILNSNILKNFVK-----NKQSYQH 321

: :: : :::* *:.. : *:* :: *

ARAT NPANYREALIEAREDEAEGADILLVKPGLPYLDIIRLLRD----KSPLPIAAYQVSGEYS 381

PEA NPANYREALTEMREDESEGADILLVKPGLPYLDIIRLLRD----NSPLPIAAYQVSGEYS 349

SYNOWH DPANAREAITEAQLDEQEGADIMMVKPGLAYLDIIHRLRN----ESELPIAAYNVSGEYS 286

CHLRE DPANYREAIREAKADEAEGADIMMVKPGMPYLDVVRLLRE----TSPLPVAVYHVSGEYA 342

CYAPA DPANVREALIEAELDLEEGADMIMVKPGLPYLDVIKTLRE----NSPLPIAAYQVSGEYA 365

ODOSI DPANGREALIEAALDAAEGADMLMVKPGMPYLDIIRRLKD----ATPLPVAAYHVSGEYA 362

THAPS DPANGREALIEAALDAAEGADMLMVKPGMPYLDIIRRLKD----NSDLPIAAYHVSGEYA 317

CYAME DPGNAREALREAAADLEEGADMLLVKPGVPYLDIIHLLYD----AFPVPIAAYHVSGEYA 356

TOXO DPSNSREAEREAEADASEGADMLMVKPGLPYLDVLAKIRE----KSKLPMVAYHVSGEYA 634

Wolb DYRNAREAICEIEMDLNEGADFIMVKPGMPYLDIIKMASD----EFNFPIFAYQVSGEYA 286

wMel DYKNAHEAMCEIEMDINEGADFIMIKPGMPYLDIIKTASD----KFNFPIFAYQVSGEYA 285

wAna DYKNAREAMCEIEMDINEGADFIMIKPGMPYLDIIKTASD----KFNFPIFAYQVSGEYA 285

wSim DYKNAREAMCEIEMDINEGADFIMIKPGMPYLDIIKTASD----KFNFPIFAYQVSGEYA 285

CAUL DPANTEEAIREVALDIAEGADMVMVKPGMPYLDILRRLVE----EFRMPTYAFQVSGEYA 288

AGRO SPANGMEAIRDAALDVEEGADMLMVKPGLPYLDICWRLKE----NFGLPTFAYQVSGEYT 313

RHOD NPANSAEALRNVARDIAEGADMVMVKPGMPYLDIVRQVKD----AFGMPTYAYQVSGEYA 286

NEIME DPANTDEALHEVALDIQEGADMVMVKPGLPYLDVVRRVKD----EFGVPTYAYQVSGEYA 292

PSEAE DPANSDEALHEVAADLAEGADMVMVKPGMPYLDIVRRVKD----EFRAPTFVYQVSGEYA 290

ECOLI NPMNRREAIRESLLDEAQGADCLMVKPAGAYLDIVRELRE----RTELPIGAYQVSGEYA 277

HELPY DYANQKEALLESLEDEKQGADILMVKPALAYLDIVKEIRD----HTLLPLALYNVSGEYA 276

NOSTOC2 DPGNSREAIREIELDIAEGADMLMVKPALAYMDIIWQVKE----ASNLPVAAYNVSGEYS 281

SYNE DPGNSREALKEVELDLLEGADMVMVKPALSYMDIIWRIKE----MTNLPVAAYNVSGEYS 281

BACS DPANRMEALREAQSDVEEGADFLIVKPSLSYMDIMRDVKN----EFTLPLVAYNVSGEYS 278

HOMO PPGARGLALRAVDRDVREGADMLMVKPGMPYLDIVREVKDK---HPDLPLTVYHVSGEFA 283

MOUSE PPGARGLALRAVARDIQEGADMLMVKPGLPYLDMVREVKDK---HPELPLAVYQVSGEFA 283

SACC PPAGRGLARRALERDMSEGADGIIVKPSTFYLDIMRDASEI---CKDLPICAYHVSGEYA 294

SCHP PCNARGLAKRAILRDVREGADGIMVKPGTPYLDILAMASKL---ADDLPIATYQVSGEFA 283

Ciliate PLDSTQLALKAVQRDLEEGADYIMVKPITTYLDIVKEIKQR---FNPI-MACYHVSGEYA 288

NOSTOC1 DAANAREAIKEVELDIVEGADIVMVKPALAYLDIIHQVQQ----ATQLPVAAYNVSGEYA 291

RICP DVRNIKEAMLEIEHDIAEGADIVMVKPGMPFLDVIREAAN----NFNAKIFAYQVSGEYA 280

PLAF DFN-SYMDLNNVDKHIIEGADIIMVKPSMFYLDIIHKIKNRIKDDVQIPIAVYNVSGEYM 380

. :*** :::** ::*: . ::****:

ARAT MIKAGGVLK---MIDEEKVMMESLMCLRRAGADIILTYFALQAATCLCGEKR-------- 430

PEA MIKAGGALK---MIDEEKVMMESLLCLRRAGADIILTYFALQAARTLCGEKR-------- 398

SYNOWH MVKAAAERG---WIDERAVVLETLLSFKRAGADLILTYHACDAAEWLRQG---------- 333

CHLRE MLKAAAERG---WLNEKDAVLEAMTCFRRAGGDLILTYYGIEASKWLAGEK--------- 390

CYAPA MIKAAVANG---WLDERKVVMESLIALRRAGADFILTYFARPASRWIAEDREAARKY--- 419

ODOSI MIKAACEKG---WLEEKDVVLETLTCFKRAGADIILTYYAKQAAQWIKDDGLY------- 412

THAPS MLKAAVEKG---WLDEKAVVLETLTCFKRAGADIILTYYAKQAAKWIKEDGLY------- 367

CYAME MIKAAAQRG---WIDEKQVVLETLLSLKRAGATSILTYYALEAAKWMQE----------- 402

TOXO MLKAAAEKG---YISEKDTVLEVLKSFRRAGADAVATYYAKEAAKWMVEDMKGTQKFTEP 691

Wolb MIKAATNNG---WLDYDKVIYESLVGFKRAGASAIFTYAALDVAKNLR------------ 331

wMel MIKAAANNG---WLDYDKVIYESLIGFKRAGASAIFTYAALDIAKNLSA----------- 331

wAna MIKAAANNS---WLDYDKVIYESLIGFKRAGASAIFTYAALDIAKNLSA----------- 331

wSim MIKAAANNS---WLDYDKVIYESLIGFKRAGASAIFTYAALDIAKNLSA----------- 331

CAUL MIMAAAQNG---WIDKDRAILESLTAFKRAGAAGIITYFAPWAAEKLGA----------- 334

AGRO QIKAAAMNG---WIDGERVMMETLLCFKRAGCDGILTYFAIEAARKLAKH---------- 360

RHOD MLMAAVQNG---WLNHDKVMLESLMAFRRAGCDGVLTYFAPAAAKLIGA----------- 332

NEIME MLQAAVANG---WLDGGKVVLESLLAFKRAGADGILTYYAIEAAKMLKR----------- 338

PSEAE MHMGAIQNG---WLAES-VILESLTAFKRAGADGILTYFAKQAAEQLRRGR--------- 337

ECOLI MIKFAALAG---AIDEEKVVLESLGSIKRAGADLIFSYFALDLAEKKILR---------- 324

HELPY MLKLAQKHN---LINYESVLLETMTCFKRAGADMIISYHAKEVANLLQRN---------- 323

NOSTOC2 MVKAAALNG---WIDEQRVVMETLTGFKRAGADLILTYHAKDAARWLP------------ 326

SYNE MVKAAALNG---WIDEQKVTLETLTSFKRAGADLILTYHAKDAARWLQD----------- 327

BACS MVKAAAQNG---WIKEKEIVLEILTSMKRAGADLIITYHAKDAAKWLAE----------- 324

HOMO MLWHGAQAG---AFDLKAAVLEAMTAFRRAGADIIITYYTPQLLQWLKEE---------- 330

MOUSE MLWHGAQAG---AFDLRTAVLETMTAFRRAGADIIITYFAPQLLKWLKEE---------- 330

SACC MLHAAAEKG---VVDLKTIAFESHQGFLRAGARLIITYLAPEFLDWLDEEN--------- 342

SCHP IIHAAAAAG---VFELKRHVMETMDGFMRAGANIVLTYFTPELLEWLEY----------- 329

Ciliate MVCFAAQNG---ACDKKKVVLETMRSFRRAGIDIIITYFTPELLDWVKEEF--------- 336

NOSTOC1 MIKAAAQMG---WINEKQVILESLTSMKRAGADLILTYFAKEVALMLL------------ 336

RICP MLKFAAETG---AIDWERALIESLISFKRAGATGIFTYAALEVAEILKHNNIK------- 330

PLAF MIKNYVKYLNEDINYENEIITELFKSYLRAGANIIITYFAKQYGLYMKKLYDKNIIIDDN 440

* *** : :*

ARAT -----------

PEA -----------

SYNOWH -----------

CHLRE -----------

CYAPA -----------

ODOSI -----------

THAPS -----------

CYAME -----------

TOXO CY--------- 693

Wolb -----------

wMel -----------

wAna -----------

wSim -----------

CAUL -----------

AGRO -----------

RHOD -----------

NEIME -----------

PSEAE -----------

ECOLI -----------

HELPY -----------

NOSTOC2 -----------

SYNE -----------

BACS -----------

HOMO -----------

MOUSE -----------

SACC -----------

SCHP -----------

Ciliate -----------

NOSTOC1 -----------

RICP -----------

PLAF SNNNFNIELTL 451

**CLUSTAL X (1.83) multiple sequence alignment for PBGD (3)**

**26 taxa/203 characters from underlined conserved regions are used for phylogeny analysis**

**Legend for abbreviations used in the alignment:**

**ARATH** Q43316 382aa Porphobilinogen deaminase Arabidopsis thaliana plants

**PEA** Q43082 369aa Porphobilinogen deaminase, Pisum sativum (pea) Plants

**CYAME*** 417aa CME132C retrieved from http://merolae.biol.s.u-tokyo.ac.jp/ Cyanidioschyzon merolae Genome Project, Red algae

**TOXO** DQ029341 747 aa Porphobilinogen deaminase Toxoplasma gondii Apicomplexa

**PLAYO** EAA21199 583aa porphobilinogen deaminase, Plasmodium yoelii yoelii Apicomplexa

**PLAFA** AE014845 435aa porphobilinogen deaminase, Plasmodium falciparum 3D7 Apicomplexa

**SYNY** P73660 320aa Porphobilinogen deaminase Synechocystis sp. PCC 6803 Cyanobacteria

**SYNC** Q8DIE4 320aa Porphobilinogen deaminase Synechococcus elongatus Cyanobacteria

**RICPR** Q9ZD77 299aa Porphobilinogen deaminase Rickettsia prowazekii Alpha-proteobacteria

**RHOCA** AAG50298 porphobilinogen deaminase [Rhodobacter capsulatus] Alpha-proteobacteria

**AGRTU** Q8UC46 309aa Porphobilinogen deaminase Agrobacterium tumefaciens str. C58 Alpha-proteobacteria

**CAUCR** NP_418891 322aa porphobilinogen deaminase [Caulobacter crescentus CB15] Alpha-proteobacteria

**Wolb** AAW71365 292aa Porphobilinogen deaminase [Wolbachia endosymbiont strain TRS of Brugia malayi] Alpha-proteobacteria

**wAna** EAL58153 267aa Porphobilinogen deaminase [Wolbachia endosymbiont of Drosophila ananassae] Alpha-proteobacteria

**wWill** ZP_01314577 302aa Porphobilinogen deaminase [Wolbachia endosymbiont of Drosophila willistoni TSC#14030-0811.24] Alpha-proteobacteria

**wMel** NP_966318 292aa Porphobilinogen deaminase [Wolbachia endosymbiont of Drosophila melanogaster] Alpha-proteobacteria

**NEIME** Q9K0P6 311aa Porphobilinogen deaminase Neisseria meningitidis serogroup B, Beta-proteobacteria

**ESCCO** NP_418249 320aa porphobilinogen deaminase = hydroxymethylbilane synthase [Escherichia coli K12] Gamma-proteobacteria

**HELPY** P56140 296aa PORPHOBILINOGEN DEAMINASE Helicobacter pylori Epsilon-proteobacteria

**BACSU** NP_390693 314aa porphobilinogen deaminase (hydroxymethylbilane synthase) [Bacillus subtilis] Firmicutes/G+ bacteria

**SACCE** CAA77804 327aa porphobilinogen deaminase [Saccharomyces cerevisiae] Fungi

**SCHPO** Q09899 336aa Porphobilinogen deaminase Schizosaccharomyces pombe (fission yeast) Fungi

**HOMO** P08397 361aa Porphobilinogen deaminase Homo sapiens Mammal

**MUS** P22907 361aa PORPHOBILINOGEN DEAMINASE Mus musculus Mammal

**Ciliate** EAS01157 362aa Porphobilinogen deaminase retrieved from http://tigrblast.tigr.org/tgi/TIGR "Tetrahymena thermophila genome sequencing project" Tetrahymena thermophila Ciliate

**THAPS** EED89447 330aa Porphobilinogen deaminase genewise.37.3.1 retrieved from http://genome.jgi-psf.org/thaps1/thaps1.home.html Thalassiosira pseudonana Diatoms/Heterokonts

ARATH ------------------------------------------------------------

PEA ------------------------------------------------------------

CYAME ------------------------------------------------------------

CAUCR ------------------------------------------------------------

TOXO MTLLREQRSAGAALRLSWVAFLFGFLVLLRDSLFKESYETPAVLLFAAARLLAPPLTPRA 60

SYNY ------------------------------------------------------------

SYNC ------------------------------------------------------------

HOMO ------------------------------------------------------------

MUS ------------------------------------------------------------

SACCE ------------------------------------------------------------

SCHPO ------------------------------------------------------------

NEIME ------------------------------------------------------------

ESCCO ------------------------------------------------------------

RICPR ------------------------------------------------------------

Wolb ------------------------------------------------------------

wAna ------------------------------------------------------------

wMel ------------------------------------------------------------

wWill ------------------------------------------------------------

PLAYO ------------------------------------------------------------

PLAFA ------------------------------------------------------------

Ciliate ------------------------------------------------------------

RHOCA ------------------------------------------------------------

AGRTU ------------------------------------------------------------

THAPS ------------------------------------------------------------

HELPY ------------------------------------------------------------

BACSU ------------------------------------------------------------

ARATH -----------------MDIASSSLSQAHKVVLTRQPSSRVNTCSLGSVSAIGFSLPQIS 43

PEA -----------------MEMTLYSSSSFSLPSAPSNPSLSLFTS--------SFRFSSFK 35

CYAME -----------------MLWGFVSVTAAVATGTQQRTRARVGCSPRALLLTAPTRHRLCK 43

CAUCR ------------------------------------------------------------

TOXO GQGGASSASSSSRLSPSARFPAASWSSSAALVSLLSPPASSRASPSRRNADVPSPSFAVT 120

SYNY ------------------------------------------------------------

SYNC ------------------------------------------------------------

HOMO ------------------------------------------------------------

MUS ------------------------------------------------------------

SACCE ------------------------------------------------------------

SCHPO ------------------------------------------------------------

NEIME ------------------------------------------------------------

ESCCO ------------------------------------------------------------

RICPR ------------------------------------------------------------

Wolb ------------------------------------------------------------

wAna ------------------------------------------------------------

wMel ------------------------------------------------------------

wWill ------------------------------------------------------------

PLAYO -----------------------------MHILTFIILNIYITICVCIHARIKTENFINI 31

PLAFA -----------------------------MHLLSFLSFIIWFIHCTAKRHEYSIKKYF-L 30

Ciliate ------------------------------------------------------------

RHOCA ------------------------------------------------------------

AGRTU ------------------------------------------------------------

THAPS ------------------------------------------------------------

HELPY ------------------------------------------------------------

BACSU ------------------------------------------------------------

ARATH -SPALGKCRRKQSSSGFVKACVAVEQKT----------RTAIIRIGTRGSPLALAQA--- 89

PEA -TSPFSKCRIRAS-----LAVEQQTQQN----------KTALIRIGTRGSPLALAQA--- 76

CYAME GTGPASKARTAECITSGPRTWRATVAQCPVDASTRRRENPAIVVIGTRGSPLALAQA--- 100

CAUCR ------------------------------------MSRQPPIRIGARGSKLSLAQS--- 21

TOXO ARPLARSSRESRLSPLAFLPVSASQNAVQTTPPFSGASEKTPLRVGSRASPLALAQARQF 180

SYNY ---------------------------------MTVSTSAPTVRIGSRKSQLALVQT--- 24

SYNC ----------------------------------MIATSSRPVRIGSRKSQLALVQT--- 23

HOMO ----------------------MSGNGNAAATAEENSPKMRVIRVGTRKSQLARIQT--- 35

MUS ----------------------MSGNGGAATTAEENGSKMRVIRVGTRKSQLARIQT--- 35

SACCE -------------------------------------MGPETLHIGGRKSKLAVIQS--- 20

SCHPO ------------------------------------MPSCTSFPIGTRKSKLAVIQS--- 21

NEIME -------------------------------------MNPKKLVIASRESLLAMWQA--- 20

ESCCO ------------------------------MIMTVTSMLDNVLRIATRQSPLALWQA--- 27

RICPR --------------------------------------MINSIRIGTRNSTLALIQT--- 19

Wolb ----------------------------------------MLIRIGTRGSSLAIAQA--- 17

wAna ----------------------------------------MLVKIGTRGSKLAVVQA--- 17

wMel ----------------------------------------MLVKIGTRGSKLAVAQA--- 17

wWill ------------------------------MNDIRQTIVHMLVKIGTRGSKLAVAQA--- 27

PLAYO NKSEFGKQKNKLAIKKNFYTHKNFKKKGKKKKIFLVQATK-EIIIGTRNSPLAIKQS--- 87

PLAFA NSHNFCKIK-----------PDPFRKDTLKKRLYSSDGIKDEIIIGTRDSPLALKQS--- 76

Ciliate ----------------------MDLKDQSLDQQLIDSLKNRKFKIASRSSQLALSQT--- 35

RHOCA --------------------------------MEQMPSPNAPLKIGTRGSPLALAQA--- 25

AGRTU -------------------------------------MQTKPFRIGTRGSPLALAQA--- 20

THAPS ------------------------------------------IRIGTRGSPLALAQA--- 15

HELPY ---------------------------------------MGNLVIGSRGSELALWQA--- 18

BACSU --------------------------------------MMRTIKVGSRRSKLAMTQT--- 19

. :. * * *: *:

ARATH ------------------------------------------------------------

PEA ------------------------------------------------------------

CYAME ------------------------------------------------------------

CAUCR ------------------------------------------------------------

TOXO VYRLALRFPDAFPQAHEIIQASLAKLSNPLPSSSSSDSSSSSSASPSSSSSASSSSSSSP 240

SYNY ------------------------------------------------------------

SYNC ------------------------------------------------------------

HOMO ------------------------------------------------------------

MUS ------------------------------------------------------------

SACCE ------------------------------------------------------------

SCHPO ------------------------------------------------------------

NEIME ------------------------------------------------------------

ESCCO ------------------------------------------------------------

RICPR ------------------------------------------------------------

Wolb ------------------------------------------------------------

wAna ------------------------------------------------------------

wMel ------------------------------------------------------------

wWill ------------------------------------------------------------

PLAYO ------------------------------------------------------------

PLAFA ------------------------------------------------------------

Ciliate ------------------------------------------------------------

RHOCA ------------------------------------------------------------

AGRTU ------------------------------------------------------------

THAPS ------------------------------------------------------------

HELPY ------------------------------------------------------------

BACSU ------------------------------------------------------------

ARATH ---YETREKLKKKHPELVE---------------------DGAIHIEIIKTTGDK----I 121

PEA ---HETRDKLMASHTELAE---------------------EGAIQIVIIKTTGDK----I 108

CYAME ---HETKRLLEEAHPFLRDR--------------------EGAIHIEIIHTTGDI----V 133

CAUCR ---GLMQARIAHALGVPAGASKDEI---------------EAAAPLIPIVTSGDR----I 59

TOXO ANSHLASSAFSSVLASLLRVDSRLE---------------YGELRIVPMQTTGDK----E 281

SYNY ---YWVQEELQKHFPD-------------------------RQFDVETMETQGDK----I 52

SYNC ---EWVQAQLQTHHRD-------------------------RAFEVVTMTTQGDN----I 51

HOMO ---DSVVATLKASYPG-------------------------LQFEIIAMSTTGDK----I 63

MUS ---ETVVAMLKALYPG-------------------------IQFEIIAMSTTGDK----I 63

SACCE ---NHVLKLIEEKYPD-------------------------YDCKVFTLQTLGDQ----I 48

SCHPO ---EIIREELEKHYPH-------------------------LEFPIISRDTIGDE----I 49

NEIME ---KHIQGRLKALYPD-------------------------CEVEILGMTTRGDQ----I 48

ESCCO ---HYVKDKLMASHPG-------------------------LVVELVPMVTRGDV----I 55

RICPR ---NLVIEQIKQIFPD-------------------------INCEIVPIITSGDL----I 47

Wolb ---LEAKQKLLDLFSS-------------------------LSVEIIKIKTSGDK----Y 45

wAna ---LEAKQKLLDSFPN-------------------------LSIEIVKIKTSGDK----Y 45

wMel ---LEAKQKLLDSFPN-------------------------LSIEIVKIKTSGDK----Y 45

wWill ---LEAKQKLLDSFPN-------------------------LSIEIVKIKTSGDK----Y 55

PLAYO ---EKVKKKLLTYFKKINEN---------------------INVILKPIKTTGDK----I 119

PLAFA ---EKVRKKIMSYFKKMNKN---------------------INVTFKYIKTTGDN---IL 109

Ciliate ---YEVIDLLVGVKELGLTKENFEVVPISNAPGKNCTLKFKINITNKQFTNKGDQ----N 88

RHOCA ---FETRSRLMAAF-----------------------DLPEEAFEIVVIKTSGDNAALIA 59

AGRTU ---YETRSRLMAAH-----------------------GLPEEMFEIVVLSTKGDR----I 50

THAPS ---YETRRRLIENFPE---------------------LEAEGAIEICVMKTQGDM----I 47

HELPY ---NHIKERLKKEC--------------------------LIESEIQIVKTKGDK----I 45

BACSU ---KWVIQKLKEINPS-------------------------FAFEIKEIVTKGDR----I 47

: . **

ARATH LSQPLADIG---GKGLFTKEIDEALING----HIDIAVHSMKDVPTYLPEKTILPCNLPR 174

PEA LSQPLADIG---GKGLFTKEIDEALING----DIDIAVHSMKDVPTYLPEETILPCNLPR 161

CYAME LDRALSEIG---GKGLFTREIDEAQLRG----DIDIAVHSMKDVPTFLPPDIELTSILRR 186

CAUCR QDRRLMEIG---GKGLFTKEIEEALLDG----RIDCAVHSLKDMPAELPPGLVLAATPER 112

TOXO LHLPLAQVG---GKGLFTKELDLALLHG----QVDVVVHSLKDVPTELPEGTEIGAYLPR 334

SYNY LDVALAKIG---DKGLFTQELEDGMLGK----RTDLAVHSLKDLPTNLPAGLMLGCVTKR 105

SYNC LDVALAKIG---DKGLFTKELELSMLRG----ETDLAVHSLKDLPTQLPDGLVLAAITER 104

HOMO LDTALSKIG---EKSLFTKELEHALEKN----EVDLVVHSLKDLPTVLPPGFTIGAICKR 116

MUS VDTALSKIG---EKSLFTKELENALEKN----EVDLVVHSLKDVPTILPPGFTIGAICKR 116

SACCE QFKPLYSFG---GKALWTKELEDHLYHDDPSKKLDLIVHSLKDMPTLLPEGFELGGITKR 105

SCHPO LSKALFEFKRQLAKSLWTRELEALLVTN----QCRILVHSLKDLPSEMPDGMVIACIPKR 105

NEIME LDKTLSKVG---GKGLFVKELEQALYDG----RADLAVHSIKDVPMDLPEGFALAAIGER 101

ESCCO LDTPLAKVG---GKGLFVKELEVALLEN----RADIAVHSMKDVPVEFPQGLGLVTICER 108

RICPR QNKPLYDIG---GKALFLKEIEQALLDK----KIDLAVHSLKDIPGKIPVELVIAAVLER 100

Wolb ANANLAEIG---GKGLFIKEIETELIKS----NIDMAVHSLKDVPAFFSKDLTIPCILER 98

wAna ANANLAEIG---GKGLFIKEIEAELLEN----NIDMAVHSLKDVPAFFSWGLTIPCVLER 98

wMel ANANLAEIG---GKGLFIKEIETELLEN----NIDMAVHSLKDMPAFFSGGLTIPCVLER 98

wWill ANANLAEIG---GKGLFIKEIETELLEN----NIDMAVHSLKDMPAFFSGGLTIPCVLER 108

PLAYO LDKTVGSFG---GKGIFTKELDEELIKN----NVDICVHSLKDIPTVLPDNIHLSCFLKR 172

PLAFA DSKSVGLYG---GKGIFTKELDEQLING----NVDLCVHSLKDVPILLPNNIELSCFLKR 162

Ciliate LKDPLYVMG---GVGVFTKIVEVELLNK----NGDIAVHSLKDLPTIIDERLFIGAVPPL 141

RHOCA ADKPLKEVG---GKGLFTKEIEEAMLAG----SIDIAVHSMKDMPTLQPEGLILDCYLPR 112

AGRTU TDRALSEIG---GKGLFTEELENQLLSG----ELDIAVHSSKDMPTVLPEGLHLSAFLPR 103

THAPS LDKSLMELG---GKGLFTKELDTALLGD----EVDICVHSMKDVPTWLPEGTVLPCNLPR 100

HELPY LDTPLNKIG---GKGLFTKELEELLLKG----AIDLAVHSLKDVPVVFEKGLDLACITKR 98

BACSU VDVTLSKVG---GKGLFVKEIEQALLNE----EIDMAVHSMKDMPAVLPEGLVIGCIPER 100

: .:: . :: *** **:* :

ARATH EDVRDAFIC-----------------------LTAATLAELPA----------------- 194

PEA EDVRDAFIS-----------------------LSAASLADLPA----------------- 181

CYAME EDTRDAFVS-----------------------FKAKSLDELPP----------------- 206

CAUCR EDPRDAFIS-----------------------HVCERLEDLPK----------------- 132

TOXO EDPRDALIFSRKPRR----------------GLSRACKAQVPGGVAETARRSATQQRVSA 378

SYNY VNPADALVLNAK--------------------HQGKDLASLPE----------------- 128

SYNC EDPADALVLGAK--------------------WTGHTIDTLPE----------------- 127

HOMO ENPHDAVVFHPK--------------------FVGKTLETLPE----------------- 139

MUS QNPCDAVVFHPK--------------------FIGKTLETLPE----------------- 139

SACCE VDPTDCLVMPFY--------------------SAYKSLDDLPD----------------- 128

SCHPO SCPLDAIVFKAG--------------------SHYKTVADLPP----------------- 128

NEIME ANPFDAFVSN-----------------------QYTRLEEMPE----------------- 121

ESCCO EDPRDAFVSN-----------------------NYDSLDALPA----------------- 128

RICPR EDPRDVLVC-----------------------LNYQSIETLPQ----------------- 120

Wolb LSPYDTFISN-----------------------KYKSLKSLPQ----------------- 118

wAna LSPCDAFISH-----------------------KHNSLESLPQ----------------- 118

wMel LSSCDAFISH-----------------------KHNSLESLPQ----------------- 118

wWill LSSCDAFISH-----------------------KHNSLESLPQ----------------- 128

PLAYO DTINDAFLSIKYKNLRDIN-------------LSSHVIDKEID----------------- 202

PLAFA DTINDAFLSIKYKSINDMNTVKSVSKTEDIHHINKKDSDHNND----------------- 205

Ciliate KPRGDVVIFNEK--------------------HKGKELKDLPE----------------- 164

RHOCA EDTRDAFVSM-----------------------KYNSLAELPE----------------- 132

AGRTU EDMRDAFIGR-----------------------TAPKLLELPQ----------------- 123

THAPS EDTNDAFITANG---------------------DIKRIADLPD----------------- 122

HELPY ADVRDTFLSV-----------------------KFPDLMSLPK----------------- 118

BACSU EDPRDALISK-----------------------NRVKLSEMKK----------------- 120

* .:

ARATH ------------------------------------------------------------

PEA ------------------------------------------------------------

CYAME ------------------------------------------------------------

CAUCR ------------------------------------------------------------

TOXO AASGAPSVGSSASASLLPYLPDALFASGGLPEETEEAGRAARASHTEDDAGGVEGEARKG 438

SYNY ------------------------------------------------------------

SYNC ------------------------------------------------------------

HOMO ------------------------------------------------------------

MUS ------------------------------------------------------------

SACCE ------------------------------------------------------------

SCHPO ------------------------------------------------------------

NEIME ------------------------------------------------------------

ESCCO ------------------------------------------------------------

RICPR ------------------------------------------------------------

Wolb ------------------------------------------------------------

wAna ------------------------------------------------------------

wMel ------------------------------------------------------------

wWill ------------------------------------------------------------

PLAYO ------------------------------------------------------------

PLAFA ------------------------------------------------------------

Ciliate ------------------------------------------------------------

RHOCA ------------------------------------------------------------

AGRTU ------------------------------------------------------------

THAPS ------------------------------------------------------------

HELPY ------------------------------------------------------------

BACSU ------------------------------------------------------------

ARATH ---GSVVGTASLRRK---SQILHKYPALHVEEN-FRGNVQTRLSKLQGG--KVQATLLAL 245

PEA ---GSVIGTASLRRK---SQILHRYPSLTVQDN-FRGNVQTRLRKLSEG--VVKATLLAL 232

CYAME ---GSVVGSSSLRRQ---SQILARYPHLKVIN--FRGNVQTRLRKLEEC--VVDATLLAL 256

CAUCR ---GARLGTASLRRQ---AQALHVRPDLEIVM--LRGNVDTRLAKLERG--EADAILLAQ 182

TOXO DEGRPRIGTCSLRRQ---ALLYALLPPSSFQLMGLRGNVQTRMSRIEKG--TLDATLLAA 493

SYNY ---GAVIGTSSLRRL---AQLRYHFPHLTFKD--VRGNVNTRLAKLDSN--EYDAIILAA 178

SYNC ---GTVIGTSSLRRL---AQLRHYYPHLTFKD--VRGNLNTRLAKLDAG--EYDALILAV 177

HOMO ---KSVVGTSSLRRA---AQLQRKFPHLEFRS--IRGNLNTRLRKLDEQ-QEFSAIILAT 190

MUS ---KSAVGTSSLRRV---AQLQRKFPNLEFKS--IRGNLNTRLRKLDEL-QEFSAIVLAV 190

SACCE ---GGIVGTSSVRRS---AQLKRKYPHLKFES--VRGNIQTRLQKLDDPKSPYQCIILAS 180

SCHPO ---GSVVGTSSIRRR---ALLARNFPHLRFVD--IRGNVGTRLAKLDAPDSQFDCLVLAA 180

NEIME ---GAVVGTSSLRRE---AQLRARYPHLLIKP--LRGNVQTRLSKLDNG--EYDAIILAA 171

ESCCO ---GSIVGTSSLRRQ---CQLAERRPDLIIRS--LRGNVGTRLSKLDNG--EYDAIILAV 178

RICPR ---NAVIGSSAVRRK---AFIKKIRPDLNIKV--FRGNVDSRIKKLMTG--EVDAIILSY 170

Wolb ---QAIIATSSIRRK---VQLLNFRPDLNIVP--LRGNVTTRLQNQS-----FDGIILAE 165

wAna ---QATIATSAIRRK---VQLLNFRPDLNIVP--LRGNVTTRLQNQS-----FDGIILAE 165

wMel ---QATIATSSIRRK---VQLLNVRPDLNIVP--LRGNVTTRLQNQS-----FDGIILAE 165

wWill ---QATIATSSIRRK---VQLLNVRPDLNIVP--LRGNVTTRLQNQS-----FDGIILAE 175

PLAYO --LPRTIATSSLRRT---SQIRYKYKNLKLKF--IRGNINTRIAKLFNN--SFDSIIIAF 253

PLAFA --TLCTIGTSSLRRR---SQIKNRYKNIYVNN--IRGNINTRIEKLYNG--EVDALIIAM 256

Ciliate ---GSVIGTSSLRRI---TNLKNRYPHLKYEN--IRGNLNTRLTKLENG--QYDAIILAE 214

RHOCA ---GAVVGTSSLRRR---AQLAARRPDLKMVE--FRGNVQTRMKKLGEG--VADATFLAL 182

AGRTU ---GAVVGSASLRRQ---ALIRRLRPDLNVIV--FRGLVDTRLRKLEEG--QADATLLAF 173

THAPS ---NSVIGTASLRRQ---AQILAQNPTLKCVN--FRGNVQTRLRKLDDG--VVDATLLAI 172

HELPY ---GAKVGTTSLRRS---MQIKLKRQDLDTES--LRGNVQTRLKKLECG--EFDAIILAE 168

BACSU ---GAVIGTSSLRRS---AQLLIERPDLTIKW--IRGNIDTRLQKLETE--DYDAIILAA 170

:.: ::** .** : :*: . .::

ARATH AGLKRLSMTENVAS--------------------------------ILSLDEMLPAVAQG 273

PEA AGLKRLNMTENVTS--------------------------------TLSIDDMLPAVAQG 260

CYAME AGLRRLQMEHVATA--------------------------------VLGMEDMLPAVAQG 284

CAUCR SGLNRLGLGHLTRS--------------------------------WLDPDACPPAPGQG 210

TOXO AGLKRLNLLSAMLAPSPSATPSPCPAPSPNETSRGTEPGGCVE-AVLLPADVFVPAVCQG 552

SYNY AGLERLDMANRIDQ--------------------------------LIPPEISLHAVGQG 206

SYNC AGLRRLGFGDRISQ--------------------------------VLPATVSLYAVGQG 205

HOMO AGLQRMGWHNRVGQ--------------------------------ILHPEECMYAVGQG 218

MUS AGLQRMGWQNRVGQ--------------------------------ILHPEECMYAVGQG 218

SACCE AGLMRMGLENRITQ--------------------------------RFHSDTMYHAVGQG 208

SCHPO AGLFRLGLKDRIAQ--------------------------------MLTAPFVYYAVGQG 208

NEIME AGLQRLKLDGRIRM--------------------------------ILSESDSLPAAGQG 199

ESCCO AGLKRLGLESRIRA--------------------------------GLPPEISLPAVGQG 206

RICPR AGLKRLNVFNQKYCH-------------------------------LIEYSKMLPCIGQG 199

Wolb AGLIRLKKDYLITE--------------------------------VLSPKTMLSAVGQG 193

wAna AGLIRLEKHHLITE--------------------------------VLPPKVMLSAVGQG 193

wMel AGLIRLEKHHLITE--------------------------------VLPPKVMLSAVGQG 193

wWill AGLIRLEKHHLITE--------------------------------VLPPKVMLSAVGQG 203

PLAYO CGLERLVSKKILRQIMKNNIKD---KSYIINYKN-ISIDLRHLNIQKLNTNIMCPALCQG 309

PLAFA CGIERLIKKANLKHLLKNKEQKNICQPFLLKCNNKKCIDLCHVNIQKLNKNLIYPALGQG 316

Ciliate AGVFRLGWIEKVGQ--------------------------------FLKQEDFLYAPGQG 242

RHOCA AGLNRLGMS--------------------------------EVAKSAIEPEDMLPAVAQG 210

AGRTU AGLKRLGKD--------------------------------NVPTEILDPKEFPPAPAQG 201

THAPS AGLKRMDMD--------------------------------DCATAILEWDEMLPAVAQG 200

HELPY AGLCRLEIQG-------------------------------AKYRKAFSVEEMIPSMGQG 197

BACSU AGLSRMGWKQDVVT-------------------------------EFLEPERCLPAVGQG 199

.*: *: : . **

ARATH AIGIACRTDDDKMATYLASLNHEETRLAISCERAFLETLDG---SCRTPIAGYASK---- 326

PEA AIGIACRSNDDKMAEYLASLNHEETRLAISCERAFLTTLDG---SCRTPIAGYASR---- 313

CYAME AIGITTRRGDDRTAALLGPLSCPRTKLCVEAERAFLANLDG---SCRTPIAGQAWF---- 337

CAUCR ALVIETR-AEDIGAPWLEAVRCRQTTIAVAAERGALLALEG---SCRTAIGARAIL---- 262

TOXO IIGVQCRSADTGILSLLRELNSPSSRGQALCERAFLRALDG---SCRTPIAGIAEWRAVP 609

SYNY ALGIECREGDQEILSLLKVLEDENSRDRCLAERAFLRQLEG---GCQVPIGVNT------ 257

SYNC ALGIECRAEDADILALVKTLEHPETTARCLAERAFLRQLEG---GCQVPIGVHT------ 256

HOMO ALGVEVRAKDQDILDLVGVLHDPETLLRCIAERAFLRHLEG---GCSVPVAVHT------ 269

MUS ALAVEVRAKDQDILDLVSVLHDPETLLRCIAERAFLRHLEG---GCSVPVAVHT------ 269

SACCE ALGIEIRKGDTKMMKILDEICDLNATICCLSERALMRTLEG---GCSVPIGVES------ 259

SCHPO ALAVEVRADDKEMIEMLKPLQHQETLYACLAERALMKRLQG---GCAIPIGVQTDV---- 261

NEIME ALGIEIAAHREDLYEVLKPLNHGVTNACVTAERALARALGG---SCQVPLAAYC------ 250

ESCCO AVGIECRLDDSRTRELLAALNHHETALRVTAERAMNTRLEG---GCQVPIGSYA------ 257

RICPR VIAVEIRKDDNAMFNICSQINHLPTFELIKPERAFLEYLDA---NCSTPIAAYAQY---- 252

Wolb AICIQCRKNDIKIIDLLEKINNNKSFIRVKSERSFMKTVNG---SCFTPLAALAKY---- 246

wAna AICIQCRRNDVKIIDLLEKINNNMSFIGVKSERSFMKTVNG---SCFTPLAALAEY---- 246

wMel AVCIQCRRNDVKIIDLLEKINNNMSFIGVKSERSFMKTVNG---SCFTPLAALAEY---- 246

wWill AVCIQCRRNDVKIIDLLEKINNNMSFIGVKSERSFMKTVNG---SCFTPLAALAEY---- 256

PLAYO IIGVTSNKNNPEISQILKNINNEKSQIMANIERAFLQKIDG---SCTMPIGGYT------ 360

PLAFA IIAVTSHKKNYFISSLLKNINNKKSEMMAQIERSFLYHIDG---NCMMPIGGYT------ 367

Ciliate ALGVQCRADDKEAIALLSYINQKEPRIRVDAERQFLNKLEGVILGCKLPIAVHS------ 296

RHOCA CIGIERREADTRAKMLLDAIHHAPSGLRLACERSFLLTLDG---SCETPIGGLS------ 261

AGRTU AIGVESRIGDARMDKLLAPINDRPTYDAVTCERAFLAALDG---SCRTPIAGYA------ 252

THAPS AIGIQCRSDDTRSLKYIDALNCMDTHVCVNCERAFLEALDG---NCKTPIAGQA------ 251

HELPY ALGVEMLKN-HKHFATLQKLNDEKSAFCCRLEREFIKGLNG---GCQIPIGVHA------ 247

BACSU ALAIECRESDEELLALFSQFTDEYTKRTVLAERAFLNAMEG---GCQVPIAGYS------ 250

: : . . ** : . .* .:.

ARATH ------------------------------------------------------------

PEA ------------------------------------------------------------

CYAME ------------------------------------------------------------

CAUCR ------------------------------------------------------------

TOXO AQGEAPRPQSQNGSDEPGVEREGEEGCAGSSVKGREGRSEEGWGGHPDCFTEKTELRFCG 669

SYNY ------------------------------------------------------------

SYNC ------------------------------------------------------------

HOMO ------------------------------------------------------------

MUS ------------------------------------------------------------

SACCE ------------------------------------------------------------

SCHPO ------------------------------------------------------------

NEIME ------------------------------------------------------------

ESCCO ------------------------------------------------------------

RICPR ------------------------------------------------------------

Wolb ------------------------------------------------------------

wAna ------------------------------------------------------------

wMel ------------------------------------------------------------

wWill ------------------------------------------------------------

PLAYO ------------------------------------------------------------

PLAFA ------------------------------------------------------------

Ciliate ------------------------------------------------------------

RHOCA ------------------------------------------------------------

AGRTU ------------------------------------------------------------

THAPS ------------------------------------------------------------

HELPY ------------------------------------------------------------

BACSU ------------------------------------------------------------

ARATH ---DEEGN--CIFRGLVASPDGTKVLE--TSRKGPYVYED-------------------- 359

PEA ---DKDGN--CLFRGLVASPDGTRVLE--TSRIGSYTYED-------------------- 346

CYAME ---DTDSSERIHFRGLVATPDGRDLFE--TTR--QCTPAN-------------------- 370

CAUCR ------DGARLSMIVEALTPDGAQRFR--REGDITLTGAD-------------------- 294

TOXO VLATPDGKQLLRFCGVLATPDGKQLFREERTVRGVRTPEE-------------------- 709

SYNY ----HLDGDNLTLTGMVASLDGQRLIKDTLSAPRKE------------------------ 289

SYNC ----VIENGQLTLTGLVASLDGQRLVKDSLTGDVAT------------------------ 288

HOMO ----AMKDGQLYLTGGVWSLDGSDSIQETMQATIHVPAQHEDGPEDDPQLVGITARNIPR 325

MUS ----VIKDGQLYLTGGVWSLDGSDSMQETMQATIQVPVQQEDGPEDDPQLVGITARNIPR 325

SACCE --KYNEETKKLLLKAIVVDVEGTEAVEDEIEMLIEN------------------------ 293

SCHPO -LAISNSSYRISLLGTVLSADGLRAAFGNAEAVVS------------------------- 295

NEIME ----TEENGLLTLRGLVGHPDGSVVLRADAQAPAEY------------------------ 282

ESCCO ----ELIDGEIWLRALVGRPDGSQIIRGERRGAPQD------------------------ 289

RICPR -----LDAYNIQIDFMLGNLDCTKIIFQTEITNIKT------------------------ 283

Wolb -------VSENVLHLYCMLADKKNIYFTERTSFVED------------------------ 275

wAna -----VSENMLYLSLYVGRWKKYILH---------------------------------- 267

wMel -----VSENMLHLRCMLAS--GKNIYFTERTSFIED------------------------ 275

wWill -----VSENMLHLRCMLAS--GKNIYFTERTSFIED------------------------ 285

PLAYO ----KFSKNKIFFNAIINDINGLENYKIKVVRHLND------------------------ 392

PLAFA ----NMRNEDIYLHVIINDIHGYNKYQVTQKDTLYN------------------------ 399

Ciliate ----EIIDQTLRLTGQVWSLEADKTIREVIEGHIS------------------------- 327

RHOCA ----VLEGDQIWLRGEILRPDGSETITGEIRG---PAAD--------------------- 293

AGRTU ----TCEGDNLHFSGLILTPDGQTSHGVEISG---NRRD--------------------- 284

THAPS ----RIVDGKIVFKGLIAMPDGSLKYETEATG---EIAD--------------------- 283

HELPY ----SLMGDRVKIQAVLGLPNGKEVITKEKQG---DKTK--------------------- 279

BACSU ---VLNGQDEIEMTGLVASPDGKIIFKETVTGNDP------------------------- 282

:

ARATH -----MVKMGKDAGQELLSRA--------------------------GPGFFGN------ 382

PEA -----MMKIGKDAGEELLSRA--------------------------GPGFFNS------ 369

CYAME -----VIDECAAAGTELRDRV--------------------------GPDFYASLVNYVQ 399

CAUCR -----DIAEARAFGLTLGAEV--------------------------RAAGGDAIILPE- 322

TOXO -----AEALGRAVAEEIKKAA--------------------------GADRLAQIKKHVT 738

SYNY -----AEKLGQDLALKLREQG--------------------------AGEILAEILAEAG 318

SYNC -----AEELGTRLALKLREQG--------------------------ASEILEEIFATVR 317

HOMO GPQLAAQNLGISLANLLLSKG--------------------------AKNILDVARQLND 359

MUS GAQLAAENLGISLASLLLNKG--------------------------AKNILDVARQLND 359

SACCE -VKEDSMACGKILAERMIADG--------------------------AKKILDEINLDRI 326

SCHPO -SEEEAEELGITVALALLKNG--------------------------AGPILEEHQRSSD 328

NEIME -----ADALGRAVAKKLADDG--------------------------ARELIGAVLNTEN 311

ESCCO -----AEQMGISLAEELLNNG--------------------------AREILAEVYNGDA 318

RICPR -----SKICGIKAAKMMLAQQ--------------------------------------- 299

Wolb -----AEKMGMDAGLELKSKCL-------------------------------------- 292

wAna ------------------------------------------------------------

wMel -----AEKMGMDAGLELKSKCL-------------------------------------- 292

wWill -----AEKMGMDAGLELKSKCL-------------------------------------- 302

PLAYO -----LDGIADEAAEKIKEKIGIDHRCSFILQIINMKEHQWRLFEYNMSKWMIENKYILD 447

PLAFA -----YKEIGPNAAIKMKEIIGTEQ----------------------FNKIKAEAELHLL 432

Ciliate -----DQDLGLKLSEKMKQAG--------------------------AGEILEKIRSQID 356

RHOCA -----GVALGAELAKQLLAKA--------------------------PADFFCWR----- 317

AGRTU -----ALILGKKAGEEVRAKA--------------------------GSNFFEGWS---- 309

THAPS -----AVEIGRKAGEELKAQA--------------------------GEKFFE------- 305

HELPY -----AFDLVQELLEEFLQSG--------------------------AKEILEKAQLF-- 306

BACSU ------EEVGKRCAALMADKG--------------------------AKDLIDRVKRELD 310

ARATH ------------------------------------------------------------

PEA ------------------------------------------------------------

CYAME ERDRAEPGRQTKSKRSTS------------------------------------------ 417

CAUCR ------------------------------------------------------------

TOXO EGWKNLKTL--------------------------------------------------- 747

SYNY RG---------------------------------------------------------- 320

SYNC PER--------------------------------------------------------- 320

HOMO AH---------------------------------------------------------- 361

MUS VR---------------------------------------------------------- 361

SACCE K----------------------------------------------------------- 327

SCHPO SEESLKNY---------------------------------------------------- 336

NEIME ------------------------------------------------------------

ESCCO PA---------------------------------------------------------- 320

RICPR ------------------------------------------------------------

Wolb ------------------------------------------------------------

wAna ------------------------------------------------------------

wMel ------------------------------------------------------------

wWill ------------------------------------------------------------

PLAYO NEEKKKFYKCANYSIGSGMLNASLIYFLCKKNQKYFSSMSRLFLTCSLGIYTSMVVNKIY 507

PLAFA NNK--------------------------------------------------------- 435

Ciliate KKENEE------------------------------------------------------ 362

RHOCA ------------------------------------------------------------

AGRTU ------------------------------------------------------------

THAPS ------------------------------------------------------------

HELPY ------------------------------------------------------------

BACSU EDGK-------------------------------------------------------- 314

ARATH ------------------------------------------------------------

PEA ------------------------------------------------------------

CYAME ------------------------------------------------------------

CAUCR ------------------------------------------------------------

TOXO ------------------------------------------------------------

SYNY ------------------------------------------------------------

SYNC ------------------------------------------------------------

HOMO ------------------------------------------------------------

MUS ------------------------------------------------------------

SACCE ------------------------------------------------------------

SCHPO ------------------------------------------------------------

NEIME ------------------------------------------------------------

ESCCO ------------------------------------------------------------

RICPR ------------------------------------------------------------

Wolb ------------------------------------------------------------

wAna ------------------------------------------------------------

wMel ------------------------------------------------------------

wWill ------------------------------------------------------------

PLAYO RRKAYTEILTEKTTMTDKAIEVMNDILNVNNNKIPTNETNNLTNKINEYNARDGSDIKTN 567

PLAFA ------------------------------------------------------------

Ciliate ------------------------------------------------------------

RHOCA ------------------------------------------------------------

AGRTU ------------------------------------------------------------

THAPS ------------------------------------------------------------

HELPY ------------------------------------------------------------

BACSU ------------------------------------------------------------

ARATH ----------------

PEA ----------------

CYAME ----------------

CAUCR ----------------

TOXO ----------------

SYNY ----------------

SYNC ----------------

HOMO ----------------

MUS ----------------

SACCE ----------------

SCHPO ----------------

NEIME ----------------

ESCCO ----------------

RICPR ----------------

Wolb ----------------

wAna ----------------

wMel ----------------

wWill ----------------

PLAYO SDNNRNEHTENLVQNL 583

PLAFA ----------------

Ciliate ----------------

RHOCA ----------------

AGRTU ----------------

THAPS ----------------

HELPY ----------------

BACSU ----------------

**CLUSTAL X (1.83) multiple sequence alignment for UROS (4)**

**18 taxa/157 characters from underlined conserved regions are used for phylogeny analysis**

**Legend for abbreviations used in the alignment:**

**TOXO** 1275 aa DQ029342 uroporphyrinogen III synthase Apicomplexa/Alveolates

**CYAME*** 299aa CML040C uroporphyrinogen III synthase retrieved from http://merolae.biol.s.u-tokyo.ac.jp/ Cyanidioschyzon merolae Genome Project, Red algae

**THAPS** EED94993 330aa incomplete uroporphyrinogen III synthase genewise.159.2.1 retrieved from http://genome.jgi-psf.org/thaps1/thaps1.home.html thalassiosira pseudonana Diatoms/Heterokonts

**Arath** CAC85287 uroporphyrinogen III synthase [Arabidopsis thaliana] Green plants

**SyneC** 264aa CAA50303 uroporphyrinogen-III synthase [Synechococcus sp. PCC 7942] Cyanobacteria

**SYNE** 538aa NP_441993 uroporphyrinogen-III synthase/methyltransferase [Synechocystis sp. PCC 6803] Cyanobacteria

**RICP** 269aa NP_220853 uroporphyrinogen-III synthase [Rickettsia prowazekii str. Madrid E] alpha-proteobacteria

**AGRO** 246aa NP_355590 uroporphyrinogen-III synthase [Agrobacterium tumefaciens str. C58] alpha-proteobacteria

**Wolb** AAW71316 235aa Uroporphyrinogen-III synthase [Wolbachia endosymbiont strain TRS of Brugia malayi] alpha-proteobacteria

**wSim** ZP_00372506 235aa Uroporphyrinogen-III synthase [Wolbachia endosymbiont of Drosophila simulans] alpha-proteobacteria

**wMel** NP_966727 235aa Uroporphyrinogen-III synthase [Wolbachia endosymbiont of Drosophila melanogaster] alpha-proteobacteria

**NeiME** NP_273819 246aa uroporphyrinogen-III synthase HemD, [Neisseria meningitidis MC58] Beta-proteobacteria

**ESCCO** NP_418248 246aa uroporphyrinogen III synthase [Escherichia coli K12] Gamma-proteobacteria

**SACC** NP_014921 275aa Uroporphyrinogen III synthase, [Saccharomyces cerevisiae] Fungi

**SCHPO** NP_594008 251aa uroporphyrinogen-iii synthase [Schizosaccharomyces pombe] Fungi

**MUS** A56838 265aa uroporphyrinogen-III synthase (EC 4.2.1.75) - mouse [Mus musculus] Mammals

**HOMO** AF230665 265aa uroporphyrinogen III synthase [Homo sapiens] Mammals

**BACSU** B42728 262aa uroporphyrinogen-III synthase (EC 4.2.1.75) hemD - Bacillus subtilis Firmicutes/G+ bacteria

MUS ------------------------------------------------------------

HOMO ------------------------------------------------------------

SACC ------------------------------------------------------------

SCHPO ------------------------------------------------------------

SYNC ------------------------------------------------------------

SYNE MAEKLPHPQCLGVPGRSPRRESNLIFTMLSTDSRAKVYLMGAGLGPVAYLTQRAIAVLGR 60

TOXO ----------------------------------------------------------MA 2

Arath ------------------------------------------------------------

CYAME ------------------------------------------------------------

THAPS ------------------------------------------------------------

ESCCO ------------------------------------------------------------

NeiME ------------------------------------------------------------

RICP ------------------------------------------------------------

Wolb ------------------------------------------------------------

wMel ------------------------------------------------------------

wSim ------------------------------------------------------------

AGRO ------------------------------------------------------------

BACSU ------------------------------------------------------------

MUS ------------------------------------------------------------

HOMO ------------------------------------------------------------

SACC ------------------------------------------------------------

SCHPO ------------------------------------------------------------

SYNC ------------------------------------------------------------

SYNE ANVVIYDALVSQELFDLLPPDCERIFVGKRGGQPSTPQAKINQLLVDHYRRGKQVVRLKS 120

TOXO ACPLLPLFLVDRVLCFFSFICSSSLLPRRSRAVSAYASFALLLFSFPAFIAPHAGIFSVR 62

Arath ------------------------------------------------------------

CYAME ------------------------------------------------------------

THAPS ------------------------------------------------------------

ESCCO ------------------------------------------------------------

NeiME ------------------------------------------------------------

RICP ------------------------------------------------------------

Wolb ------------------------------------------------------------

wMel ------------------------------------------------------------

wSim ------------------------------------------------------------

AGRO ------------------------------------------------------------

BACSU ------------------------------------------------------------

MUS ------------------------------------------------------------

HOMO ------------------------------------------------------------

SACC ------------------------------------------------------------

SCHPO ------------------------------------------------------------

SYNC ------------------------------------------------------------

SYNE GDPWIFGRIMPELATLVTHHCAYEVVPGISSAIAGAGLASIPLTAKDSGAGFFVMDGHDP 180

TOXO ASTLSVDAFFPSPAHLSQGQVGFSGRPVLFLNKEKTSKTLFPSSIAPHLPPPLVPSAASL 122

Arath ------------------------------------------------------------

CYAME ------------------------------------------------------------

THAPS ------------------------------------------------------------

ESCCO ------------------------------------------------------------

NeiME ------------------------------------------------------------

RICP ------------------------------------------------------------

Wolb ------------------------------------------------------------

wMel ------------------------------------------------------------

wSim ------------------------------------------------------------

AGRO ------------------------------------------------------------

BACSU ------------------------------------------------------------

MUS ------------------------------------------------------------

HOMO ------------------------------------------------------------

SACC ------------------------------------------------------------

SCHPO ------------------------------------------------------------

SYNC ------------------------------------------------------------

SYNE HRWPWP-ALAQLPTLVILMGTKNLSLLVNELVRAGKSPHTPMAVIKNAGRPEQQTWEG-- 237

TOXO SGPLRSRCHFTAPSSASLSFVSPSLSAFLSFASCFDRCAFPLHPRPRLHLFPAPAWTSSL 182

Arath ------------------------------------MALLLLSHCSILSFQPPLSSSSSF 24

CYAME ------------------------------------------------------------

THAPS ------------------------------------------------------------

ESCCO ------------------------------------------------------------

NeiME ------------------------------------------------------------

RICP ------------------------------------------------------------

Wolb ------------------------------------------------------------

wMel ------------------------------------------------------------

wSim ------------------------------------------------------------

AGRO ------------------------------------------------------------

BACSU ------------------------------------------------------------

MUS ------------------------------------------------MKVLLLKDAKED 12

HOMO ------------------------------------------------MKVLLLKDAKED 12

SACC ------------------------------------------MSSRKKVRVLLLKNKTVP 18

SCHPO -----------------------------------------------MKTALLLKTKSQP 13

SYNC -----------------------------------------MAEQPLIGKTILTTRAAGQ 19

SYNE ---------TLADMVEKTQGQSLAPAVIVIGDVVSQRIIPALPPLPLTEKTILVTRAADQ 288

TOXO RHSRFHGWEKPLLSPSYVPAHAVPVPVRMRFSFRFSSAQPSVSHRSSFPSSLYSSHSVSP 242

Arath HSSHVQSLSKPVFAS--------PSPIRNSISSSVSSSSSSVSSSN-SIPQVVVTRERGK 75

CYAME -----MFLWASSPCLVIGRGAFPLGKSRSLTCGANRRRVSSLGSIDSLGATLGIALTRER 55

THAPS ----------------------RNSKSPAEVDVSVGSTTSSSPFVVALTREEGKNDKLRK 38

ESCCO -------------------------------------------------MSILVTRPSPA 11

NeiME ---------------------------------------------------MLIVRPSGR 9

RICP ------------------------------------------------MKSVLLTRNIQE 12

Wolb ------------------------------------------------MKSILLTRPLSD 12

wMel ------------------------------------------------MKSILLTRPLLD 12

wSim ------------------------------------------------MKSILLTRPLLD 12

AGRO -------------------------------------------------MRIVVTRPQRS 11

BACSU ----------------------------------------MENDFPLKGKTVLVTRNKAQ 20

MUS DSGLDPYIQELRLCGLEATLIPVLSFEFMSLPS--LSEKLSHP-EGFGGLIFTSPRAVEA 69

HOMO DCGQDPYIRELGLYGLEATLIPVLSFEFLSLPS--FSEKLSHP-EDYGGLIFTSPRAVEA 69

SACC ---IDKYELECRSKAFEPIFVPLIKHTHVIQDFRNVLNTIPNYLNTINYIIITSQRTVES 75

SCHPO ---FDPYVEAFEKYGRDTAFIPVLRHKRVHEEQ--LRDKLKNVRKTYCGLIVTSQRVSET 68

SYNC ---SSPFAAQLRAAGAAVIEMPTLEIGPPSSWLPLDEAIAAIAD--FDWLILASANAVEA 74

SYNE ---ASQFTELLHQQGATVLAMAALEIVPPKDWQPLDQAIAKLGD--FDWLILTSANGVEF 343

TOXO ---PESAASASCSLSSSSVSAPPLPTALCPAWPRSASRPAAFFS--FDYLCLTSPESARV 297

Arath ---NNQIIKALEKNGISSLELPLIQHARG---PDFDRLASVLNDKSFDWIIITSPEAGSV 129

CYAME GKNSKLATLVRRRLPEARIYELPCIAHKVRTDADRIVQLLASDADSIDWILITSPTAAEV 115

THAPS ALLSNEHIQQLSQSSGVAVDIHEIPCIEHADGPDTDKLVPTLASTQFDYVAITSPEAAKV 98

ESCCO ---GEELVSRLRTLGQVAWHFPLIEFSPGQQLPQLADQLAALGE--SDLLFALSQHAVAF 66

NeiME ---AAEDVETCLNAGWRAEVLSPVEIEA----DAAGLELLSEQYARADAVFWVSPTAVET 62

RICP ---NNETIQEINKYNLDLRYIHCSLIKYKT------LDFNINILNNYSNIIITSKYAAHI 63

Wolb ---SLNTRSTLKKYGYKVYIEPAFTIKHLQP---------DISAYKFDVVISTSKNSVKA 60

wMel ---SFNTRNILRKYGYKVYIEPVFTIKYLNP---------DISAHEFDVVISTSKNSVKA 60

wSim ---SFNTRNILRKYGYKVYIEPVFTIKYLNP---------DISAYEFDVVISTSKNSVKA 60

AGRO ---GERTAAKLEALGHAPVLLPLFHPIHHG------ERATSALSDPAGAIAVTSAEALRA 62

BACSU ---AASFQQKVEALGGKAVLTSLITFRRALPNDVAEQVREDLAAP--GWLVFTSVNGADF 75

: *

MUS VKLCLEKDNK------------------------------------------TEAWEKSL 87

HOMO AELCLEQNNK------------------------------------------TEVWERSL 87

SACC LNEAII-PTL----------------------------------------------TSEQ 88

SCHPO LDEALK-QED----------------------------------------------ETER 81

SYNC VQQRLAAQ--------------------------------------------------QK 84

SYNE FFQRLQHHG-------------------------------------------------LD 354

TOXO FARCWERMLTCLLPIASQSCSSTSSSTSLTPPSYPSHSPPSCESSSCSAASVSPPHLFRC 357

Arath FLEAWK------------------------------------------------------ 135

CYAME FAQVVRSAQE-------------------------------------------------- 125

THAPS LVNAWIEAGR-------------------------------------------------- 108

ESCCO AQSQLHQQ---------------------------------------------------- 74

NeiME AVPYL------------------------------------------------------- 67

RICP LADYNLKQDIWVVG---------------------------------------------N 78

Wolb FSQICR------------------------------------------------------ 66

wMel FSQICK------------------------------------------------------ 66

wSim FSQICK------------------------------------------------------ 66

AGRO LATCEEQLAPY------------------------------------------------- 73

BACSU FFSYLKE----------------------------------------------------N 83

MUS KDRWNAK-SVYVVGSATASLVNKIGLD--------------------------------- 113

HOMO KEKWNAK-SVYVVGNATASLVSKIGLD--------------------------------- 113

SACC KAALLSK-TVYTVGPATANFIRRSGFINV------------------------------- 116

SCHPO QKILMET-PIFTVGPATDDSIRRLGFQQ-------------------------------- 108

SYNC S-WSDVPCAIAVVGQKTAQVLAAQGGKAD------------------------------- 112

SYNE SRALAGL-KLAVVGKKTAQSLEKFGLKPD------------------------------- 382

TOXO LDSVPRQ-SIVCVGDGTRHAAEEALHAVFKRLCRHRGNADDTATNEARRGPLEGKNNARE 416

Arath TASSPEV-QIGVVGAGTARVFEEAMKSADG------------------------------ 164

CYAME -RGLQRLPPIASVGAATNTALLKYSLQAN------------------------------- 153

THAPS ----PQLGKVAAVGKATQESLKEFGIEVA------------------------------- 133

ESCCO DRKWPRLPDYFAIGRTTALALHTVSGQ--------------------------------- 101

NeiME -NLSDGIKAQIAVGQGSRRALERCLVR--------------------------------- 93

RICP KTKQLLGKKVIYTANNIADLIQHFPTD--------------------------------- 105

Wolb ----VDDLPIITVGNSTMQTAKDSGFS--------------------------------- 89

wMel ----VDDFPIITVGNSTMQAAKNLGFS--------------------------------- 89

wSim ----EDDFPIITVGNSTMQAAKNLGFS--------------------------------- 89

AGRO -----LSKPLFAVGGATAEAAQETGFE--------------------------------- 95

BACSU QLILPAHKKIAAVGEKTARRLKMHNVS--------------------------------- 110

.

MUS ------------------------------------------------------------

HOMO ------------------------------------------------------------

SACC ------------------------------------------------------------

SCHPO ------------------------------------------------------------

SYNC ------------------------------------------------------------

SYNE ------------------------------------------------------------

TOXO SEEETSQNVEGDDRVSGTASQRHGFRGHKLDLNAEETATSERSTNDSRRREGRELDCNFQ 476

Arath ------------------------------------------------------------

CYAME ------------------------------------------------------------

THAPS ------------------------------------------------------------

ESCCO ------------------------------------------------------------

NeiME ------------------------------------------------------------

RICP ------------------------------------------------------------

Wolb ------------------------------------------------------------

wMel ------------------------------------------------------------

wSim ------------------------------------------------------------

AGRO ------------------------------------------------------------

BACSU ------------------------------------------------------------

MUS -----------AEGAGSG---NAEKLAEYICSKPSSELP--------------------- 138

HOMO -----------TEGETCG---NAEKLAEYICSRESSALP--------------------- 138

SACC -----------KGGEDAG---NGSILADIIIDDLSTDIKACPPSE--------------- 147

SCHPO -----------THGKDCG---RGEVLAD-LIEEWYTTTKQHKP----------------- 136

SYNC -------------------YIPPEFIAESLVEHFPQPVAG-------------------- 133

SYNE -------------------FIPPDFIADALVEHFPHSPAG-------------------- 403

TOXO NVSDETQSASREIQSLFSPWMPSVATAARLAADLPPRPPILSLLCLTSDNDYRGGFSPDA 536

Arath -----------LLHVA---FTPSKATGKVLASELP-EKVGKR------------------ 191

CYAME -------------------FIPSRAIGACLGDELPVEKLPKVR----------------- 177

THAPS -------------------FVPSKATAATLVKELPLSDAATKEGR--------------- 159

ESCCO -----------KILYP-----QDREISEVLLQLPELQNIAGKR----------------- 128

NeiME -----------TVIAPDDGNDSEAVLRLPVWNSLPEGAR--------------------- 121

RICP -------------LYKHTIYLSSNEITQDLPNKIARHIIYNVEY---------------- 136

Wolb ------------------DIISADSNVEGLISFVKNYYSRAVK----------------- 114

wMel ------------------DIISADSNVDGLISFIKAHYSSAIK----------------- 114

wSim ------------------DIISADSNVDGLISFIKAHYSNAIK----------------- 114

AGRO ----------------QIFTASGDALGLTVLVKQHR------------------------ 115

BACSU -----------------VDVMPQEYIAEQLADALKQHAEPG------------------- 134

:

MUS ---------------------------------------------------------LLF 141

HOMO ---------------------------------------------------------LLF 141

SACC ---------------------------------------------------------LLF 150

SCHPO ---------------------------------------------------------LLF 139

SYNC -------------------------------------------------------QRLLF 138

SYNE -------------------------------------------------------LKILF 408

TOXO FNERCIHAPEQHTDPLEDVGHVDPWCNYTRAQRVAVSVELPGLRRSVVCCPCWLQTRVVW 596

Arath -------------------------------------------------------SSVLY 196

CYAME ---------------------------------------------------------ILY 180

THAPS ------------------------------------------------------SSTLFY 165

ESCCO ---------------------------------------------------------ALI 131

NeiME ---------------------------------------------------------VLF 124

RICP -------------------------------------------LNELPISIIQEFENIRY 153

Wolb ---------------------------------------------------------FLY 117

wMel ---------------------------------------------------------FLY 117

wSim ---------------------------------------------------------FLY 117

AGRO -------------------------------------------------AFLTEEEPLLY 126

BACSU -------------------------------------------------------ETITV 139

MUS PCG-TIKGDTLPKMLKDKG----------------------------------------- 159

HOMO PCG-NLKREILPKALKDKG----------------------------------------- 159

SACC LVG-EIRRDIIPKKLHSKG----------------------------------------- 168

SCHPO LVG-EKHRDIIQRKLGDDR----------------------------------------- 157

SYNC PRVETGGREQITQALQSQG----------------------------------------- 157

SYNE PRVESGGRAQLVQELSQKQ----------------------------------------- 427

TOXO PTS-ALASEDLATTFEERKLREVVQLFGAQNVSVSSAPRSCDQPSSGDSPGDSVYIHPNK 655

Arath PAS-LKAGNDIVEGLSKRG----------------------------------------- 214

CYAME PTS-ASASDEIEKRLRSRV----------------------------------------- 198

THAPS PAS-NKAADTLQNGLEERG----------------------------------------- 183

ESCCO LRG-NGGRELIGDTLTARG----------------------------------------- 149

NeiME VRG-HGGRDFLMNALQEKG----------------------------------------- 142

RICP FSK-PAYRNAFKANTIRAT----------------------------------------- 171

Wolb IRG-QEVSCDLKKRLSEEG----------------------------------------- 135

wMel IRG-QEVSCDLKKRLSEED----------------------------------------- 135

wSim IRG-QEVSCDLKKRLSEED----------------------------------------- 135

AGRO LAG-RPRGSVFEEGLAAAG----------------------------------------- 144

BACSU MKG-NLSRDVIKQELVPLG----------------------------------------- 157

:

MUS ------------------------------------------------------------

HOMO ------------------------------------------------------------

SACC ------------------------------------------------------------

SCHPO ------------------------------------------------------------

SYNC ------------------------------------------------------------

SYNE ------------------------------------------------------------

TOXO SSCGDVQKASFHGTVLPSVSSRTRVSLSDHNDVAFSARTPEEIHPKTRDAPEHGRKGQTA 715

Arath ------------------------------------------------------------

CYAME ------------------------------------------------------------

THAPS ------------------------------------------------------------

ESCCO ------------------------------------------------------------

NeiME ------------------------------------------------------------

RICP ------------------------------------------------------------

Wolb ------------------------------------------------------------

wMel ------------------------------------------------------------

wSim ------------------------------------------------------------

AGRO ------------------------------------------------------------

BACSU ------------------------------------------------------------

MUS ------------------------------IPMESMHVYQTVPHP-GIQGS-------LK 181

HOMO ------------------------------IAMESITVYQTVAHP-GIQGN-------LN 181

SACC ------------------------------IKVREVVTYKTEELSDGFKRF-------IH 191

SCHPO --------------------------------VDSLIVYATQELE-NTETQ-------IK 177

SYNC ------------------------------AIVVEVPAYESRCPS-QIPDD-------AL 179

SYNE ------------------------------ALVTEVPSYQSACPG-HMPEE-------VW 449

TOXO ARAVNDVFGGTEAREKEARSETASWTSLPLFRLQRLNIYTTTQRMLALEERQALLATLRE 775

Arath ------------------------------FEVVRLNTYTTVPVQ-----------SVDT 233

CYAME ----------------------------PAVEFIRIDMYDTIEAKWTTEDIDTARNSVD- 229

THAPS ------------------------------FDVMRLNTYDTVPASWDEEQT--------- 204

ESCCO ------------------------------AEVTFCECYQRCAIH--YDGAE-------E 170

NeiME ------------------------------FRTEVAEVYFRRHKPLNFQNF--------Q 164

RICP ------------------------------TAYKKVFNDPSLG---STYPL--------E 190

Wolb ------------------------------FNVREVILYKTIIKR-GLTNR-------CK 157

wMel ------------------------------FNVREVVLYKTIIKR-SLTNR-------CK 157

wSim ------------------------------FNVREVVLYKTIIKR-SLTNR-------CK 157

AGRO ------------------------------IPFRTVDCYEMLPSDISENTL--------E 166

BACSU ------------------------------FEVKEWVLYETIPDEEGIEAL--------K 179

MUS SYYEDQGIP--------------------------------------------------- 190

HOMO SYYSQQGVP--------------------------------------------------- 190

SACC AMKECDEDEVFS------------------------------------------------ 203

SCHPO DTIRKHPTI--------------------------------------------------- 186

SYNC IALRQAHL---------------------------------------------------- 187

SYNE QSIVNQTV---------------------------------------------------- 457

TOXO ALLKREKSAEPADTPRTAAARATVETRKEEKAQASETESGKTEGAGQRQLLEGGRDGIEA 835

Arath VLLQQALSAP-------------------------------------------------- 243

CYAME ------------------------------------------------------------

THAPS -LLAQSAN---------------------------------------------------- 211

ESCCO AMRWQAREVT-------------------------------------------------- 180

NeiME TENIAAAYI--------------------------------------------------- 173

RICP VPLGKMSID--------------------------------------------------- 199

Wolb NLLLGGRIN--------------------------------------------------- 166

wMel NLLLDGKIG--------------------------------------------------- 166

wSim NLLLDGKID--------------------------------------------------- 166

AGRO TTLLDTTAD--------------------------------------------------- 175

BACSU DAAGQYSFD--------------------------------------------------- 188

MUS -------------------ASITFFSPSGLKYSL-------------------------- 205

HOMO -------------------ASITFFSPSGLTYSL-------------------------- 205

SACC -------------------DWVVVFSPQGTKEIT-------------------------- 218

SCHPO -------------------DWIVAFSPTSICSLL-------------------------- 201

SYNC -------------------NLISFTSSKTVRNFC-------------------------- 202

SYNE -------------------DVITFASSKTVSHFR-------------------------- 472

TOXO QTQEGEEKGEDGQDAKVTVAFVMLASPSAVWSWVANGLPLEHSTLDAPVLPSYTAIHAGA 895

Arath --------------------VLSVASPSAVRAWL-------------------------- 257

CYAME --------------------LVTLASPSAVKVWA-------------------------- 243

THAPS --------------------VACFASPSAVKAWL-------------------------- 225

ESCCO --------------------MVVVTSGEMLQQLW-------------------------- 194

NeiME --------------------TSTELVRLLFGQLP-------------------------- 187

RICP --------------------FILLYSQNSAKTLVR------------------------- 214

Wolb --------------------SVAFFSSQTARIFCS------------------------- 181

wMel --------------------SVAFFSSQTARVFCS------------------------- 181

wSim --------------------SVAFFSSQTARVFCS------------------------- 181

AGRO --------------------MVLLYSSEAARAFFH------------------------- 190

BACSU --------------------YVTFTSSSTVHTFM-------------------------- 202

MUS ------------------------------------------------------EYIQAL 211

HOMO ------------------------------------------------------KHIQEL 211

SACC ------------------------------------------------------QYLGDS 224

SCHPO ------------------------------------------------------NTF--- 204

SYNC ------------------------------------------------------QLMASN 208

SYNE ------------------------------------------------------QLLAQT 478

TOXO KQIVVDSLPRKKSSSRSSSSSAAASPASPTCSQAPLELSPSAREGSGQPRKAASHLLSTE 955

Arath ------------------------------------------------------HLIQNE 263

CYAME ------------------------------------------------------QRVG-- 247

THAPS ------------------------------------------------------KNTDGM 231

ESCCO ------------------------------------------------------SLIPQW 200

NeiME ------------------------------------------------------PQFSRF 193

RICP ------------------------------------------------------LLLQNN 220

Wolb ------------------------------------------------------LVLKSG 187

wMel ------------------------------------------------------LVLKSG 187

wSim ------------------------------------------------------LVLKSG 187

AGRO ------------------------------------------------------HVSADK 196

BACSU ------------------------------------------------------HVLGEE 208

MUS SGSS-----------------------FDQIKFIAIGPSTTRAMAAK--GLPVSCTAESP 246

HOMO SGDN-----------------------IDQIKFAAIGPTTARALAAQ--GLPVSCTAESP 246

SACC NRLPG-----------------------SHLRVASIGPTTKKYLDDN--DVTSDVVSPKP 259

SCHPO -----------------------------ELKIATIGPTTGDYLKKL--GTQPNVVSPAP 233

SYNC LGV-------------------DWSARISGVAIASIGPQTSITCQEL--LGRVEVEAQEY 247

SYNE SQANGY---------------QDWRSLINHCQIASIGPQTSERCLRE--LGRVDIEATEY 521

TOXO ADLMRLLEECRNSVMEGREAEKRRRKETQKLVALAIGPTTAAAARHA--GFAKVVAAAQP 1013

Arath EQWSN--------------------------YVACIGETTASAARRL--GLKNVYYPEKP 295

CYAME ----------------------------TKQPAVCIGKTSADAAKEV--GFSEVYAPSDP 277

THAPS NKPAR-------------------------AKAACIGETSAEACRKNQWDESDIFYPEKP 266

ESCCO YREHWL----------------------LHCRLLVVSERLAKLAREL--GWQDIKVADNA 236

NeiME FKSL---------------------------LYFTHHPRIAEALKRE--GVCSVETVPTL 224

RICP LLQY-----------------------LQDSLVIAISLKVANIVRPF---IKNVVYCDNQ 254

Wolb LSNVMD-----------------------NITAYAMSKNIVDSLKPIK--WKKIITSRLP 222

wMel LSHVMN-----------------------NTVAYTMSKNIADSLKLIK--WKKIITSRLP 222

wSim LSHVMN-----------------------NTVAYTMSKNIADSLKLIK--WKKIITSRLP 222

AGRO YVTA-----------------------LAATKFICISRNVLFLVPEI--FRANAKAAEEP 231

BACSU LKKWK----------------------ANGTACISIGPLTNDALLTY--GITSHTPDT-F 243

MUS TPQALAAGIRNVLKPNHCC----------------------------------------- 265

HOMO TPQALATGIRKALQPHGCC----------------------------------------- 265

SACC DPKSLLDAIELYQRHK-------------------------------------------- 275

SCHPO NPESLASSIVAFDEENSS------------------------------------------ 251

SYNC TLDGLLLAIEQWARQTT------------------------------------------- 264

SYNE TLEGLTGAITNYVYQNP------------------------------------------- 538

TOXO GLAGWTETLFRAIENHVEEEKKKRRKVCCEESERCGSEGERDAGHGNRGSRDKDQFQRSN 1073

Arath GLEGWVESIMEALGAHADSSNPSSRN---------------------------------- 321

CYAME GLEAWVDTLVRVFEQKRTETRG-------------------------------------- 299

THAPS GVDGWAVSVADAL----------------------------------------------- 279

ESCCO DNDALLRALQ-------------------------------------------------- 246

NeiME EAALSHSSISVSDGMVFPGTSN-------------------------------------- 246

RICP SPHDIIKLLYENAKI--------------------------------------------- 269

Wolb TQENLIDIINKDC----------------------------------------------- 235

wMel TGESLIDIINKDC----------------------------------------------- 235

wSim TGESLIDIINKDC----------------------------------------------- 235

AGRO SEAAMFELLHHNSGT--------------------------------------------- 246

BACSU TIDGMLELMCSMSREEERI----------------------------------------- 262

:

MUS ------------------------------------------------------------

HOMO ------------------------------------------------------------

SACC ------------------------------------------------------------

SCHPO ------------------------------------------------------------

SYNC ------------------------------------------------------------

SYNE ------------------------------------------------------------

TOXO EKVRVLVLLTREEGKNMELVNLLWERGVSVDFAALKDSLKGAATDNGNQEDATQTSASSA 1133

Arath ------------------------------------------------------------

CYAME ------------------------------------------------------------

THAPS ------------------------------------------------------------

ESCCO ------------------------------------------------------------

NeiME ------------------------------------------------------------

RICP ------------------------------------------------------------

Wolb ------------------------------------------------------------

wMel ------------------------------------------------------------

wSim ------------------------------------------------------------

AGRO ------------------------------------------------------------

BACSU ------------------------------------------------------------

MUS ------------------------------------------------------------

HOMO ------------------------------------------------------------

SACC ------------------------------------------------------------

SCHPO ------------------------------------------------------------

SYNC ------------------------------------------------------------

SYNE ------------------------------------------------------------

TOXO KSPSSSSPLHSPSLSSSFPPNASTIPLLREPAGEGEVRPVAGDIEGVSATKDGGPGTSLE 1193

Arath ------------------------------------------------------------

CYAME ------------------------------------------------------------

THAPS ------------------------------------------------------------

ESCCO ------------------------------------------------------------

NeiME ------------------------------------------------------------

RICP ------------------------------------------------------------

Wolb ------------------------------------------------------------

wMel ------------------------------------------------------------

wSim ------------------------------------------------------------

AGRO ------------------------------------------------------------

BACSU ------------------------------------------------------------

MUS ------------------------------------------------------------

HOMO ------------------------------------------------------------

SACC ------------------------------------------------------------

SCHPO ------------------------------------------------------------

SYNC ------------------------------------------------------------

SYNE ------------------------------------------------------------

TOXO DKVKENEKDTPTGEEGERHGGVREKRHNIVMKVLEVPLLRTEAFRGVFKREQSILEELLA 1253

Arath ------------------------------------------------------------

CYAME ------------------------------------------------------------

THAPS ------------------------------------------------------------

ESCCO ------------------------------------------------------------

NeiME ------------------------------------------------------------

RICP ------------------------------------------------------------

Wolb ------------------------------------------------------------

wMel ------------------------------------------------------------

wSim ------------------------------------------------------------

AGRO ------------------------------------------------------------

BACSU ------------------------------------------------------------

MUS ----------------------

HOMO ----------------------

SACC ----------------------

SCHPO ----------------------

SYNC ----------------------

SYNE ----------------------

TOXO SNVVSAVESHAIGSSPRETPKK 1275

Arath ----------------------

CYAME ----------------------

THAPS ----------------------

ESCCO ----------------------

NeiME ----------------------

RICP ----------------------

Wolb ----------------------

wMel ----------------------

wSim ----------------------

AGRO ----------------------

BACSU ----------------------

**CLUSTAL X (1.83) multiple sequence alignment for UROD (5)**

**27 taxa/243 characters from underlined conserved regions are used for phylogeny analysis**

**Legend for abbreviations used in the alignment:**

**CAULO** 351aa NP_422557 uroporphyrinogen decarboxylase [Caulobacter crescentus CB15] alpha-proteobacteria

**AGRTU** 344aa NP_533500 uroporphyrinogen decarboxylase [Agrobacterium tumefaciens str. C58 (U. Washington)] alpha-proteobacteria

**RHOD** 344aa AAA97435 uroporphyrinogen decarboxylase [Rhodobacter capsulatus] alpha-proteobacteria

**RICPR** 345aa Q9ZC83 Uroporphyrinogen decarboxylase (URO-D) Rickettsia prowazekii alpha-proteobacteria

**Wolb** AAW70593 347aa Uroporphyrinogen-III decarboxylase [Wolbachia endosymbiont strain TRS of Brugia malayi alpha-proteobacteria

**wAna** ZP_00374193 301aa Uroporphyrinogen-III decarboxylase [Wolbachia endosymbiont Drosophila ananassae alpha-proteobacteria

**wSim** ZP_00372469 296aa Uroporphyrinogen-III decarboxylase [Wolbachia endosymbiont Drosophila simulans alpha-proteobacteria

**wWill** ZP_01314807 338aa Uroporphyrinogen-III decarboxylase [Wolbachia endosymbiont Drosophila willistoni TSC#14030-0811.24 alpha-proteobacteria

**wMel** NP_966753 338aa Uroporphyrinogen-III decarboxylase [Wolbachia endosymbiont Drosophila melanogaster alpha-proteobacteria

**NEIME** 354aa Q9K041 Uroporphyrinogen decarboxylase (URO-D) (UPD)Neisseria meningitidis serogroup B Beta-proteobacteria

**ESCCO** 354aa NP_418425 uroporphyrinogen decarboxylase [Escherichia coli K12] Gamma-proteobacteria

**TOXO** 665 aa DQ029343 uroporphyrinogen decarboxylase Toxoplasma gondii Apicomplexa/Alveolates

**PLAFA** 374aa CAD50347 uroporphyrinogen decarboxylase, putative [Plasmodium falciparum 3D7] Apicomplexa/Alveolates

**PLAYO** 373aa EAA21978 uroporphyrinogen decarboxylase, putative [Plasmodium yoelii yoelii] Apicomplexa/Alveolates

**SCHPO** 370aa Q9USJ5 Uroporphyrinogen decarboxylase Schizosaccharomyces pombe (fission yeast)/ Fungi

**SACCE** 362aa P32347 Uroporphyrinogen decarboxylase (URO-D) (UPD)Saccharomyces cerevisiae/ Fungi

**MUS** 367aa P70697 UROPORPHYRINOGEN DECARBOXYLASE (URO-D) (UPD) Mus musculus (house mouse)/ Animals

**HOMO** 367aa P06132 Uroporphyrinogen decarboxylase (URO-D) (UPD) Homo sapiens (human)/ Animals

**SYNE** 350aa NP_442753 uroporphyrinogen decarboxylase [Synechocystis sp. PCC 6803] Cyanobacteria

**SYNC** 354aa A56609 uroporphyrinogen decarboxylase (EC 4.1.1.37) - Synechococcus sp. (strain PCC 7942) Cyanobacteria

**TOBACCO** 391aa S55732 uroporphyrinogen decarboxylase - common tobacco/ Nicotiana tabacum/ Green plants

**ARAT** 394aa O22886 UROPORPHYRINOGEN DECARBOXYLASE PRECURSOR (UPD) Arabidopsis thaliana/ Green plants

**CYAME1*** 443aa CME194C uroporphyrinogen decarboxylase retrieved from http://merolae.biol.s.u-tokyo.ac.jp/ Cyanidioschyzon merolae Genome Project, Cyanidioschyzon merolae red algae

**CYAME2*** 455aa CMP083C uroporphyrinogen decarboxylase retrieved from http://merolae.biol.s.u-tokyo.ac.jp/ Cyanidioschyzon merolae Genome Project, Cyanidioschyzon merolae red algae

**Ciliate** EAR94688 398aa uroporphyrinogen decarboxylase retrieved from http://tigrblast.tigr.org/tgi/TIGR "Tetrahymena thermophila genome sequencing project, Tetrahymena thermophila Ciliates/Alveolates

**THAPS1** EED94619 392aa grail.20.112.1 uroporphyrinogen decarboxylase retrieved from http://genome.jgi-psf.org/thaps1/thaps1.home.html Thalassiosira pseudonana Diatoms/Heterokonts

**THAPS2** EED94520 403aa incomplete genewise.8.624.1 uroporphyrinogen decarboxylase retrieved from http://genome.jgi-psf.org/thaps1/thaps1.home.html Thalassiosira pseudonana Diatoms/Heterokonts

**BACSU** 353aa CAA74518 Uroporphyrinogen III decarboxylase [Bacillus subtilis] Firmicutes/G+ Bacteria

SYNE ------------------------------------------------------------

SYNC ------------------------------------------------------------

TOBACCO ------------------------------------------------------------

ARAT ------------------------------------------------------------

THAPS1 ------------------------------------------------------------

CYAME1 ------------------------------------------------------------

CYAME2 ------------------------------------------------------------

THAPS2 ------------------------------------------------------------

SCHPO ------------------------------------------------------------

SACCE ------------------------------------------------------------

MUS ------------------------------------------------------------

HOMO ------------------------------------------------------------

NEIME ------------------------------------------------------------

ESCCO ------------------------------------------------------------

Ciliate ------------------------------------------------------------

AGRTU ------------------------------------------------------------

RHOD ------------------------------------------------------------

CAULO ------------------------------------------------------------

RICPR ------------------------------------------------------------

Wolb ------------------------------------------------------------

wWill ------------------------------------------------------------

wMel ------------------------------------------------------------

wAna ------------------------------------------------------------

wSim ------------------------------------------------------------

PLAFA ------------------------------------------------------------

TOXO MQASSSSPSLLDERESSLPPRLSIFVRTKSMSVLHLLSCSRTPRPLRLRSPFSLVCLCFF 60

BACSU ------------------------------------------------------------

SYNE ------------------------------------------------------------

SYNC ------------------------------------------------------------

TOBACCO ------------------------------------------------------------

ARAT ------------------------------------------------------------

THAPS1 ------------------------------------------------------------

CYAME1 ------------------------------------------------------------

CYAME2 ------------------------------------------------------------

THAPS2 ------------------------------------------------------------

SCHPO ------------------------------------------------------------

SACCE ------------------------------------------------------------

MUS ------------------------------------------------------------

HOMO ------------------------------------------------------------

NEIME ------------------------------------------------------------

ESCCO ------------------------------------------------------------

Ciliate ------------------------------------------------------------

AGRTU ------------------------------------------------------------

RHOD ------------------------------------------------------------

CAULO ------------------------------------------------------------

RICPR ------------------------------------------------------------

Wolb ------------------------------------------------------------

wWill ------------------------------------------------------------

wMel ------------------------------------------------------------

wAna ------------------------------------------------------------

wSim ------------------------------------------------------------

PLAFA ------------------------------------------------------------

TOXO VALFHFLGAFSTCRQLPVSSRGDAAHMDMSWWRGPGVHTAHAYGFSEDRGETGLHSLAPS 120

BACSU ------------------------------------------------------------

SYNE ------------------------------------------------------------

SYNC ------------------------------------------------------------

TOBACCO ------------------------------------------------------------

ARAT ------------------------------------------------------------

THAPS1 ------------------------------------------------------------

CYAME1 ---------------------------------------------------MSRLAARRS 9

CYAME2 ------------------------------------------------------------

THAPS2 ------------------------------------------------------------

SCHPO ------------------------------------------------------------

SACCE ------------------------------------------------------------

MUS ------------------------------------------------------------

HOMO ------------------------------------------------------------

NEIME ------------------------------------------------------------

ESCCO ------------------------------------------------------------

Ciliate ------------------------------------------------------------

AGRTU ------------------------------------------------------------

RHOD ------------------------------------------------------------

CAULO ------------------------------------------------------------

RICPR ------------------------------------------------------------

Wolb ------------------------------------------------------------

wWill ------------------------------------------------------------

wMel ------------------------------------------------------------

wAna ------------------------------------------------------------

wSim ------------------------------------------------------------

PLAFA ------------------------------------------------------------

TOXO LSGNSSRTERHPHHVLPRVSQSQEERRETEERDSEDAARAKNEWTAAEKEQLLQSSLGFF 180

BACSU ------------------------------------------------------------

SYNE ------------------------------------------------------------

SYNC ------------------------------------------------------------

TOBACCO -----------------------------------------MMSCNYSFSSISPSSKSAF 19

ARAT --------------------------------------MSILQVSTSSLSSSTLLSISPR 22

THAPS1 ----------------------------------------------------MKFAAALF 8

CYAME1 PDARSRCRSPTSRRGRRRGCGTAQRQTAEKAEAHTRPMFLLPSGTLETVFCGRRAYTSRP 69

CYAME2 -------------------MFQFAVLKLTRTPRCAGHTAPRSLQAASGAKFRLRSERLRY 41

THAPS2 ----MKVVQHLLSIQLLSTAVAFTPATTSRSP---------------------------- 28

SCHPO ------------------------------------------------------------

SACCE ------------------------------------------------------------

MUS ------------------------------------------------------------

HOMO ------------------------------------------------------------

NEIME ------------------------------------------------------------

ESCCO ------------------------------------------------------------

Ciliate ------------------------------------MSEADKQAEQPRTIEVPLLQSTLA 24

AGRTU ------------------------------------------------------------

RHOD ------------------------------------------------------------

CAULO ------------------------------------------------------------

RICPR ------------------------------------------------------------

Wolb ------------------------------------------------------------

wWill ------------------------------------------------------------

wMel ------------------------------------------------------------

wAna ------------------------------------------------------------

wSim ------------------------------------------------------------

PLAFA ------------------------------------------------------------

TOXO SGGVRSFFFPKGTANMTLESAGSERTDDAPPALYTLTPDDERALIRSQRTAYKANPREPL 240

BACSU ------------------------------------------------------------

SYNE ------------------------MTEANDLPY-LLRVARGEVVKRPPVWMMR------- 28

SYNC ------------------------MVASSSLPR-LLRAARGEVLDRPPVWMMR------- 28

TOBACCO TSPSNFNLNPRLICCSAGGTVAEPKAINATQPL-LLDAVRGKEVERPPVWLMR------- 71

ARAT KSLSSTKSCRIVRCSVEGTTVTERKVSATSEPL-LLRAVKGEVVDRPPVWLMR------- 74

THAPS1 LLASGANGFSATTNTQPVPTTNIDANGKTIDPL-LIRAARGETTERVPVWMMR------- 60

CYAME1 KRFFSLKAVEASGQETASSSSAAGERAQAMQPL-LVRAIRHEQVERPPIWLMR------- 121

CYAME2 HVASLALRAALDTDYEGCLSESSVVETCNEAPLLLRVLRCDPSVPRPPVWFMR------- 94

THAPS2 --------TALHSSTEAVVESTANGVNKGDRDI-LVRCASGQQVERTPVWLMR------- 56

SCHPO ----------------------------MKNDL-ILRAAKGEEVERPPVWIMASWKISSW 31

SACCE ----------------------MGNFPAPKNDL-ILRAAKGEKVERPPCWIMR------- 30

MUS ---------------MEANGFGLQNFPELKNDT-FLRAAWGEETDYTPVWCMR------- 37

HOMO ---------------MEANGLGPQGFPELKNDT-FLRAAWGEETDYTPVWCMR------- 37

NEIME -------------------------MISLKNDT-FLRALLKQPVEYTPIWMMR------- 27

ESCCO -------------------------MTELKNDR-YLRALLRQPVDVTPVWMMR------- 27

Ciliate RLGIDKNSENWRQQVRALRQSKDQKFPVMKNDT-LLRVIKREKIDHLPIWVMR------- 76

AGRTU -----------------------------MSERKVMTVLNGKTVTPPPIWLMR------- 24

RHOD -----------------------------MTDKTILRALKGEVLPTPPVWLMR------- 24

CAULO ---------------------MTSSSPILKQTPKFLSALEGQSHANPPVWFMR------- 32

RICPR -------------------------------MKQ--ILNPLKNSNKIPIWFMR------- 20

Wolb -----------------MKEESELARIKSSKAVVSKTIKEGKQGRKIPIWLMR------- 36

wWill --------------------------MESGKTTIAKIIKRNEPGGRVPIWLMR------- 27

wMel --------------------------MESGKTTIAKIIKRNEPGGRVPIWLMR------- 27

wAna ------------------------------------------------------------

wSim ------------------------------------------------------------ 2

PLAFA ----MNEVGEIYSRPENLKYEKFGRPQNDLILNVIENKNEEKKFKKIPLWIMR------- 49

TOXO VNDSLRRAALGPARYARLVEKTVELATAGRHGGDTEQKTAGEEPFLTPVWMMR------- 293

BACSU -----------------------MSKRETFNETF-LKAARGEKADHTPVWYMR------- 29

SYNE --------QAGRYMKVYRDLRDKYPSFRERSENPDLAIEISLQPWQAFQ--PDGVIMFSD 78

SYNC --------QAGRYMKVYRDLRDKYPGFRERSETPELAIEISLQPFRAFK--PDGVILFSD 78

TOBACCO --------QAGRYMKSYQLLCEKYPLFRDRSENVDLVVEISLQPWKVFR--PDGVILFSD 121

ARAT --------QAGRYMKSYQTLCEKYPSFRDRSENADLVVEISLQPWKVFK--PDGVILFSD 124

THAPS1 --------QAGRHIKEYRDLCKKYPTFRERSEIPEVAVEVSLQPWRNYQ--TDGCILFSD 110

CYAME1 --------QAGRHMNAYRELCKRHPSFRERSEVAALSTEISLQPYRAYH--PDAVIMFSD 171

CYAME2 --------QAGRYMSAFRMYSGRYA-FRERAETPEIAVELSLQPWRAFG--VDAVIMFAD 143

THAPS2 --------QAGRYMSAFRQYSDKYP-FRERSETPSMAIELSLQCHRAYG--MDGIIMFSD 105

SCHPO YVHDLNLIVCTNETIEYHKLRAKQ-SFFEMCQTPETACELTLQPVTRFKGLLDAAIIFSD 90

SACCE --------QAGRYLPEYHEVKNNR-DFFQTCRDAEIASEITIQPVRRYRGLIDAAIIFSD 81

MUS --------QAGRYLPEFRETRAAQ-DFFSTCRSPEACCELTLQPLRRFP--LDAAIIFSD 86

HOMO --------QAGRYLPEFRETRAAQ-DFFSTCRSPEACCELTLQPLRRFP--LDAAIIFSD 86

NEIME --------QAGRYLPEYKATRAKAGSFLDLCKNTELATEVTIQPLERFD--L-AAILFSD 76

ESCCO --------QAGRYLPEYKATRAQAGDFMSLCKNAELACEVTLQPLRRYP--LDAAILFSD 77

Ciliate --------QAGRYLPEFREVRKIW-SFFELCDNPLLAAEVTLQPLERFP--MDAGIIFSD 125

AGRTU --------QAGRYLPEYRETRKSAGSFLDLCYNPELASEVTLQPIRRFG--LDAAILFSD 74

RHOD --------QAGRYLPEYRATRAQAGDFLSLCYTPDLAAEVTLQPIRRYG--FDAAILFAD 74

CAULO --------QAGRYLPEYRAVRATAPDFISFCFDPEKAAEVTLQPMRRFP--FDASIVFAD 82

RICPR --------QAGRYLPEYKKVRETTKNFLDFCYDVNKATEVTLQPIRRYG--LDAAIIFSD 70

Wolb --------QAGRSLPEYRRAVKDTSSFMEICYNTDLVVELTLQPVARFS--MDAAIIFSD 86

wWill --------QAGRSLPEYRKAVENMNNFMEICYNTDLVTELTLQPVTRFD--MDAAIIFSD 77

wMel --------QAGRSLPEYRKAVENMNNFMEICYNTDLVTELTLQPVTRFD--MDAAIIFSD 77

wAna ------------------KAVENMNNFMEICYNTDLVTELTLQPVTRFD--MDAAIIFSD 40

wSim -----------------------MNNFMEICYNTDLVTELTLQPVTRFD--MDAAIIFSD 35

PLAFA --------QAGRYLPEYVEIRKKY-DFFEICKSPDLSSYVSIMPYDRFK--TDMIVIFSD 98

TOXO --------QAGRYLPEFRNMRRQHG-FLEVCRDPRLASELTLQPYRRFP-QLDAAIIFSD 343

BACSU --------QAGRSQPEYRKLKEKYG-LFEITHQPELCAYVTRLPVEQYG--VDAAILYKD 78

: . :: : ::: *

SYNE ILTPLPGIGIPFDIIESKGPIIDPPIRTQAQVDQLHALDP---ESSLPFIKTILGTLRKE 135

SYNC ILTPLPGMGIPFDIIESKGPILEPPIRTAEQVAAVHDLDP---EEATPFIRPILETLRQE 135

TOBACCO ILTPLSGMNIPFDIIKGKGPVIFDPLRTAADVEKVREFIP---EKSVPYVGEALTILRKE 178

ARAT ILTPLSGMNIPFDIVKGKGPIIFNPPQSAADVAQVREFVP---EESVPYVGEALRRLRNE 181

THAPS1 ILTPLPGMGVEFDIDE-KVGPVVKPMRTWDDVNKMHLIDP---SKSIPFTAEALRILRQE 166

CYAME1 ILTPLPGMGITFDIPE-RGPVVSPPVRSYDDLQRFREMDP---YKSTPFVRETLQTLRRV 227

CYAME2 ILTPLPPMGLDYQIVSGQGPSIKTPVRCMEDVELVRPLTPEDTETALSFVGTTLSTLAHT 203

THAPS2 ILTPLPTLGIEFDVVKGLGPVISTEVRTEADVAKLKDVESVNFDETLPFIREILGSLSKE 165

SCHPO ILVIPQALGMQVVMLEQKGPHFPKPLVVPEDID-LLEKTPN-ISAKLGYVMDAISLTREK 148

SACCE ILVIPQAMGMRVEMLEGKGPHFPEPLRNPEDLQTVLDYKVD-VLKELDWAFKAITMTRIK 140

MUS ILVVPQALGIEVTMVPGKGPSFPEPLREERDLERLRDPAA--AASELGYVFQAITLTRQR 144

HOMO ILVVPQALGMEVTMVPGKGPSFPEPLREEQDLERLRDPEV--VASELGYVFQAITLTRQR 144

NEIME ILTVPDAMGLGLYFAEGEGPKFKRALQHEADIAKLHVPDME----KLQYVFDAVTSIRKA 132

ESCCO ILTVPDAMGLGLYFEAGEGPRFTSPVTCKADVDKLPIPDP---EDELGYVMNAVRTIRRE 134

Ciliate ILVVPKVMGMEVIMEEKRGPVFPQPLVEPSDLQKLSIPDP----ESLGDVYDALYLFRQA 181

AGRTU ILVIPDALHRNVSFSEGKGPAMD-PIDISGIEKLDASDVM----AHLSPVFETVSRLRTS 129

RHOD ILLLPQALGLDLWFETGEGPRMS-TVTSMEGVKGLKGKDD--IHDKLAPVYETCKILSRE 131

CAULO ILLIPGALGQKVWFEAGEGPKLG-DMPSVESMAEKAGEAG----KALSLVGETLTRVRSA 137

RICPR ILVLPNALGWEVDFKENIGPVLK-QFKSQKDFQYLQRDSN----NKLEKVYKIIKKVKEK 125

Wolb ILIVADVLGCDVDFIRGVGPTIK-PVKNFKELRNPQKIET-----KTLPILNAIRKVRDK 140

wWill ILIIADVLGCDVNFVRGVGPIIK-PVESPKELKGPQEIET-----KTLPILNAIKKVRSQ 131

wMel ILIIADVLGCDVNFVRGVGPIIK-PVESPKELKGPQEIET-----KTLPILNAIKKVRSQ 131

wAna ILIIADVLGCDVNFVRGVGPIIK-PVESPKELKGPQEIET-----KTLPILNAIKKVRSQ 94

wSim ILIIADVLGCDVNFVRGVGPIIK-PVESPKELKGPQEIET-----KTLPILNAIKKVRSQ 89

PLAFA ILIIFVAMGIDIKFVENLGPVFNLEIYNMNDFNKLNLNMKE-IIDNLYYVYDSINLTKKK 157

TOXO ILVIPEAMGMKLSMEEGVGPRFAWRIESPADMRKLNFKPD--IEGTLGYVFDAVYVTSQQ 401

BACSU IMTPLPSIGVDVEIKNGIGPVIDQPIRSLADIEKLGQIDP---EQDVPYVLETIKLLVNE 135

*: : . .

SYNE VGNQ---STVLGFVGAPWTLAAYAIEGKSS--KDYKVIKQMAFSEPA----ILHSFLDKI 186

SYNC VGNE---AAVLGFAGAPWTLAAYAIEGKSS--KTYANIKHLAFSEPT----ILHELLGKL 186

TOBACCO VNNQ---AAVLGFVGAPFTLASYVVEGGSS--KNFTKIKRLAFAEPK----VLHALLQKF 229

ARAT VNNE---AAVLGFVGAPFTLSSYVIEGGSS--KNFTQIKRLAFSQPK----VLHALLQKF 232

THAPS1 VTPE---TAVLGFVGCPYTLATYLVEGKTS--KEYLEIKKMAFTEPK----LLHAILKNL 217

CYAME1 VEPE---VAVLGFVGAPWTLLTYCIEGGSS--QTYSQIKKVAFTEPA----LLHELLARV 278

CYAME2 LADARPRPALLGFLGAPFTLAAYGIEGEGGRASSGKQVKRMMYTAEGRE--LMHRLLQKI 261

THAPS2 AEEAN--TSLIGFVGAPFTLAAYTIEGKSS--KHCLSTKKMMMADEDGSSKAVSQFLDKL 221

SCHPO LDGQ---VPLMGFSGAPWTIMAYMIEGGGS--KTFAKAKSWLFRYPE----ASHKLLKII 199

SACCE LDGE---VPLFGFCGGPWTLMVYMTEGGGS--RLFRFAKQWINMYPE----LSHKLLQKI 191

MUS LAGR---VPLIGFAGAPWTLMTYMVEGGSS--STMAQAKRWLYQRPQ----ASHKLLGIL 195

HOMO LAGR---VPLIGFAGAPWTLMTYMVEGGGS--STMAQAKRWLYQRPQ----ASHQLLRIL 195

NEIME LDGR---VPLIGFSGSPFTLACYMVEGGGS--KEFRTIKTMMYSRPD----LLHKILDTN 183

ESCCO LKGE---VPLIGFSGSPWTLATYMVEGGSS--KAFTVIKKMMYADPQ----ALHALLDKL 185

Ciliate VEGK---VTTIGFAGAPWTLMSYMIEGGGS--KMFSKVKKWIFKWKE----ESKKLLSMI 232

AGRTU LPDE---TTLLGFCGAPWTVATYMIAGHGT--PDQAPARLFGYQEPA----AMEKLLALL 180

RHOD LPKE---TTFIGFAGMPWTVATYMIAGRGS--KDQAAAHKFKDTDRA----AFSALIDAV 182

CAULO LDPD---KALIGFAGAPWTVATYMIE-KGS--SDRSGARTFAYQNPE----TLDALIQVL 187

RICPR LPSN---TSLIGFAGSPWTVMSYMLEGKGK--RDFRTSKKFIYENKI----LAKDLLNFI 176

Wolb LPEE---KSLIGFAGGPWTVASYIIEGRSS-----KTFSKVLNLHPS----SLEQIIEQI 188

wWill LPKE---KSLIGFAGGPWTVASYIIEGRSS-----KTFSKVLNFYPS----CLKEIIERI 179

wMel LPKE---KSLIGFAGGPWTVASYIIEGRSS-----KTFSKVLNFYPS----CLKEIIERI 179

wAna LPKE---KSLIGFAGGPWTVASYIIEGGSS-----KTFSKVLNFYPS----CLKEIIERI 142

wSim LPKE---KSLIGFAGGPWTVASYIIEGGSS-----KTFSKVLNFYPS----CLKEIIERI 137

PLAFA INND---VPILGFCGSPFTLFMYLTKNNK---KTYEDSFKFLYEKPN----DAHQIINKL 207

TOXO LAGA---IPLIGFCGGPLTLLTYMVEGGGG--KTWRQAKKFVYEHPE----ATHTLLQVI 452

BACSU QLN----VPLIGFSGAPFTLASYMIEGGPS--KNYNKTKAFMYSMPD----AWNLLMSKL 185

. :** * * *: * ::

SYNE AEAIAVYVRYQIDCGAQVVQLFDSWAGQLSPQDYDTFALPYQQKVVKLVKEI-------- 238

SYNC ADNIAIYLCHQIDCGAQVVQLFDSWAGQLSPIDYDTFALPYQQRVFQQVKAK-------- 238

TOBACCO ATSMAKYIRYQADSGAQAVQIFDSWATELSPVDFEEFSLPYLKQIVDSVKLT-------- 281

ARAT TTSMITYIRYQADSGAQAVQIFDSWATELSPVDFEEFSLPYLKQIVEAVKQT-------- 284

THAPS1 SESIAEYALFQIENGAQLIQIFDSWAGHLSPRDYDEFAAPYQKVILDKIKEK-------- 269

CYAME1 TDNLAIYANFQIECGADAIQIFDSWAGQLAPSDFDEFAGPYLNRLVREVKAS-------- 330

CYAME2 TQSMIVFAAYQARKGAEAIQIFDSWAHLLSPEDYQEFSLFYVEELVRGIRAH-------- 313

THAPS2 AVMIGNYACHQIECGAQVIQVFESWAHQLSPRQFEAFAKPAAQKAIRIIKEK-------- 273

SCHPO TDATVSYLIQQVYAGAQLLQIFDSWAGELSPEDFTEYAYPYLVRICQEVKQHL------- 252

SACCE TDVAVEFLSQQVVAGAQILQVFESWGGELSSVDFDEFSLPYLRQIAERVPKRL------- 244

MUS TDVLVPYLIGQVAAGAQALQLFESHAGHLGTELFSKFALPYIRDVAKRVKAGL------- 248

HOMO TDALVPYLVGQVVAGAQALQLFESHAGHLGPQLFNKFALPYIRDVAKQVKARL------- 248

NEIME AQAVTAYLNAQIDAGAQAVQIFDTWGGVLSDAAFKEFSLKYIRQIVAGLKR--------- 234

ESCCO AKSVTLYLNAQIKAGAQAVMIFDTWGGVLTGRDYQQFSLYYMHKIVDGLLR--------- 236

Ciliate SDVLIEYISKQIEGGAQIIQLFDSWAGELGHEDYWEFGLSYCVRVATEVKKR-------- 284

AGRTU AEVSADYLVAQIDAGADAVQIFDSWAGVLGEKEFEEYAIKPVARIIASVRAR-------- 232

RHOD TVATIEYLSKQVEAGCEVVKLFDSWAGSLKGQDFEDFAVEPARVITAEMKRR-------- 234

CAULO VDATIDYLAMQVDAGAQALKLFESWAEGLSEPLFDRLVTQPHIRIIEGLRAR-------- 239

RICPR TEKTSDYLINQVKSGVDLLKIFDSWSGVLAEDDFTEFVIEPTKKIILKVKEV-------- 228

Wolb TEVTISYLVKQIEFGADVIQLFDSNAGVLSGELFEECVIKPTKKIVSAIKGK-------- 240

wWill TEVTIIYLIKQIEFGADVIQLFDSNAGALSEPLFKEYVIEPTKRIILAIKDR-------- 231

wMel TEVTIIYLIKQIEFGADVIQLFDSNAGALSEPLFKEYVIEPTKRIILAIKDR-------- 231

wAna TEVTIIYLIKQIEFGADVIQLFDSNAGALSEPLFKEYVIEPTKRIILAIKDR-------- 194

wSim TEVTIIYLIKQIEFGADVIQLFDSNAGALSEPLFKEYVIEPTKRIILAIKDR-------- 189

PLAFA SDICLNHLINQIDSGANIIQIFDSNAELVDKNIFNEFSIIYLKKVIEIIKKY-------- 259

TOXO TDICVEYLVGQVDAGAQVLQVFDTNASQFAAEAYDEIGAPYMASIAKRVKARRPHVTMIA 512

BACSU ADMIIVYVKAQIEAGAKAIQIFDSWVGALNQADYRTYIKPVMNRIFSELAK--------- 236

. * * . : :*:: . : :

SYNE -----------HPDTPLILYISGSAGILERMGKSG------------VDIVSVDWTVDMA 275

SYNC -----------HPEVPLILYISGSAGVLERMGQSG------------CDIVSVDWTVDLL 275

TOBACCO -----------HPNLPLILYASGSGGLLERLPLTG------------VDVVSLDWTVDMA 318

ARAT -----------HPNLPLILYASGSGGLLERLARTG------------VDVVSLDWTVDMA 321

THAPS1 -----------YPTVPTVTYIKHSGALIERMAATG------------VDVVSLDWTVDMA 306

CYAME1 -----------HPETPLILYINQGSHLLERMRETG------------VDVVSVDWTIDIG 367

CYAME2 -----------GVETPLIFFANGSCGKLGVISAMYPADTREANGYRGLDALALDWRTSIA 362

THAPS2 -----------YPDVPVIYFANGGSSYLELQKDMG------------CDMIAVDWSVDMK 310

SCHPO ------KKKK-RDEVPMIVFAKGAWYAIDQLCDSG------------YDVIGLDWTVSPK 293

SACCE ------QELGIMEQIPMIVFAKGSWYALDKLCCSG------------FDVVSLDWSWDPR 286

MUS ------QKAG-LAPVPMIIFAKDGHFALEELAQAG------------YEVVGLDWTVAPK 289

HOMO ------REAG-LAPVPMIIFAKDGHFALEELAQAG------------YEVVGLDWTVAPK 289

NEIME ------ESEG-R-RVPVIVFAKGGGLWLESMAQIG------------ADALGLDWTCNIG 274

ESCCO ------ENDG-R-RVPVTLFTKGGGQWLEAMAETG------------CDALGLDWTTDIA 276

Ciliate -----------HPETPIIMFAKGNHSDMVFKHDCF-------------DCVGIDYAWDLS 320

AGRTU -----------RPSAKIIAFAKGAGYLLRDYRRKTG-----------ANAIGLDWSVPLS 270

RHOD -----------FPGLPVIAFPREAGQGYIGFAEKTG-----------ADCVAIDNSVSPD 272

CAULO -----------GVTVPIIGFPRGAGTLVEDYAARTP-----------VQGVALDTSASAK 277

RICPR -----------FVKTPIITFPKGAGLLYEKFIEEVP-----------IDILAVDQMVPLE 266

Wolb -----------FPDFPIIGFPKSAGSLYKDYCEKTG-----------VSAVSIDYNVPIE 278

wWill -----------FSDFPIIGFPRSAGNLYKDYCEQTG-----------VSAVSIDYNVPIK 269

wMel -----------FSDFPIIGFPRSAGNLYKDYCEQTG-----------VSAVSIDYNVPIK 269

wAna -----------FSDFPIIGFPRSAGNLYKDYCEQTG-----------VSAVSIDYNVPIK 232

wSim -----------FSDFPIIGFPRSAGNLYKDYCEQTG-----------VSAVSIDYNVPIK 227

PLAFA -----------RPNIYIILFIKNN---FHDDIKNLK-----------IDVLSITHKQLID 294

TOXO FPKDRPSAGFADSAFDVISLGSSAEIESMQRRFSGQCEEKIDQVTAGEAELTRSDASATE 572

BACSU ------------ENVPLIMFGVGASHLAGDWHDLP------------LDVVGLDWRLGID 272

:

SYNE DARQRLG----KEMKVQGNMDPGVLFG-SQDFIKERILDTVRKAGQ-GGHIFNLGHGVLV 329

SYNC DARRRLG----PDIGLQGNIDPGVLFG-SQDFIRDRILDTVRKAGN-QRHILNLGHGILP 329

TOBACCO DGRRRLG----PNVAIQGNVDPGVLFG-SKEFITNRINDTVKKAGK-GKHILNLGHGIKV 372

ARAT EGRDRLG----RDIAVQGNVDPGVLFG-SKEFITSRIHDTVKKAGR-DKHILNLGHGIKV 375

THAPS1 EGRERIA----AGRGVQGNLDPGVLFG-DFATIKERAEEIMKKAGP-TGHVMNLGHGIEA 360

CYAME1 EARSRLG----PRVAVQGNMDPACLLG-SRELIQRRAYEIITKAGK-TGHIMNLGHGVLP 421

CYAME2 EARQLFG----KRAVLQGNIDPTVLACGDTASVKAAIDRCVCAASG-GAHILNVGRGIQP 417

THAPS2 EARKILG----PDIPISGNIDPTILFG-TKEQIEQAVRDCIDKAGGPGKHLLNLGHGVMQ 365

SCHPO EAVRIRGN---RRVTFQGNLDPNILYGTREIIEARTKEMIQDFGGGKQGYIINLGHGITP 350

SACCE EAVKINK----NRVTLQGNLDPGVMYGSKEVITKKVKQMIEAFGGGKSRYIVNFGHGTHP 342

MUS KARERVG----KAVTLQGNLDPCALYASEEEIGRLVQQMLDDFG--PQRSIANLGHGLYP 343

HOMO KARECVG----KTVTLQGNLDPCALYASEEEIGQLVKQMLDDFG--PHRYIANLGHGLYP 343

NEIME EARRRVG----KQVALQGNFDPFALFGTPESIRTEVARILADYG-HGSGHVFNLGHGINQ 329

ESCCO DARRRVG----NKVALQGNMDPSMLYAPPARIEEEVATILAGFG-HGEGHVFNLGHGIHQ 331

Ciliate LAAKYSQQ---YNKVIQGNLDPGVLLAEKDTIREKTRKMIDIVG--VDRYIVNLGHGMWP 375

AGRTU FARDLQK-----EGPVQGNLDPMLMVAGG-KALQDGIDAVLQSLGQG-PLIFNLGHGITP 323

RHOD WAAENVQK---GKTCVQGNLDPSYMVTGG-QELVEATKKVVAAFKNG-PHIFNLGHGITP 327

CAULO LGQTIQK-----TKTIQGALDPLLLRAGG-DALLKRVDEMLEQWNQG-PYIFNLGHGILP 330

RICPR KMKEWSN-----KVIIQGNLDPVILLTNK-EIIKEKTYKILQAMKGK-NFIFNLGHGILP 319

Wolb WAKENLK------IPLQGNLNPSLLAYNKMEAIKETKRIIDCFR--DLPFIFNLGHGVLP 330

wWill WAKANLN------IPLQGNLDPNLLAYNKTEAIKEAKRIIDCFR--DLPFIFNLGHGVLP 321

wMel WAKANLN------IPLQGNLDPNLLAYNKTEAIKEAKRIIDCFR--DLPFIFNLGHGVLP 321

wAna WAKANLN------IPLQGNLDPNLLAYNKTEAIKEAKRIIDCFR--DLPFIFNLGHGVLP 284

wSim WAKANLN------IPLQGNLDPNLLAYNKTEAIKEAKRIIDCFR--DLPFIFNLGHGVLP 279

PLAFA NTDQFYFKLFQNNIILQGALDPYILLLNDHELIQKHIIQMMKNITHKNKYIANLGHGILP 354

TOXO SDRDRKSDVRGDRKALQGNLDPQVLYTDTATIKRETAKMIRRFGVG--RHIANLGHGMEP 630

BACSU EARSKGI-----TKTVQGNLDPSILLA-PWEVIEQKTKEILDQGMESDGFIFNLGHGVFP 326

..* .:* : : *.*:*

SYNE GTPEDNVRFFFETAKQVDQLL----------------- 350

SYNC GTPEDNARHFFETAKNLDQLLAASH------------- 354

TOBACCO GTPEENFAHFFEIAKGLRY------------------- 391

ARAT GTPEENVAHFFEVAQEIRY------------------- 394

THAPS1 ATPEENAAYFIETVRNYRHEGQE--------------- 383

CYAME1 ETPEENVLEFFRLVRGYRYDEH---------------- 443

CYAME2 DTPEEMVALFCEHVRALPETDWYQQLRHRHLVENGVPG 455

THAPS2 GTPEEAVGWLVDECKRYRGQNA---------------- 403

SCHPO GVNPDDVRFFLEKCHQYGSA------------------ 370

SACCE FMDPDVIKFFLEECHRIGSK------------------ 362

MUS DMDPERVGAFVDAVHKHSRLLRQN-------------- 367

HOMO DMDPEHVGAFVDAVHKHSRLLRQN-------------- 367

NEIME HADPEHAKILVDTVHELSRQYHGG-------------- 353

ESCCO DVPPEHAGVFVEAVHRLSEQYHR--------------- 354

Ciliate EHDLEHLAVFVDETHKYSASKLK--------------- 398

AGRTU QADPENVTRLVQRVRSVGGAG----------------- 344

RHOD EANPDNVTLLMETIRKG--------------------- 344

CAULO DTPIAHVEAVLERVTGQKVGQ----------------- 351

RICPR ETHPENVEFLTQYVRLYEQKNSNSTF------------ 345

Wolb NTPIENIAALVDLVKSQ--------------------- 347

wWill DTPVENIAALVNLVKSY--------------------- 338

wMel DTPVENIAALVNLVKSY--------------------- 338

wAna DTPVENIAALVNLVKSY--------------------- 301

wSim DTPVENIAALVNLVKSY--------------------- 296

PLAFA TTKIKNVNLFIDTIRNNEWS------------------ 374

TOXO EMKPEHAKAFIDGVKEASAAYIRELRGEGQDTSTV--- 665

BACSU DVSPEVLKKLTAFVHEYSQNKKMGQYS----------- 353

.

**CLUSTAL X (1.83) multiple sequence alignment for CPO (6)/HemF (O_2_-dependent)**

**23 taxa/232 characters from underlined conserved regions are used for phylogeny analysis**

**Legend for abbreviations used in the alignment:**

**RICPR** 279aa NP_221228 COPROPORPHYRINOGEN III OXIDASE (hemF) [Rickettsia prowazekii] Alph-proteobacteria

**AGRO 303aa** NP_532920 coproporphyrinogen III oxidase [Agrobacterium tumefaciens str. C58 (U. Washington)] Alph-proteobacteria

**CAULO** 290aa NP_419325 coproporphyrinogen III oxidase, aerobic [Caulobacter crescentus CB15] Alph-proteobacteria

**Wolb** AAW71297 274aa coproporphyrinogen III oxidase [Wolbachia endosymbiont strain TRS of Brugia malayi] Alph-proteobacteria

**wAna** ZP_00373119 274aa coproporphyrinogen III oxidase [Wolbachia endosymbiont of

Drosophila ananassae] Alph-proteobacteria

**wMel** NP_966926 256aa coproporphyrinogen III oxidase [Wolbachia endosymbiont of

Drosophila melanogaster] Alph-proteobacteria

**RALSO** 302aa Q8XXC3 Coproporphyrinogen III oxidase, aerobic Ralstonia solanacearum Beta-proteobacteria

**ESCC** 299aa NP_416931 coproporphyrinogen III oxidase [Escherichia coli K12] Gamma-proteobacteria

**SYNC** 388aa NP_898131 Coproporphyrinogen III oxidase [Synechococcus sp. WH 8102] Cyanobacteria

**SYNE** 340aa NP_440183 coproporphyrinogen III oxidase [Synechocystis sp. PCC 6803] Cyanobacteria

**PLAFA** 470aa AE014842 coproporphyrinogen III oxidase [Plasmodium falciparum 3D7] Apicomplexa/Alveolates

**TOXO** 364 aa DQ029344 coproporphyrinogen III oxidase, aerobic Toxoplasma gondii Apicomplexa/Alveolates

**SACCE** 328aa 2144339 coproporphyrinogen oxidase (EC 1.3.3.3) - yeast (Saccharomyces cerevisiae) Fungi

**SCHPO** 312aa NP_593150 coproporphyrinogen III oxidase [Schizosaccharomyces pombe] Fungi

**MUS** 354aa BAA03840 coproporphyrinogen oxidase [Mus musculus] Animals

**HOMO** 454aa NP_000088 coproporphyrinogen oxidase [Homo sapiens] Animals

**Ciliate** EAR84299 338aa aerobic form of coproporphyrinogen oxidase retrieved from http://tigrblast.tigr.org/tgi/TIGR "Tetrahymena thermophila genome sequencing project" Ciliates/Alveolates

**ARATH** 386aa CAD12661 coproporphyrinogen III oxidase [Arabidopsis thaliana] Green plants

**Tobaco** 397aa CAA58038 coproporphyrinogen oxidase [Nicotiana tabacum] Green plants

**CHLRE** 365aa AF133671 coproporphyrinogen III oxidase precursor [Chlamydomonas reinhardtii] Green algae

**CYAME** 406aa CMO136C Oxygen-dependent coproporphyrinogen III oxidase (HemF) retrieved from http://merolae.biol.s.u-tokyo.ac.jp/ Cyanidioschyzon merolae Genome Project, Cyanidioschyzon merolae red algae

**Maize-1** ABB77211 400aa plastid coproporphyrinogen III oxidase [Zea mays] plants

**Maize-2** ABB77212 420aa Mitochondrial coproporphyrinogen III oxidase [Zea mays] plants

AGRO ------------------------------------------------------------

CAULO ------------------------------------------------------------

RICPR ------------------------------------------------------------

Wolb ------------------------------------------------------------

wMel ------------------------------------------------------------

wAna ------------------------------------------------------------

RALSO ------------------------------------------------------------

ESCC ------------------------------------------------------------

SYNC ------------------------------------------------------------

SYNE ------------------------------------------------------------

CHLRE -------------------------------------------------MALQASTRSLQ 11

ARATH ---------------------------------------------------MASHSSTLL 9

Tobaco ----------------------------------------------MLTPILSSASCSWT 14

Maize-1 -----------------------------------------------------MASNLLS 7

Maize-2 ---------------------------------------------MIRFLIALEARQGRT 15

CYAME -----------------------------------------------MAPLCFTVAPARK 13

MUS ------------------------------------------------------------

HOMO MALQLGRLSSGPCWLVARGGCGGPRAWSQCGGGGLRAWSQRSAAGRVCRPPGPAGTEQSR 60

Ciliate ------------------------------------------------------------

SACCE ------------------------------------------------------------

SCHPO ------------------------------------------------------------

TOXO ------------------------------------------------------------

PLAFA ------------------------------------------------------------

AGRO -----------------------------------------------MERPILPKGLPED 13

CAULO ------------------------------------------------------MSTDQD 6

RICPR -----------------------------------------------------------M 1

Wolb ------------------------------------------------------------

wMel ------------------------------------------------------------

wAna ------------------------------------------------------------

RALSO ------------------------------------------------------------

ESCC ------------------------------------------------------------

SYNC --------------------MTVPDGVLSWAGITCLGSSIEWSAFMVRSLVRRLLGRSQN 40

SYNE --------------------MTVSP-----------------------------TTQPQT 11

CHLRE QRRAFSSAQTSKRVSVTKVR----------------------ATAIEAENYVKQAPQSLV 49

ARATH SSPTFAPFSSHRLHYSPNPSTLRFSR----PIRNKPNLALRCSVSIEKEVPETERPFTFL 65

Tobaco PTSQFPHSWHSSPSFLTKPLNLPFTESYKTAKRPTPNYSFKVQAMIEKEVAVSHKPDAFL 74

Maize-1 APSRTLAPSPAPAPAGFRGRVPVQAHFPRRGGFGFGFAPRRSSRALRVRASVAIEKETPE 67

Maize-2 PGARSHHLPPPLTLPAAKPQAASRPPPTAHLWQVATAAYINHQSTTRRTKPPTRNPMASN 75

CYAME NGSERLRRLGAERADGAFLAALCKSRWARFGRRTQVSGLHWKLTCISTHENVSEAPNSLL 73

MUS ----------------------------------------MVPKSSGARSPSPGRREEDG 20

HOMO GLGHGSTSRGGPWVGTGLAAALAGLVGLATAAFGHVQRAEMLPKTSGTRATSLGRPEEEE 120

Ciliate ----------------------------------------MISSLLKFLAPQ-------- 12

SACCE ------------------------------------------------------------

SCHPO ------------------------------------------------------------

TOXO ------------------------------------------------------------

PLAFA ------------------------------------------------------------

AGRO IEDKKAVAQAWFQHL----------------RDTIVASFETLEDELTGPLSDQEP----- 52

CAULO LDTKKAAARAWFESL----------------RDQICAAFEQLEDEAPADLYPGAP----- 45

RICPR NTENKEITSNWFTNL----------------RDLLCKEFEKIEEKY-AQIKGLKP----- 39

Wolb ---------MKEQ------------------KIQAFEWFRALRDRVVESFLLIEGQSPTD 33

wMel -------------------------------------MI--------ESFLLIEKQSSAE 15

wAna -----------MEKQ----------------KTQAFKWFCALRDKIIESFLLIEKQSSAE 33

RALSO -------------MD----------------TQAVRAYLLDLQDRITTAVGTLDG----- 26

ESCC -----------MKPD----------------AHQVKQFLLNLQDTICQQLTAVDG----- 28

SYNC NGTAAPALELPPEDS----------------RARARAMVMGLQDEICAGLESLDGE---- 80

SYNE N------HSLPPADA----------------KQRVSQFMQTLQDEICQGLEALDGK---- 45

CHLRE R------PGIDTEDS---------------MRARFEKVIRNAQDSICNAISEIDG----- 83

ARATH RDSDD-VTPSSSSSS---------------VRARFETMIRAAQDSVCDAIEAIEGG---- 105

Tobaco RESDMGSNVTSNSSS---------------VRGRFEKMRREAQDSVCLAIEKADGG---- 115

Maize-1 SEPPPTFLREDGHGSGS-------------VRARFESMIRRVQGEVCAALEEADGGG--- 111

Maize-2 LLSAVAIEKETPESELPPTFLREDGRGAGSVRARFESMIRRVQGEVCAALEEADGGG--- 132

CYAME REEDLQSGPLTTEHQRHIQGVPPVH---PRMRANFENMIRAAQNSICLALENVDGSG--- 127

MUS DELARRCSTFMSSPVTELRELRRRP---EDMKTKMELMIMETQAQVCRALAQVDGV---- 73

HOMO DELAHRCSSFMAPPVTDLGELRRRP---GDMKTKMELLILETQAQVCQALAQVDGG---- 173

Ciliate -------KKAKKTHVSKEEALER--------RKKFEQFVLDLQNHFVQTLQKYETK---- 53

SACCE ---MPAPQDPRNLPI----------------RQQMEALIRRKQAEITQGLESIDT----- 36

SCHPO ------MSDITVEPI----------------GKQMEKLILDVQQEIVAGLEAVDG----- 33

TOXO --MDSQKKQVYHPDKA--------------FRERWEAMLRRYQTAICDALASVDG----- 39

PLAFA ----MKDEIAPNEYF----------------RNLWENLLKSEQNNICSLIESLDN----- 35

.

AGRO GRFVQKDWLRDN---GEGGGGK-MSMMEGRVFEKVGVHTSTVYGEFSPEFRKQIPG---- 104

CAULO GRFAKKAWDRP-----AGGGGV-MGMMHGRLFEKVGVHVSTVFGTFTPEMAKTMPG---- 95

RICPR AKFVRTSWKRN-----GGGCGI-MSLMKGEVFEKVGVNISTVFGEFSQEFRSEILG---- 89

Wolb PKIEKRKWDR------PGGGGGESTIIYGDVFEKVGVNISKVYGKFADSVVNEIPG---- 83

wMel PKIEKRKWDR------PGGGGGESTIIFGNVFEKVGVNVSKVHGKFADSAINEIPG---- 65

wAna PKIEKRKWDR------PGGGGGESTIIFGNVFEKVGVNVSKVHGKFADSAINEIPG---- 83

RALSO GTFVTDTWDKPPT-ERLRGSGRTCILENGAVLERGGVGFSHVMGDTLPPSATANRP---- 81

ESCC AEFVEDSWQR-----EAGGGGRSRVLRNGGVFEQAGVNFSHVHGEAMPASATAHRP---- 79

SYNC GTFVEESWER-----PEGGGGRSRVMREGLVFEQGGVNFSEVQGQELPPSILKQRP---- 131

SYNE GKFQEDSWQR-----EEGGGGRSRVLADGDFLEQGGVNFSEVWGKSLPPSILKQRP---- 96

CHLRE KPFHQDAWTR-----PGGGGGISRVLQDGNVWEKAGVNVSVVYGTMPPEAYRAATG-NAE 137

ARATH PKFKEDVWSR-----PGGGGGISRVLQDGNVFEKAGVNVSVVYGVMPPEAYRAAKG-SAS 159

Tobaco AKFKEDVWSR-----PGGGGGHSSVLQDGAVFEKAGVNVSVVYGVMPPEAYRAARP-TDN 169

Maize-1 ARFVEDVWSR-----PCGGGGINPVLQDGRVFEKAGVNVSVVYGVIPPDAYRDGKGEADK 166

Maize-2 ARFVEDVWSR-----PGGGGGISRVLQDGRVFEKAGVNVSVVYGVMPPDAYRAAKGEAGK 187

CYAME KKFREDAWVRG----ADGGGGISRVLQDGHVFEKAGVNVSIVYGTMPPEALQAASAGAVA 183

MUS ADFTVDRWER-----KEGGGGITCVLQDGRVFEKAGVSISVVHGNLSEEAANQMRGRGKT 128

HOMO ANFSVDRWER-----KEGGGGISCVLQDGCVFEKAGVSISVVHGNLSEEAAKQMRSRGKV 228

Ciliate AVFLRDEWQR----KDGHGGGVTCVLQDGNFFEKAGVNTSKISFPFNHGIAASMKERGKV 109

SACCE VKFHADTWTRGN----DGGGGTSMVIQDGTTFEKGGVNVSVVYGQLSPAAVSAMKADHKN 92

SCHPO QKFFQDKWTKG-----EGGYGISCVIQDGNVFEKGGVNTSIVQGKLNQDAVQRMRANHEG 88

TOXO GSFCEDMWTRG----RSGGGGCSRVLQDSTVLEKGGVNVSAVHGTLPPDAVTKMTCHGHH 95

PLAFA KKFQEEVWYRKSGKNKNLGGGITRILEDGNIFEKCAVNYSCIYGAIDKESAKQMCVNHYN 95

: * : * * : . *: .* * :

AGRO ------------------------------AEEDPRFWASGLSLIAHPVNPNVPAVHMNT 134

CAULO ------------------------------AAEDPRFFATGISLIAHMTNPRVPAVHMNT 125

RICPR ------------------------------AELDGKFFATGISVVAHLKSPLIPAMHFNT 119

Wolb -----------------------------ASESNGEFWASGISLVSHMQSPLVPAAHMNT 114

wMel -----------------------------ASESNGEFWASGISLVSHMQSPLIPAAHMNT 96

wAna -----------------------------ASESNGEFWASGISLVSHMQSPLIPAAHMNT 114

RALSO ------------------------------ELAGRGFEAMGVSLVFHPRNPYAPTVHMNV 111

ESCC ------------------------------ELAGRSFEAMGVSLVVHPHNPYVPTSHANV 109

SYNC ------------------------------EAKGHPWFATGTSMVLHPHNPYIPTVHLNY 161

SYNE ------------------------------EAEGHEFYATGTSMVLHPKNPYIPTVHLNY 126

CHLRE KLKNKGDG------------------------GRVPFFAAGISSVMHPRNPHCPTMHFNY 173

ARATH -DQKPGP---------------------------VPFFAAGVSSVLHPKNPFAPTLHFNY 191

Tobaco GNVKPGP---------------------------IPFFAAGVSSVLHPKNPFAPTLHFNY 202

Maize-1 NGAAAGDG---------------------HKPGPVPFFAGGISSVLHPKNPFAPTLHFNY 205

Maize-2 NEAAADG----------------------HKPGPVPFFAAGISSVLHPKNPFAPTLHFNY 225

CYAME RNAGYARD------------------------ERVPFFAAGISSVIHPWNPHVPTMHFNY 219

MUS LKT---------------------------KDSKLPFTAMGVSSVIHPKNPYAPTMHFNY 161

HOMO LKT---------------------------KDGKLPFCAMGVSSVIHPKNPHAPTIHFNY 261

Ciliate FTDDEIKR--------------------------YNIYANGISLVIHPINPFAPTVHANY 143

SACCE LRLPEDPKTGLP------------------VTDGVKFFACGLSMVIHPVNPHAPTTHLNY 134

SCHPO IDR---------------------------TAKELPFFAAGISMVIHPRNPMAPTTHLNY 121

TOXO DLPAADG-------------------------DGLQFYAAGLSMVIHPRNPMAPTVHLNY 130

PLAFA KEFINTTKICHSDDINILISRVLNSHNIRIINEKYKFYASGISIIAHPVNPNVPTVHMNF 155

* * * : * .* *: * *

AGRO RMVVTT-----------------------------------------SHWFGGGADLTPV 153

CAULO RFIATT-----------------------------------------KSWFGGGGDLTPL 144

RICPR RYIETS-----------------------------------------KNWFGGGGDLTPF 138

Wolb RLIYTS-----------------------------------------KQWFGGGMDFTPT 133

wMel RLIYTS-----------------------------------------KQWFGGGMDFTPI 115

wAna RLIYTS-----------------------------------------KQWFGGGMDFTPI 133

RALSO RCFVAQRPDA-----------------------------------EPVWWFGGGMDLTPY 136

ESCC RFFIAEKPGA-----------------------------------DPVWWFGGGFDLTPF 134

SYNC RYFEAG----------------------------------------PVWWFGGGADLTPY 181

SYNE RYFEAG----------------------------------------PVWWFGGGADLTPY 146

CHLRE RYFETEEWNGIP----------------------------------GQWWFGGGTDITPS 199

ARATH RYFETDAPKDVPGA-------------------------------PRQWWFGGGTDFTPA 220

Tobaco RYFETDAPKDAPGA-------------------------------PRQWWFGGGTDFTPA 231

Maize-1 RYFETDAPKGAPG-------------------------------APRQWWFGGGTDLTPS 234

Maize-2 RYFETDAPKDAPG-------------------------------APRQWWFGGGTDLTPS 254

CYAME RYFETDK---------------------------------------GMWWMGGGTDLTPS 240

MUS RYFEVEEADG-----------------------------------NTHWWFGGGCDLTPR 186

HOMO RYFEVEEADG-----------------------------------HKQWWFGGGCDLTPT 286

Ciliate RWMELYDRETNQ---------------------------------TVDCWFGGGGDLTPH 170

SACCE RYFETWNQDGTP----------------------------------QTWWFGGGADLTPS 160

SCHPO RYFELVNSDGK-----------------------------------KIWWFGGGADLTPS 146

TOXO RFFQIFRRAPSERSGTNE-----------------CGGEEATEGESLLWWFGGGADLSPS 173

PLAFA RFFQIFIKTGKKKKKYNNLNNVNNNNLNNNLNNNFVKNKIDNNYKSIKHWFGGGCDLSPC 215

* . *:*** *::*

AGRO LGRRRTQQDPDTQLFHRAFEITCNRHPIADYPRYKSWCDEYFFLKHRDEPRGTGGIFFDW 213

CAULO LGYQRQQDFPDAIDFHAAYKRACDKYDPEWHPKYKAWCDEYFFLPHRNEPRGIGGIFYDH 204

RICPR YPEEN-----ETAKFHTAFKEACDKYDSSYYPKFKKQCDEYFYLRHRKEPRGVGGIFYDY 193

Wolb YKNEE-----DYKYIHESIKTTCDKFDAEYYLKFKEQCDNYFFLQHRKEPRGIGGIFYDN 188

wMel YKNEE-----DCKYIHESIKMTCDRFDTGYYPKFKEQCDNYFFLQHRKEPRGIGGIFYDN 170

wAna YRNEE-----DCKYIHESIKMICDRFDTGYYPKFKEQCDNYFFLQHRKEPRGIGGIFYDN 188

RALSO YGFAE-----DAAHFHRTCKQALEPFGEELYPRFKQWCDDYFYLKHRKEARGVGGIFFDD 191

ESCC YGFEE-----DAIHWHRTARDLCLPFGEDVYPRYKKWCDEYFYLKHRNEQRGIGGLFFDD 189

SYNC YPFLE-----DARHFHRTHQAACDSVHPDLHKVFKPWCDEYFFLKHRGETRGVGGIFYDY 236

SYNE YPFAE-----DAAHFHHTLKNACDQTHGEFYPVFKRWCDEYFYLKHRQEMRGIGGIFFDY 201

CHLRE YVVPE-----DMKHFHGTYKAVCDRHDPAYYEKFRTWCDEYFLIKHRGERRGLGGIFFDD 254

ARATH YIFEE-----DVKHFHSIQKQACDKFDPSFYPRFKKWCDDYFYIKHRDERRGLGGIFFDD 275

Tobaco YIFEE-----DVKHFHSVQKAACDKFDASFYPRFKKWCVDYFYIKHRDERRGLGGIFFDD 286

Maize-1 YIIEE-----DVKHFHSVQKQACDKFDPSFHPRFKKWCDDYFYIKHRNERRGLGGIFFDD 289

Maize-2 YIIEE-----DVKHFHSVQKQACDKFDPSFHPRFKKWCDDYFYIKHRNERRGLGGIFFDD 309

CYAME YLYVE-----DAQHFHGVLKSVCDRHDAAFYQRFKKWCDDYFLIRHRGERRGIGGVFFDD 295

MUS YLNQE-----DAVHFHRTLKEACDQHGPDIYPKFKKWCDDYFFIVHRGERRGIGGIFFDD 241

HOMO YLNQE-----DAVHFHRTLKEACDQHGPDLYPKFKKWCDDYFFIAHRGERRGIGGIFFDD 341

Ciliate YLFEE-----DCKEFHQGFKNACDPYGQDLYKKLKKECDDYFFIKFRNERRGIGGIFYDD 225

SACCE YLYEE-----DGQLFHQLHKDALDKHDTALYPRFKKWCDEYFYITHRKETRGIGGIFFDD 215

SCHPO ILFEE-----DGKHFHKLHKEACDRHDPTFYPRFKKWADEYFLIKHRKETRGIGGIFFDD 201

TOXO HVFEE-----DCVFFHEKLREQCDRRDPLYYARFKRWCDAYFRNHHRNEGRGIGGIFFDD 228

PLAFA YIFPD-----LFTEFHNSFKLVCDKYNHLFYRHFKIWCDLYFRIKHRNINRGIGGIFFDN 270

* . : : . ** .* ** **:*:*

AGRO LHPDE-------------------------------------------EKGGWDANFTFV 230

CAULO HDS-----------------------------------------------GDWARDFAFT 217

RICPR LNS-----------------------------------------------GNFEQDFAFT 206

Wolb LNS-----------------------------------------------GRWENDFEFT 201

wMel LSS-----------------------------------------------SNWENDFEFT 183

wAna LSS-----------------------------------------------GNWENDFEFT 201

RALSO FA-----------------------------------------------ELGFERSFEMM 204

ESCC LN-----------------------------------------------TPDFDRCFAFM 202

SYNC QDSSGVLYKGQDPSGPAAGVSAQL---------------------G-ARPLGWEQLFALG 274

SYNE QDGNAPLYRGPDPNGPAAQYSNQL---------------------APIEPLGWEDLFSFA 240

CHLRE LND-----------------------------------------------RNPEDILKFS 267

ARATH LND-----------------------------------------------YDQEMLLSFA 288

Tobaco FND-----------------------------------------------YDQEMLLSFS 299

Maize-1 LND-----------------------------------------------YDQEMLLNFA 302

Maize-2 LND-----------------------------------------------YDQEMLLNFA 322

CYAME LND-----------------------------------------------RPAEELFKFS 308

MUS LDSP-----------------------------------------------SKEEAFRFV 254

HOMO LDSP-----------------------------------------------SKEEVFRFV 354

Ciliate FLIED----------------------------------------------SWDKTFEFA 239

SACCE YDE-----------------------------------------------RDPQEILKMV 228

SCHPO LSE-----------------------------------------------KDPQELFAFV 214

TOXO LNEN--------------------------------------------MTVSPEKFFAFA 244

PLAFA LLNNIIKNKKVIRSGKLKDINKKNGNDPTINNNNNNNKDCKCYSCNNIMDKSYRMIYFFI 330

:

AGRO QDVGRAFNLVYPKIVRANFNQNWTEEDRDEQLIRRGRYVEFNLLYDRGTIFGLKT----- 285

CAULO QDVGRAFLEIYPTLVRRRMGEAWTADEREQQLIQRGRYVEFNLLYDRGTMFGLKT----- 272

RICPR KDIGKALLSVYPEIVRSKLFLPWTAEQKEYQLIRRGRYVEFNLLYDRGTKFGLMT----- 261

Wolb KAVGETFLKIYLHIIRQHMRKPWTKEQRETQLIKRGRYVEFNLLYDRGTKFGLMT----- 256

wMel KAVGEAFLEIYLHIIRKHIQKSWTKEQRENQLIKRGRYVEFNLLYDRGTRFGLMT----- 238

wAna KAVGEAFLEIYLHIIRKHIQKSWTKEQRENQLIKRGRYVEFNLLYDRGTRFGLMT----- 256

RALSO RAVGDALLPAWLPIAEQRHATPYGERERAFQAYRRGRYVEFNLVFDRGTLFGLQS----- 259

ESCC QAVGKGYTDAYLPIVERRKAMAYGERERNFQLYRRGRYVEFNLVWDRGTLFGLQT----- 257

SYNC QANGRAFLPSYAPIVEKRHPMAYGDRERQFQLYRRGRYVEFNLVWDRGTIFGLQT----- 329

SYNE QRCGRAFLPAYSPIVEKRRNTEYGDRQRQFQLYRRGRYVEFNLVYDRGTIFGLQT----- 295

CHLRE TDAVNNVVEAYCPIIKKHMNDPYTPEEKEWQQIRRGRYVEFNLVYDRGTTFGLKT----- 322

ARATH TECANSVVPAYIPIVEKRKDMEFTEQHKAWQQLRRGRYVEFNLVYDRGTTFGLKT----- 343

Tobaco TECANSVIPAYIPIVEKRKDTPFTDKHKAWQQLRRGRYVEFNLVYDRGTTFGLKT----- 354

Maize-1 TECADSVLPAYIPIIERRKNTPFNEEHRAWQQLRRGRYVEFNLVYDRGTTFGLKT----- 357

Maize-2 TECADSVLPAYIPIIERRKDTPFNEEHKEWQQLRRGRYVEFNLVYDRGTTFGLKT----- 377

CYAME SDMVGHVVEAYVPIVERRKHMPYTEEQKRWQQLRRGRYVEFNLVYDRGTVFGLKT----- 363

MUS KTCAEAVVPSYVPIVKKHCDDSYTPRDKLWQQLRRGRYVEFNLLYDRGTKFGLFTP---- 310

HOMO QSCARAVVPSYIPLVKKHCDDSFTPQEKLWQQLRRGRYVEFNLLYDRGTKFGLFTP---- 410

Ciliate QKAGWATLNSYNTILERRKDTQYNEKNMEWKQIRRGRYVEFNLVYDRGTKFGLFTP---- 295

SACCE EDCFDAFLPSYLTIVKRRKDMPYTKEEQQWQAIRRGRYVEFNLIYDRGTQFGLRTP---- 284

SCHPO KDCAHTFLPAYVPIMEKRKNMEFTEDDKEFQLIRRGYYAEFNVMYDRGTWFGLQAP---- 270

TOXO EDGLQTFIDAYIPILIKRKDQPFTESQKIWQQIRRGRYVEFNLVHDRGTKFGFQVP---- 300

PLAFA QECIINFRKSYLHILLETVNFKYDDNMLKWQRVCRGRYVEFNLLYDRGTKFGIELNRYKI 390

: : : : ** *.***:: **** **:

AGRO -----------------------------------GGNVESILSSLPPVVRWP------- 303

CAULO -----------------------------------GGNVESILSSMPPAVKWP------- 290

RICPR -----------------------------------DGNVEAILMSLPPVVKFN------- 279

Wolb -----------------------------------DGNPDAIMMSMPPLVKWM------- 274

wMel -----------------------------------DGNPDAIMMSMPPLVKWL------- 256

wAna -----------------------------------DGNPDAIMMSMPPLVKWL------- 274

RALSO -----------------------------------GGRTESILMSMPPVANWRYDWQPEP 284

ESCC -----------------------------------GGRTESILMSMPPLVRWEYDYQPKD 282

SYNC -----------------------------------NGRTESILMSLPPLVRWEYGYKAEA 354

SYNE -----------------------------------NGRTESILMSLPPLVRWQYCYSPEA 320

CHLRE -----------------------------------GGRIESILMSMPQTASWLYDHQPKA 347

ARATH -----------------------------------GGRIESILVSLPLSARWEYDHKPEE 368

Tobaco -----------------------------------GGRIESILVSLPLTARWEYDHKPEE 379

Maize-1 -----------------------------------GGRIESILVSLPLTARWQYDHKPEE 382

Maize-2 -----------------------------------GGRIESILVSLPLTARWQYDHKPEE 402

CYAME -----------------------------------GGRIESILMSLPLTARWEYDHQPAE 388

MUS -----------------------------------GSRIESILMSLPLTARWEYMHSPPE 335

HOMO -----------------------------------GSRIESILMSLPLTARWEYMHSPSE 435

Ciliate -----------------------------------DARIESILMSLPHVAKWEYMYKIEE 320

SACCE -----------------------------------GSRVESILMSLPEHASWLYNHHPAP 309

SCHPO -----------------------------------EPRVESILMTLPLHASWRYKYEPKQ 295

TOXO -----------------------------------GSRIESILISLPLTARWEYQFKIEK 325

PLAFA YRRKKKQKKLENYSSTNFIKDEIFDEQVSDYLSDEHEKIDNVFSSLPLKCDFQYKYKIEK 450

. : :: ::* :

AGRO ---------------------------------------

CAULO ---------------------------------------

RICPR ---------------------------------------

Wolb ---------------------------------------

wMel ---------------------------------------

wAna ---------------------------------------

RALSO GSPEAALYTDFLPARDWV--------------------- 302

ESCC GSPEAALS-EFIKVRDWV--------------------- 299

SYNC GSREALLTELFTKPQDWLGDASLEDRCRPHGAIN----- 388

SYNE GSPEAELTEKFLVPQDWVNS------------------- 340

CHLRE GSPEAELLDACRNPRVWV--------------------- 365

ARATH GTEEWKLLDACINPKEWI--------------------- 386

Tobaco GTEEWKLLDACINPKEWI--------------------- 397

Maize-1 GTEEWKLLEACINPKDWI--------------------- 400

Maize-2 GTEEWKLLDACINPKDWI--------------------- 420

CYAME GTPEWDLLDACRHPREWV--------------------- 406

MUS NSKEAEILEVLRHPKDWVH-------------------- 354

HOMO NSKEAEILEVLRHPRDWVR-------------------- 454

Ciliate GSEEEKMQKVLINPVDWI--------------------- 338

SACCE GSREAKLLEVTTKPREWVK-------------------- 328

SCHPO -ERHKALLKVTHTPIEWC--------------------- 312

TOXO GSKEEEAQRVFVEPRDWLPIDNAHLEGFSWPTETGARRC 364

PLAFA YSREYETLEILKYPKKWVDY------------------- 470

**CLUSTAL X (1.83) multiple sequence alignment for FC (8)**

**30 taxa/251 characters from underlined conserved regions are used for phylogeny analysis**

**Note: Cysteine residues involved in binding [Fe-S] cluster is labeled in red color in human FC.**

**Legend for abbreviations used in the alignment:**

**TOXO**  913aa DQ029346 ferrochelatase Toxoplasma gondii apicomplexa/Alveolates

PLAFA CAC82988 ferrochelatase [Plasmodium falciparum] 350aa apicomplexan /Alveolates

**RICPR** Q9ZC84 342aa Rickettsia prowazekii FERROCHELATASE Alpha-proteobacteria

**CAUCR** NP_422556 347aa ferrochelatase [Caulobacter crescentus CB15] Alpha-proteobacteria

**RHOCA** JC4752 351aa ferrochelatase Rhodobacter capsulatus alpha-proteobacteria

**Agro** 368aa NP_356851 Ferrochelatase [Agrobacterium tumefaciens] alpha-proteobacteria

**Wolb** AAW71307 340aa ferrochelatase [Wolbachia endosymbiont strain TRS of Brugia malayi] alpha-proteobacteria

**wAna** ZP_00373515 315aa ferrochelatase [Wolbachia endosymbiont of Drosophila ananassae] alpha-proteobacteria

**wWill** ZP_01315120 315aa ferrochelatase [Wolbachia endosymbiont of Drosophila willistoni TSC#14030-0811.24] alpha-proteobacteria

**wMel** NP_966898 315aa ferrochelatase [Wolbachia endosymbiont of Drosophila melanogaster] alpha-proteobacteria

**NEIME** Q9K097 336aa Neisseria meningitidis serogroup B Ferrochelatase Beta-proteobacteria

**RALEU** 371aa YP_295260 ferrochelatase [Ralstonia eutropha JMP134] Beta-proteobacteria

**YERMO** 320aa ZP_00826183 ferrochelatase [Yersinia mollaretii ATCC 43969] Gamma-proteobacteria

**ECOLI** P23871 320aa Escherichia coli Ferrochelatase Gamma-proteobacteria

**HELPY** Q9ZKD4 335aa Helicobacter pylori J99 FERROCHELATASE Epsilon-proteobacteria

**BACSU** NP_388894 310aa ferrochelatase [Bacillus subtilis] Firmicutes/G+ bacteria

**SYNE** NP_442453 387aa ferrochelatase [Synechocystis sp. PCC 6803] cyanobacteria

**SYNC** 391aa NP_897838 Ferrochelatase [Synechococcus sp. WH 8102]cyanobacteria

**CYAME*** CMS035C 413aa Ferrochelatase retrieved from http://merolae.biol.s.u-tokyo.ac.jp/ Cyanidioschyzon merolae Genome Project, Cyanidioschyzon merolae red algae

**THAPS** EED96635 506aa Ferrochelatase grail.14.169.1 retrieved from http://genome.jgi-psf.org/thaps1/thaps1.home.html thalassiosira pseudonana Diatoms/Heterokonts

**Cucumber1**  514aa T10246 ferrochelatase I (EC 4.99.1.1) - [Cucumis sativus] Plants

**Cucumber2** 522aa BAB20760 ferrochelatase II [Cucumis sativus] Plants

**ARATH1** P42043 Arabidopsis thaliana Ferrochelatase I, 466aa chloroplast/mitochondrial precursor green plants

**ARATH2** O04921 Arabidopsis thaliana Ferrochelatase II, 512aa chloroplast precursor green plants

**CHLRE** AAK16728 493aa ferrochelatase [Chlamydomonas reinhardtii] Green algae/Chlorophyta

**HOMO** XP_008784 423aa ferrochelatase [Homo sapiens] Animals

**MUS** NP_032024 422aa ferrochelatase [Mus musculus] Animals

**SACCE** IBBYFC 393aa ferrochelatase precursor - yeast (Saccharomyces cerevisiae) fungi

**SCHPO** T41302 384aa ferrochelatase precursor - fission yeast (Schizosaccharomyces pombe) fungi

**Ciliate** EAR97552 393aa Ferrochelatase retrieved from http://tigrblast.tigr.org/tgi/TIGR "Tetrahymena thermophila genome sequencing project" Ciliates/Alveolates

PLAFA ------------------------------------------------------------

TOXO MRHDVLTRLCGALETAAGTTSQTKCFWRDRSGVFRFLCSQASLGAERLPTPAFTKTRDAD 60

RHOCA ------------------------------------------------------------

AGRO ------------------------------------------------------------

ECOLI ------------------------------------------------------------

YERMO ------------------------------------------------------------

NEIME ------------------------------------------------------------

RALEU ------------------------------------------------------------

HELPY ------------------------------------------------------------

CYAME ------------------------------------------------------------

THAPS ------------------------------------------------------------

RICPR ------------------------------------------------------------

Wolb ------------------------------------------------------------

wWill ------------------------------------------------------------

wMel ------------------------------------------------------------

wAna ------------------------------------------------------------

CAUCR ------------------------------------------------------------

SYNE ------------------------------------------------------------

SYNC ------------------------------------------------------------

Cucumber2 ------------------------------------------------------------

ARATH2 ------------------------------------------------------------

CHLRE ------------------------------------------------------------

Cucumber1 ------------------------------------------------------------

ARATH1 ------------------------------------------------------------

HOMO ------------------------------------------------------------

MUS ------------------------------------------------------------

SACCE ------------------------------------------------------------

SCHPO ------------------------------------------------------------

Ciliate ------------------------------------------------------------

BACSU ------------------------------------------------------------

PLAFA ------------------------------------------------------------

TOXO APVVATSRVWTRQSEPGESGLLQWSRNTGNTPSCRNVYSADTSTCSISRLSPAARSLDSE 120

RHOCA ------------------------------------------------------------

AGRO ------------------------------------------------------------

ECOLI ------------------------------------------------------------

YERMO ------------------------------------------------------------

NEIME ------------------------------------------------------------

RALEU ------------------------------------------------------------

HELPY ------------------------------------------------------------

CYAME ------------------------------------------------------------

THAPS ------------------------------------------------------------

RICPR ------------------------------------------------------------

Wolb ------------------------------------------------------------

wWill ------------------------------------------------------------

wMel ------------------------------------------------------------

wAna ------------------------------------------------------------

CAUCR ------------------------------------------------------------

SYNE ------------------------------------------------------------

SYNC ------------------------------------------------------------

Cucumber2 ------------------------------------------------------------

ARATH2 ------------------------------------------------------------

CHLRE ------------------------------------------------------------

Cucumber1 ------------------------------------------------------------

ARATH1 ------------------------------------------------------------

HOMO ------------------------------------------------------------

MUS ------------------------------------------------------------

SACCE ------------------------------------------------------------

SCHPO ------------------------------------------------------------

Ciliate ------------------------------------------------------------

BACSU ------------------------------------------------------------

PLAFA ------------------------------------------------------------

TOXO DQYATKKKGLRGRRSECDHEPPSTREWSAQTVPEAFCLLGSGGSLAPPSFGPFFSAGGRR 180

RHOCA ------------------------------------------------------------

AGRO ------------------------------------------------------------

ECOLI ------------------------------------------------------------

YERMO ------------------------------------------------------------

NEIME ------------------------------------------------------------

RALEU ------------------------------------------------------------

HELPY ------------------------------------------------------------

CYAME ------------------------------------------------------------

THAPS ------------------------------------------------------------

RICPR ------------------------------------------------------------

Wolb ------------------------------------------------------------

wWill ------------------------------------------------------------

wMel ------------------------------------------------------------

wAna ------------------------------------------------------------

CAUCR ------------------------------------------------------------

SYNE ------------------------------------------------------------

SYNC ------------------------------------------------------------

Cucumber2 ------------------------------------------------------------

ARATH2 ------------------------------------------------------------

CHLRE ------------------------------------------------------------

Cucumber1 ------------------------------------------------------------

ARATH1 ------------------------------------------------------------

HOMO ------------------------------------------------------------

MUS ------------------------------------------------------------

SACCE ------------------------------------------------------------

SCHPO ------------------------------------------------------------

Ciliate ------------------------------------------------------------

BACSU ------------------------------------------------------------

PLAFA ------------------------------------------------------------

TOXO FLHSRLRVGFQKSDQEKDKHNVRVFSTHMGRQRAEMEKTRRREGLVEEGFGEAEVTGRRE 240

RHOCA ------------------------------------------------------------

AGRO ------------------------------------------------------------

ECOLI ------------------------------------------------------------

YERMO ------------------------------------------------------------

NEIME ------------------------------------------------------------

RALEU ------------------------------------------------------------

HELPY ------------------------------------------------------------

CYAME ----------------------------------------------------------MF 2

THAPS ---------------------------------------MSSIAIIVLISILETALPFGL 21

RICPR ------------------------------------------------------------

Wolb ------------------------------------------------------------

wWill ------------------------------------------------------------

wMel ------------------------------------------------------------

wAna ------------------------------------------------------------

CAUCR ------------------------------------------------------------

SYNE ------------------------------------------------------------

SYNC ------------------------------------------------------------

Cucumber2 ------------------------MDSAIQASSSPASSSAFRSPCLTSASQNCKFPLPTS 36

ARATH2 ------------------------MNCPAMTASPSSSS--------SSSYSTFRPPPPLL 28

CHLRE ------------------------MASFGLMQRTVHCP-------------------QLV 17

Cucumber1 ---------------------------MDAASSSLALSNIKLHGSTNTLNSDQRISSLCS 33

ARATH1 ---------------------------MQATALSSGFN-------PLTKRKDHRFPRSCS 26

HOMO ------------------------------------------------------------

MUS ------------------------------------------------------------

SACCE ------------------------------------------------------------

SCHPO ------------------------------------------------------------

Ciliate ------------------------------------------------------------

BACSU ------------------------------------------------------------

PLAFA --------------------------------------------------------MDVQ 4

TOXO SVRASERQERSDGTEDALWRKEADSLSEEARKLQCLYHSRSSYLWDEKQTLRPRLPAWLR 300

RHOCA -----------------------------------------------------------M 1

AGRO --------------------------------------MECSGLYPRRNRSSHNQSEFQV 22

ECOLI ------------------------------------------------------------

YERMO ------------------------------------------------------------

NEIME ------------------------------------------------------------

RALEU ------------------------------------------------------------

HELPY ------------------------------------------------------------

CYAME VVVPGNAWRLPRPRGGLHRRPTWRLSLIHGSEDGQHALTSSGTARNAASPATSTTATATT 62

THAPS GVSAFSSSISPRVASPSSLSRPPQHVHASAPPSSTSSYRSATTTKLAAATALDLSDESIT 81

RICPR ------------------------------------------------------------

Wolb ------------------------------------------------------------

wWill ------------------------------------------------------------

wMel ------------------------------------------------------------

wAna ------------------------------------------------------------

CAUCR ------------------------------------------------------------

SYNE ------------------------------------------------------------

SYNC ------------------------------------------------------------

Cucumber2 RVVGSKRHRAFRLHMDACPTKCHVVSRYSFE----------LPDSQSIFSKKSINKFFPP 86

ARATH2 PQLSNDSQRSVVMHCTRLPFEAFAATSSNRL----------LGKHSLPLRAALVTSNPLN 78

CHLRE EERCSPVAGCSGRGLPVIQRQRRGVCSATNG----------VQRGRVLRRTAASTDVVSF 67

Cucumber1 LPKSRVTFSCKTSGNLQVRDRSTGLVVSCSSSNGDRDVIQGLHLSGPIEKKSRLGQACCS 93

ARATH1 QRNSLSLIQCDIK------ERSFGESMTITN-------------RGLSFKTNVFEQARSV 67

HOMO --------------MRSLGANMAAALRAAGVLLRDPLASSSWRVCQPWRWKSGAAAAAVT 46

MUS --------------MLSASANMAAALRAAGALLREPLVHGSSRACQPWRCQSG-AAVAAT 45

SACCE --------------------MLSRTIRTQGSFLRR------------------------S 16

SCHPO -----------------------MSVSSYSS---------------------------DA 10

Ciliate ------------------------------------------------------------

BACSU ------------------------------------------------------------

PLAFA DFLNCNKLKISKEKISNLNKSKIGILITNLGSPEKLTYWSLYKYLSEFLTDPRVVKLN-- 62

TOXO QNCDEDREGEKRVKPRKADGAAVCVILVNLGSPSAPTYTELWKYLNQFLGDPRVVELP-- 358

RHOCA TIANRILPHAP-ADHPPVPVPRVGVLLANLGTPDATDYWSMRRYLNEFLSDRRVIDYP-- 58

AGRO SFMATELSALP-ANHPRVTFGKVGVLLVNLGTPDGTDYWPMRRYLAEFLSDKRVIEWS-- 79

ECOLI -----------------MRQTKTGILLANLGTPDAPTPEAVKRYLKQFLSDRRVVDTS-- 41

YERMO -----------------MMQTKLGVLMVNLGTPDAPTSQAVKRYLAEFLSDRRVVDTS-- 41

NEIME -----MLPFFPEPSLSYTQQNRTAVLLLNLGTPDAPTAQAVRPYLKSFLTDRRVVELP-- 53

RALEU ------MTFSPEPAYQHGQAPRTAILLVNLGTPDAPTPKAVGRYLKEFLSDPRVVEIPR- 53

HELPY --MNLINEKLNNLENSATKSPKEAVVLLNMGGPN--SLYEVGVFLKNMFDDPFILTIKN- 55

CYAME ARVANRGTAGSASFLEPRFQGRTGLILVNIGTPASLNVADVRRYLREFLGDDRVVDIR-- 120

THAPS LPSWDKNDDTSTDTIAKLKDPKVGVLLLNLGGPE--TGEDVEGFLYNLFADPDIIRLPS- 138

RICPR ------------------MNKRIAIVLFNLGGPE--DIEYVKPFLFNLFYDKAIINLP-- 38

Wolb --------------------MKKAVVLLNLGGPD--SLSAVRPFLFNLFYDKRIINLPN- 37

wWill --------------------MKKAVILFNLGGPD--SLNAVRPFLFNLFYDRRIINLPN- 37

wMel --------------------MKKAVILFNLGGPD--SLNAVRPFLFNLFYDRRIINLPN- 37

wAna --------------------MKKAVILFNLGGPD--SLNAVRPFLFNLFYDRRIINLPN- 37

CAUCR ------------------MTQKLAVVLFNLGGPD--GPDAVRPFLFNLFRDPAIIGAP-- 38

SYNE -------------------MGRVGVLLLNLGGPE--KLEDVRPFLFNLFADPEIIRLP-- 37

SYNC -------------------MSRVGVVLLNLGGPE--RIQDVGPFLYNLFADPEIIRLP-- 37

Cucumber2 PRALVASNTQNTSAAPLIGEDKVGVLLLNLGGPE--TLDDVQPFLFNLFADPDIIRLPRL 144

ARATH2 ISSSSVISDAISSSSVITDDAKIGVLLLNLGGPE--TLDDVQPFLFNLFADPDIIRLPPV 136

CHLRE VDPNDIRKPAAAAAG-PAVD-KVGVLLLNLGGPE--KLDDVKPFLYNLFADPEIIRLPAA 123

Cucumber1 VGTFTVGEFALESQS-QAVDDKVGVLLLNLGGPE--TLDDVQPFLYNLFADPDIIRLPRL 150

ARATH1 TGDCSYDETSAKARSHVVAEDKIGVLLLNLGGPE--TLNDVQPFLYNLFADPDIIRLPRP 125

HOMO TETAQHAQGAKPQVQPQKRKPKTGILMLNMGGPE--TLGDVHDFLLRLFLDQDLMTLP-- 102

MUS TEKVHHAKTTKPQAQPERRKPKTGILMLNMGGPE--TLGEVQDFLQRLFLDRDLMTLP-- 101

SACCE QLTITRSFSVTFNMQNAQKRSPTGIVLMNMGGPS--KVEETYDFLYQLFADNDLIPISA- 73

SCHPO SSTVMDESPPNGVTKSVSGKGPTAVVMMNMGGPS--NLDEVGPFLERLFTDGDIIPLG-- 66

Ciliate ----MLSQIFSRTFSSSKQQAKTAVFMLNLGGPN--SLEEVSPFLERFFADSTVIRIP-- 52

BACSU -----------------MSRKKMGLLVMAYGTPY--KEEDIERYYTHIRRGR-------- 33

:.: * * : : .

PLAFA -RFLWLPILYTFVLPFR-SGKVLSKYKSIWIKDG-------------------------- 94

TOXO -SFLWSFIRYLFILPFR-SYASAQKYQSIWNFDPGFKLCASQSHVNKSSSLQSRVLGGAQ 416

RHOCA -IWKWQPLLQLIILSKR-PFTSGNNYRSIWNEER-------------------------- 90

AGRO -RLYWYPILYGIVLNKR-PQKVGKAYEEIWNHER-------------------------- 111

ECOLI -RLLWWPLLRGVILPLR-SPRVAKLYASVWME---------------------------- 71

YERMO -PLLWWPLLRGVILPIR-SPRVAKLYQSVWMEEG-------------------------- 73

NEIME -KWLWYPILHGLVLTLR-PKKSAHAYEKIWFK---------------------------- 83

RALEU --LAWLPLLYGVILPLR-ARASALKYESIWLREAHMTG---------------------- 88

HELPY --NFMRKMVGKMIVNSR-IEKSKKIYEKLGGK---------------------------- 84

CYAME -PKWLKTILLQVLLLTR-PAKSAEAYANIWDKETG------------------------- 153

THAPS ILSPLQSLVALLISKRR-APKSREAYDSIGGG---------------------------- 169

RICPR --NPLRYIIAKIISITR-EKKSQKIYSLIGSK---------------------------- 67

Wolb ---PFRFFLAKFISAKR-ENNARKIYEQIGGK---------------------------- 65

wWill ---PFRFLLAKFISAKR-ENTARKIYEEIGGK---------------------------- 65

wMel ---PFRFLLAKFISAKR-ENTARKIYEEIGGK---------------------------- 65

wAna ---PFRFLLAKFISAKR-ENTARKIYEEIGGK---------------------------- 65

CAUCR --ALIRYPLAALISTTR-EKSAKANYAIMGGG---------------------------- 67

SYNE -FPWLQKPLAWLISTLR-AKKSQANYAEIGGG---------------------------- 67

SYNC -SPALQKPLAWLISTLR-SGKSQEAYRSIGGG---------------------------- 67

Cucumber2 -FRFLQRPLARFISVLR-SPKSREGYASIGGG---------------------------- 174

ARATH2 -FQFLQKPLAQFISVAR-APKSKEGYASIGGG---------------------------- 166

CHLRE -AQFLQPLLATIISTLR-APKSAEGYEAIGGG---------------------------- 153

Cucumber1 -FRFLQEPLAKLISTYR-APKSKEGYASIGGG---------------------------- 180

ARATH1 -FQFLQGTIAKFISVVR-APKSKEGYAAIGGG---------------------------- 155

HOMO ----IQNKLAPFIAKRR-TPKIQEQYRRIGGG---------------------------- 129

MUS ----IQNKLAPFIAKRR-TPKIQEQYRRIGGG---------------------------- 128

SACCE ---KYQKTIAKYIAKFR-TPKIEKQYREIGGG---------------------------- 101

SCHPO ---YFQNSLGKFIAKRR-TPKVQNHYSDIGGG---------------------------- 94

Ciliate ------FGLGPKIGKLRGPAKVTKQYEAIGGR---------------------------- 78

BACSU ------KPEPEMLQDLK------DRYEAIGGI---------------------------- 53

: : * :

PLAFA -----------------SPLCVNTHNQCLALKKILNEKYN-------------------- 117

TOXO TEAVRQTQVQAVQPIPEEPCTVSTSAEVEARKAKDRRGGKNSIMYAERSAKDATQRAQAP 476

RHOCA ---------------DESPLMTITRDQVRKLRAAVETRYG-------------------- 115

AGRO ---------------NESYLRTYTRSQGELMAAALKDFP--------------------- 135

ECOLI ---------------GGSPLMVYSRQQQQALAQRLPEMP--------------------- 95

YERMO -----------------SPLLVYSRRQQKALAARMPDIP--------------------- 95

NEIME ---------------EGSPLEVYTARQAAALAKRMPDLI--------------------- 107

RALEU -----------------SPLLVYSERQAHALQRLLNQQGY-------------------- 111

HELPY -----------------SPLTPITFALTERLNELDP------------------------ 103

CYAME -----------------SPLLHYSQELATKIQKRVGNGY--------------------- 175

THAPS -----------------SPILQYTRAQADLMAESLKSRYG-------------------- 192

RICPR -----------------SYLIQETEKQKLAITEKLKEFIK-------------------- 90

Wolb -----------------SPILENTKMQAEALERELNRSVFCHPSSVTLGPRKENWIPVSR 108

wWill -----------------SPILENTKMQANASELKLNENRN-------------------- 88

wMel -----------------SPILENTKMQANASELKLNENRN-------------------- 88

wAna -----------------SPILENTKMQANALELKLNENRN-------------------- 88

CAUCR -----------------SPLLPETEKQARALEAALALAMPG------------------- 91

SYNE -----------------SPLLQITEAQASALTTRLERLGQ-------------------- 90

SYNC -----------------SPLRRITEQQARELQSLLRQRGL-------------------- 90

Cucumber2 -----------------SPLRKITDAQAEELKKALWQKDV-------------------- 197

ARATH2 -----------------SPLRHITDAQAEELRKCLWEKNV-------------------- 189

CHLRE -----------------SPLRRITDEQAEALAESLRAKGQ-------------------- 176

Cucumber1 -----------------SPLRKITDEQAQALKMALAEKNM-------------------- 203

ARATH1 -----------------SPLRKITDEQADAIKMSLQAKNI-------------------- 178

HOMO -----------------SPIKIWTSKQGEGMVKLLDELSPNT------------------ 154

MUS -----------------SPIKMWTSKQGEGMVKLLDELSPAT------------------ 153

SACCE -----------------SPIRKWSEYQATEVCKILDKTCPET------------------ 126

SCHPO -----------------SPILHWTRIQGSEMCKILDKKCPES------------------ 119

Ciliate -----------------SPIQDWTRKQGEKMVEKLDQISPDT------------------ 103

BACSU -----------------SPLAQITEQQAHNLEQHLNEIQD-------------------- 76

. :

PLAFA ------------------------------------------------------------

TOXO FSSLETEDKNTVYPVSGSSGFGSSRDSRIATSDTHQGERDFSGAPAPLVRITECLRRKVQ 536

RHOCA ------------------------------------------------------------

AGRO ------------------------------------------------------------

ECOLI ------------------------------------------------------------

YERMO ------------------------------------------------------------

NEIME ------------------------------------------------------------

RALEU ------------------------------------------------------------

HELPY ------------------------------------------------------------

CYAME ------------------------------------------------------------

THAPS ------------------------------------------------------------

RICPR ------------------------------------------------------------

Wolb IG---------------------------------------------------------- 110

wWill ------------------------------------------------------------

wMel ------------------------------------------------------------

wAna ------------------------------------------------------------

CAUCR ------------------------------------------------------------

SYNE ------------------------------------------------------------

SYNC ------------------------------------------------------------

Cucumber2 ------------------------------------------------------------

ARATH2 ------------------------------------------------------------

CHLRE ------------------------------------------------------------

Cucumber1 ------------------------------------------------------------

ARATH1 ------------------------------------------------------------

HOMO ------------------------------------------------------------

MUS ------------------------------------------------------------

SACCE ------------------------------------------------------------

SCHPO ------------------------------------------------------------

Ciliate ------------------------------------------------------------

BACSU ------------------------------------------------------------

PLAFA ------------------------------------------------------------

TOXO ARFDTLLLHLGERERRGKRDAPYDENCLERGDSSRHRGENLFSLDRDKRGEALSKAGARC 596

RHOCA ------------------------------------------------------------

AGRO ------------------------------------------------------------

ECOLI ------------------------------------------------------------

YERMO ------------------------------------------------------------

NEIME ------------------------------------------------------------

RALEU ------------------------------------------------------------

HELPY ------------------------------------------------------------

CYAME ------------------------------------------------------------

THAPS ------------------------------------------------------------

RICPR ------------------------------------------------------------

Wolb ------------------------------------------------------------

wWill ------------------------------------------------------------

wMel ------------------------------------------------------------

wAna ------------------------------------------------------------

CAUCR ------------------------------------------------------------

SYNE ------------------------------------------------------------

SYNC ------------------------------------------------------------

Cucumber2 ------------------------------------------------------------

ARATH2 ------------------------------------------------------------

CHLRE ------------------------------------------------------------

Cucumber1 ------------------------------------------------------------

ARATH1 ------------------------------------------------------------

HOMO ------------------------------------------------------------

MUS ------------------------------------------------------------

SACCE ------------------------------------------------------------

SCHPO ------------------------------------------------------------

Ciliate ------------------------------------------------------------

BACSU ------------------------------------------------------------

PLAFA -------------------------------------------------------NKVVI 122

TOXO PPGHAREQSKGDTREDPFLYPESGETQASVCSLAGNSDRHSFLERSDYDAARRLQPAVRV 656

RHOCA ------------------------------------------------------AGNVVV 121

AGRO --------------------------------------------------------NVVV 139

ECOLI -----------------------------------------------------------V 96

YERMO -----------------------------------------------------------V 96

NEIME -----------------------------------------------------------V 108

RALEU --------------------------------------------------------ELTV 115

HELPY --------------------------------------------------------SRFY 107

CYAME ----------------------------------------------------------AV 177

THAPS -------------------------------------------------------IEAKT 197

RICPR -------------------------------------------------------EDFII 95

Wolb ----------------------------------------------------MTSKLTKV 118

wWill -------------------------------------------------------HVHKV 93

wMel -------------------------------------------------------HVHKV 93

wAna -------------------------------------------------------HVHKV 93

CAUCR -------------------------------------------------------VEAKC 96

SYNE --------------------------------------------------------DAKV 94

SYNC --------------------------------------------------------DATT 94

Cucumber2 --------------------------------------------------------PAEV 201

ARATH2 --------------------------------------------------------PAKV 193

CHLRE --------------------------------------------------------PANV 180

Cucumber1 --------------------------------------------------------STNV 207

ARATH1 --------------------------------------------------------AANV 182

HOMO -------------------------------------------------------APHKY 159

MUS -------------------------------------------------------APHKY 158

SACCE -------------------------------------------------------APHKP 131

SCHPO -------------------------------------------------------APHLP 124

Ciliate -------------------------------------------------------APHIY 108

BACSU ------------------------------------------------------EITFKA 82

PLAFA SYGMRYGERSIKKGLEYLQKEN--INKLLVLPLYPQSAECTVSSTLDCIGKNLKNWSNVP 180

TOXO LMAMRYGEPSLPSVLREARKGG--CRKLLILPLYPQSAASTTSSVYDAAMQEIMKWRVMP 714

RHOCA DFCMRYGNPSTRDVLDDMLAQG--CERILFLPLYPQYAGATSATANDQFFRALMQVKRQP 179

AGRO DWAMRYGQPSIASRIDALKEQG--CEKILLFPLYPQYAASTTATVNDKAFEHLMKLRWQP 197

ECOLI ALGMSYGSPSLESAVDELLAEH--VDHIVVLPLYPQFSCSTVGAVWDELARILARKRSIP 154

YERMO ELGMSYGSPNLPDAIDKLLAQG--VTKLVVLPLYPQYSCSTSAAVWDAVARILKGYRRLP 154

NEIME RHAMTYGNPSVADVLSELKAQG--AGRLLVIPMYPQYAASSSGAAVDKVCEQLLLQRNQM 166

RALEU ACAMRYGNPSIASVLEALRRQG--TEQVLVLPMYPQYSGTTTATAFDEVFRVLGQWRNQP 173

HELPY TYAMRYTPPYASMVLQDLALKE--IESLVFFSMYPQYSSTTTLSSFNDAFNALKSLETFR 165

CYAME AVGMQFGEPNLVSVMHDFRRRG--IDRMIIVPMFPQYASSTTGSASEMAYHTASKLYSTP 235

THAPS YIGMRYWYPFTEEALAQIREDG--INALVILPLYPQFSISTSGSSLRVLQEEFARNSDFY 255

RICPR FINMRYSTPFAKEVIGQIKEYN--PSEIILLPLYPQFSSTTTGSSVKNFLQNIDID---- 149

Wolb FICMRYWHPFANEVVKSVKQFD--PDEVILLPLYPQYSTTTTLSSIENWQKNAKQYGIKC 176

wWill FICMRYWRPFADEVIESVKQFD--PDEVILLPLYPQYSTTTTLSSIENWQKNAKRYGLKC 151

wMel FICMRYWRPFADEVIESVKQFD--PDEVILLPLYPQYSTTTTLSSIENWQKNAKRYGLKC 151

wAna FICMRYWRPFADEVIESVKQFD--PDEVILLPLYPQYSTTTTLSSIENWQKNAKRYGLKC 151

CAUCR FIAMRYWHPLTDETARQVAAFA--PDQVVLLPLYPQFSTTTTGSSLKAWKKTYKGS---- 150

SYNE YIGMRYWHPFTEEAVEKIKGDR--LQRLVILPLYPHFSISTSGSSFRVLEEMWHNDPSLR 152

SYNC YVAMRYWHPFTESAVADMKADG--MDEVVVLPLYPHFSISTSGSSFRELQRLRQGDAAFE 152

Cucumber2 YVGMRYWHPFTEEAIEQIKKDG--ISKLVVLPLYPQFSISTSGSSLRLLEGIFREDEYLV 259

ARATH2 YVGMRYWHPFTEEAIEQIKRDG--ITKLVVLPLYPQFSISTSGSSLRLLERIFREDEYLV 251

CHLRE YVGMRYWHPYTEEALEHIKADG--VTRLVILPLYPQFSISTSGSSLRLLESLFKSDIALK 238

Cucumber1 YVGMRYWYPFTEEAIQQIKRDG--ITRLVVLPLYPQYSISTTGSSIRVLQKMFREDAYLS 265

ARATH1 YVGMRYWYPFTEEAVQQIKKDK--ITRLVVLPLYPQYSISTTGSSIRVLQDLFRKDPYLA 240

HOMO YIGFRYVHPLTEEAIEEMERDG--LERAIAFTQYPQYS**C**STTGSSLNAIYRYYNQVGRKP 217

MUS YIGFRYVHPLTEEAIEEMERDG--LERAIAFTQYPQYSCSTTGSSLNAIYRYYNEVGQKP 216

SACCE YVAFRYAKPLTAETYKQMLKDG--VKKAVAFSQYPHFSYSTTGSSINELWRQIKALDSER 189

SCHPO FVAFRYAPPLTEDMLDELKKAN--VSRAVAFSQYPQWSCATSGASLNELRRKLIEKGMEK 182

Ciliate FPAFRYGLPLYTESIKECIEKNPTVEKFVFFSQYPQYSCTTAGNNIREALKHLKEQYKNH 168

BACSU YIGLKHIEPFIEDAVAEMHKDG--ITEAVSIVLAPHFSTFSVQSYNKRAKEEAEKLGGLT 140

: . : . *: : :

PLAFA -----ELRFISGYCLKDIFINTMKENIENYWEL--------------------------- 208

TOXO -----DLRLLSGYADHPAYIAALAATVRRFWEAKETAKNARP--------------ESSG 755

RHOCA -----AARTVPEYFARPSYIEALASSVERVYATLDT------------------------ 210

AGRO -----AIRTVPPYHDDPAYIEGLAASVKNHLATLDW------------------------ 228

ECOLI -----GISFIRDYADNHDYINALANSVRASFAKHG------------------------- 184

YERMO -----SVSFIRDYAEHPTYISALKQSVERSFAQHG------------------------- 184

NEIME -----SVRTVSRFYDDTGYIDAMKNHILRYWAEHG------------------------- 196

RALEU E-----IRLVKHFHDHPAYIAALHQQVGAYWAQHG-----------------------TP 205

HELPY P----KVRVIERFYADKKLNEIILNTILSALNN--------------------------- 194

CYAME -----YLHIVPAFYDHPAYIQAYANVIERVIGPRGS------------------------ 266

THAPS GPQRMFHTVVPSWYDRPGYVKSVANLINKELESFTPEEIAEG---------------TSD 300

RICPR ----IPIKTICCYPIEEDFIKAHVSIIKEKLYD--------------------------- 178

Wolb N-----TKIIRHHYDNQDFIEAHANLITKHYKLAS------------------------- 206

wWill N-----TKMIHRYYDNQDFIEAHTNLIAKYYKLAR------------------------- 181

wMel N-----TKMIHRYYDNQDFIEAHTNLIAKYYKLAR------------------------- 181

wAna N-----TKMIHRYYDNQDFIEAHTNLIAKYYKLAR------------------------- 181

CAUCR ----GVQTTVGCYPTEGGLIEAHARMIRESWE--------------------------KA 180

SYNE Q---LDYSLIPSWYDHPGYLQAMADLIAQELK--------------------------KF 183

SYNC Q---LPIRCIRSWFDHPGYIKAMAELIAEEVR--------------------------NS 183

Cucumber2 N---MQHTVIPSWYQREGYIKAMADLIEKELK--------------------------TF 290

ARATH2 N---MQHTVIPSWYQREGYIKAMANLIQSELG--------------------------KF 282

CHLRE S---LRHTVIPSWYQRRGYVSAMADLIVEELK--------------------------KF 269

Cucumber1 S---LPVSIIKSWYQREGYIKSMADLMQAELK--------------------------NF 296

ARATH1 G---VPVAIIKSWYQRRGYVNSMADLIEKELQ--------------------------TF 271

HOMO T---MKWSTIDRWPTHHLLIQCFADHILKELD------------------------HFPL 250

MUS T---MKWSTIDRWPTHPLLIQCFADHILKELN------------------------HFPE 249

SACCE S---ISWSVIDRWPTNEGLIKAFSENITKKLQ------------------------EFPQ 222

SCHPO D---FEWSIVDRWPLQQGLINAFAENIEETLK------------------------TYPE 215

Ciliate G---KTIHVIDRWYNHPGYVKTISRLLSEDLKNNFKEVCSKINFIVEKQKYLFQQNKSEQ 225

BACSU ------ITSVESWYDEPKFVTYWVDRVKETYAS------------------------MPE 170

: :

PLAFA -YGKSKKLIISYHSLPIRNVI-QGDL---------------------YPFFCIESTKKLV 245

TOXO ARGRGEKLIFSFHGIPLNTGRQAGEI---------------------YQCLCAKTARLTA 794

RHOCA ---RPDVLVASYHGMPKRYHR-EGDP---------------------YHCQCQKTSRLLR 245

AGRO ---EPEMLITSFHGIPQSYFK-KGDP---------------------YYCHCQKTARLLR 263

ECOLI ---EPDLLLLSYHGIPQRYAD-EGDD---------------------YPQRCRTTTRELA 219

YERMO ---KPDRLVMSFHGIPKRYAQ-LGDD---------------------YPIRCEETSRALQ 219

NEIME ---RGKKLMLSFHGVPQKHHD-LGDP---------------------YPDECRHTAKLLA 231

RALEU DFARGDKLILSFHGVPRRTLE-LGDP---------------------YHCECLKTGRLLG 243

HELPY CKSQDFVLIFSVHGLPKSIVD-AGDT---------------------YQQECEHHVSLLK 232

CYAME --RRVDHLLLSFHGVPEKHCQQTDDTGLVCMQQEHCCSALVQANRNCYRAQCFATARALA 324

THAPS GQPIPKHVLFSAHGVPASYIE-AGDP---------------------YKDQIIDCVERIS 338

RICPR ---KNFRILFSAHGLPKRIIK-AGDP---------------------YSFQIKETVNKIV 213

Wolb -EVGKPRVLFSAHSLPLSVIK-KGDP---------------------YALQVEETVKLIV 243

wWill -KIGKPRVLFSAHSLPLSIIK-KGDP---------------------YASQVERSVELIV 218

wMel -KIGKPRVLFSAHSLPLSIIK-KGDP---------------------YASQVERSVELIV 218

wAna -KIGKPRVLFSAHSLPLSIIK-KGDP---------------------YASQVERSVELIV 218

CAUCR GSPTNIRLLFSAHGLPEKVIL-AGDP---------------------YQKQVEATAAAVA 218

SYNE PNPDQAHIFFSAHGVPQSYVDEAGDP---------------------YQAEIEACTRLIM 222

SYNC DDPEKAHVFFSAHGVPKSYVEEAGDP---------------------YQQQIEACTDLIM 222

Cucumber2 DFPEQVMVFFSAHGVPLAYVEEAGDP---------------------YKAEMEECVDLIM 329

ARATH2 GSPNQVVIFFSAHGVPLAYVEEAGDP---------------------YKAEMEECVDLIM 321

CHLRE RDVPSVELFFSAHGVPKSYVEEAGDP---------------------YKEEMEECVRLIT 308

Cucumber1 ANPQEVMIFFSAHGVPVSYVENAGDP---------------------YKDQMEECICLIM 335

ARATH1 SDPKEVMIFFSAHGVPVSYVENAGDP---------------------YQKQMEECIDLIM 310

HOMO EKRSEVVILFSAHSLPMSVVNR-GDP---------------------YPQEVSATVQKVM 288

MUS EKRSEVVILFSAHSLPMSVVNR-GDP---------------------YPQEVGATVHKVM 287

SACCE PVRDKVVLLFSAHSLPMDVVNT-GDA---------------------YPAEVAATVYNIM 260

SCHPO DVRDDVVIVFSAHSLPMSQVAK-GDP---------------------YVYEIAATSQAVM 253

Ciliate EDRDNVLILFSAHSLPFDFVKQ-GDT---------------------YPYEIGTTANLVI 263

BACSU DERENAMLIVSAHSLPEKIKEF-GDP---------------------YPDQLHESAKLIA 208

:. * *.:* .: *

PLAFA KSLNLN---KDDYILVFQSKIKGQ--QWVKPCIEDTI-IRLAKQGYKQIDIVSPSFSSDC 299

TOXO EQLQLK---PEEFEVAFQSRFGPA--EWTQPYMDKRL-EALAVAGYRFVDVVMPGFATDC 848

RHOCA ERLGWG---PDSIDTTFQSVFGTE--EWLRPYTVEHV-VQLAEAGKKNIAVISPAFSADC 299

AGRO EALGRT---EKNFMITFQSRFGPE--EWLQPYTDKTV-EKLASEGIKRIAVMNPGFVSDC 317

ECOLI SALGMA---PEKVMMTFQSRFGRE--PWLMPYTDETL-KMLGEKGVGHIQVMCPGFAADC 273

YERMO AELTLP---PEQIMMTYQSRFGRE--PWLTPYTDETL-KNLPAQGVEHIQLICPGFSADC 273

NEIME EALELT---EDQYVVSFQSQFGRA--KWVTPSTQDLF-GKLPKQGVTELDVFCPGFLADC 285

RALEU DALGLQ---PGQYQVTFQSRFGKA--EWLQPYTAPTL-AELGKVGAGRVDVFCPGFPADC 297

HELPY ELMQQKNIPFKEVLLSYQSKLGPM--KWLEPSTEELI----EKHRKSNIIIYPLAFTIDN 286

CYAME QTLGLA---PHEYSVSFQSRLTAAGPEWIKPYTDEVL-TILPKQGVRRLAVAIPSFVTDC 380

THAPS ALLPSE-EDGVKVHLSFQSRVGPV--EWLRPYTDDVL-PSLGEQGVKNLVVVPISFVSEH 394

RICPR KELNIK---DLDYKITYQSRVGPI--EWLKPNTEDEI-ELAGKL-KKDIIIVPISFVSEH 266

Wolb KKLHIK---DLDWSICYQSKIGPV--KWLEPSTESEL--LRAKADGVPVVLLPISFVSEH 296

wWill EKLAIN---NLDWSICYQSKIGPV--KWLEPSTESEL--LRAKADGVPVVLSPISFVSEH 271

wMel EKLAIN---NLDWSICYQSKIGPV--KWLEPSTESEL--LRAKADGVPVVLSPISFVSEH 271

wAna EKLAIN---NLDWSICYQSKIGPV--KWLEPSTESEL--LRAKADGVPVVLSPISFVSEH 271

CAUCR AHLPP----QIEWTVCYQSRVGPL--KWIGPSTDDEI-RRAGGE-DKGVMITPIAFVSEH 270

SYNE RTLDR----PNQYTLAYQSRVGPV--EWLKPYTEEAL-QKLGAEGIDDLLVVPISFVSEH 275

SYNC KSLAEHMGHSNPHTLAYQSRVGPV--EWLKPYTEEAL-EQLGEAKTNDLVVVPISFVSEH 279

Cucumber2 EELEKR-RITNSYTLAYQSRVGPV--EWLKPYTDETI-IELGQKGVKSLLAVPISFVSEH 385

ARATH2 EELDKR-KITNAYTLAYQSRVGPV--EWLKPYTEEAI-TELGKKGVENLLAVPISFVSEH 377

CHLRE DEVKRR-GFANTHTLAYQSRVGPA--EWLKPYTDESI-KELGKRGVKSLLAVPISFVSEH 364

Cucumber1 QELKAR-GIGNEHTLAYQSRVGPV--QWLKPYTDEVL-VELGQKGIKSLLAVPVSFVSEH 391

ARATH1 EELKAR-GVLNDHKLAYQSRVGPV--QWLKPYTDEVL-VDLGKSGVKSLLAVPVSFVSEH 366

HOMO ERLEYC----NPYRLVWQSKVGPM--PWLGPQTDESI-KGLCERGRKNILLVPIAFTSDH 341

MUS EKLGYP----NPYRLVWQSKVGPV--PWLGPQTDEAI-KGLCERGRKNILLVPIAFTSDH 340

SACCE QKLKFK----NPYRLVWQSQVGPK--PWLGAQTAEIA-EFLGPKVDG-LMFIPIAFTSDH 312

SCHPO KRLNYK----NKFVNAWQSKVGPL--PWMSPATDFVI-EQLGNRGQKNMILVPIAFTSDH 306

Ciliate QEAKLK----NPHRVVWQSKVGFQ--QWLAPNTMHAL-EQASNQGWKNVILVPLGFTSDH 316

BACSU EGAGVS-----EYAVGWQSEGNTP-DPWLGPDVQDLTRDLFEQKGYQAFVYVPVGFVADH 262

:** * . . .* :

PLAFA LETLEEIKIHYQQLFRKYSNGN--LRYINCLNDTTIGIKLIMNLIEQNIIGWV------- 350

TOXO LETIEEMASVYRGKFLELTHGEGELRVLPCLNASEEATAAVFAIAREQMTDWLKLMVPLE 908

RHOCA IETLEEINGEIREAFEHAGGES--FTYVPCLNDDDLHIAALLEVVEENLAGWID------ 351

AGRO LETLEEIAGEAGEIFLHNGGEK--FTHIPCLNDSTEGMNVLEKVVRRELQGWV------- 368

ECOLI LETLEEIAEQNREVFLGAGGKK--YEYIPALNATPEHIEMMANLVAAYR----------- 320

YERMO LETLEEIKEQNREIFLHAGGKK--FEYIPALNDDVGHIDLLEQLVREYM----------- 320

NEIME LETMEEIALMGREQFYEAGGKS--YRYIPCLNDNPDWIDALVALAEENLGSWR------- 336

RALEU IETLEEIAMEGQTEFMVAGGKT--FHFIPCMNDAQPWISALAEIALQHVQGWPLN----- 350

HELPY SETLYELDMQYR--LMAERLAVKEYLVCPCLNDSIEFAKFIIELVKNLKSE--------- 335

CYAME LETLEEIGMQGREEFLNAGGEE--FYLVPCLNDSQEWADALLRIIEDSC----------- 427

THAPS IETLEEIDIEYR--ELALESGITNWRRSPALNTDATFIEDMADMVADALNEPSQS----- 447

RICPR VETLVELDIEYK--LIADKY-KIQYTRIPTLGTNKIFINSLTNILLRFINNTNTN----- 318

Wolb SETLVELDMEYK-----TIIKDGYYFRIPTLSTNSLFIKCLAGLCINHS----------- 340

wWill SETLVELDIEYK-----AIIKDGYYFRVPTLSTDPLFIKCLADLCINLP----------- 315

wMel SETLVELDIEYK-----AIIKDGYYFRVPTLSTDPLFIKCLADLCINLP----------- 315

wAna SETLVELDIEYK-----AIIKDGYYFRVPTLSTDPLFIKCLADLCINLP----------- 315

CAUCR VETLVELDHEYA--ELAEEVGAAPYLRVSALGTAPEFIDGLAKAVRDSVGKAPG------ 322

SYNE IETLQEIDIEYR--EIAEEAGIDNFQRVPALNTHPVFIDALAQMVMDSLNDP-------- 325

SYNC IETLEEIDIEYR--ELATEAGVVNFRRVRALDTYPPFIEGLADLVTTSLEGP-------- 329

Cucumber2 IETLEEIDVEYK--ELALKSGIEKWGRVPALGCEPTFITDLADAVIESLPYVGAMAVSNL 443

ARATH2 IETLEEIDVEYK--ELALKSGIKNWGRVPALGTEPMFISDLADAVVESLPYVGAMAVSNL 435

CHLRE IETLEEIDMEYR--ELAEESGIRNWGRVPALNTNAAFIDDLADAVMEALPYVGCLAGP-- 420

Cucumber1 IETLEEIDMEYK--HLALESGIQNWGRVPALNCNSSFISDLADAVIEALPSA-------- 441

ARATH1 IETLEEIDMEYR--ELALESGVENWGRVPALGLTPSFITDLADAVIESLPSA-------- 416

HOMO IETLYELDIEYSQ-VLAKECGVENIRRAESLNGNPLFSKALADLVHSHIQSN-------- 392

MUS IETLYELDIEYSQ-VLAQKCGAENIRRAESLNGNPLFSKALADLVHSHIQSN-------- 391

SACCE IETLHEIDLG----VIGESEYKDKFKRCESLNGNQTFIEGMADLVKSHLQSN-------- 360

SCHPO IETLKELEDYI---EDAKQKGITGVKRVSSINGSMTAIQGMADLVAEHLKAK-------- 355

Ciliate LETLYELDLEYIK-ESQEKLKFNKIIRARSLNDDQQFCDSLADIVSQNLKTD-------- 367

BACSU LEVLYDNDYECK---VVTDDIGASYYRPEMPNAKPEFIDALATVVLKKLGR--------- 310

*.: : . :

PLAFA ------------------------------------------------------------

TOXO GEKNP------------------------------------------------------- 913

RHOCA ------------------------------------------------------------

AGRO ------------------------------------------------------------

ECOLI ------------------------------------------------------------

YERMO ------------------------------------------------------------

NEIME ------------------------------------------------------------

RALEU -------------------------------------MPHAHELEARRSRAQTRGAAA-- 371

HELPY ------------------------------------------------------------

CYAME ------------------------------------------------------------

THAPS ---------------VTEACVANNVGNLELEAVSNRMEISSELINGRLAMMGIFVTILLE 492

RICPR -----------------------------------LVMSSSSKRICPNKFTKCLCNLTN- 342

Wolb ------------------------------------------------------------

wWill ------------------------------------------------------------

wMel ------------------------------------------------------------

wAna ------------------------------------------------------------

CAUCR ------------------------------------TVSSACGWRCGADWSKCPCREGAS 346

SYNE --PCTFETVPHPKKNMKMYPQER---------WEWGLTTAAEVWNGRLAMLG-FIALLVE 373

SYNC --EVSLDAAAELPTKVKLYPQEK---------WEWGWNNSSEVWNGRLAMLG-FSAFLLE 377

Cucumber2 EARQPLVPLGSVEELLAAYDSQRRQLPPPVTVWEWGWTKSAETWNGRAAMLAVLVLLVLE 503

ARATH2 EARQSLVPLGSVEELLATYDSQRRELPAPVTMWEWGWTKSAETWNGRAAMLAVLALLVLE 495

CHLRE --TDSLVPLGDLEMLLQAYDRERRTLPSPVVMWEWGWTKSAETWNGRIAMIAIIIILALE 478

Cucumber1 ------TALAP---HTSSTDADDHDPFLYAIKLLFGSVLAFILLLSPKAFMVFRNNFLLN 492

ARATH1 ------EAMSNPNAVVDSEDSESSDAFSYIVKMFFGSILAFVLLLSPKMFHAFRNL---- 466

HOMO -----------------------------------EL**C**SKQLTLSCPL**C**VN--PV**C**RETK 415

MUS -----------------------------------KLCSTQLSLNCPLCVN--PVCRKTK 414

SACCE -----------------------------------QLYSNQLPLDFALGKSNDPVKDLSL 385

SCHPO -----------------------------------VPYSRQFTQRCPGCTS--ESCAERI 378

Ciliate -----------------------------------AYKSPNLKLRCPDCKI--PECNLLS 390

BACSU ------------------------------------------------------------

PLAFA ----------------------

TOXO ----------------------

RHOCA ----------------------

AGRO ----------------------

ECOLI ----------------------

YERMO ----------------------

NEIME ----------------------

RALEU ----------------------

HELPY ----------------------

CYAME ----------------------

THAPS FASGKPLIHFFSW--------- 505

RICPR ----------------------

Wolb ----------------------

wWill ----------------------

wMel ----------------------

wAna ----------------------

CAUCR A--------------------- 347

SYNE LISGQGPLHFVGLL-------- 387

SYNC LISGHGPLHALGLL-------- 391

Cucumber2 VTTGEGFLHQWGIFPLFHQ--- 522

ARATH2 VTTGKGFLHQWGILPSL----- 512

CHLRE AASGQSILKNLFLAE------- 493

Cucumber1 YTRIYGYRGERSEFFWVRLIFT 514

ARATH1 ----------------------

HOMO SFFTSQQL-------------- 423

MUS SFFTSQQL-------------- 422

SACCE VFGNHEST-------------- 393

SCHPO NFFQDF---------------- 384

Ciliate AV-------------------- 392

BACSU ----------------------
